# Optimization of TAM16, a Benzofuran That Inhibits the Thioesterase Activity of Pks13; Evaluation toward a Preclinical Candidate for a Novel Antituberculosis Clinical Target

**DOI:** 10.1021/acs.jmedchem.1c01586

**Published:** 2021-12-15

**Authors:** Caroline Wilson, Peter Ray, Fabio Zuccotto, Jorge Hernandez, Anup Aggarwal, Claire Mackenzie, Nicola Caldwell, Malcolm Taylor, Margaret Huggett, Michael Mathieson, Dinakaran Murugesan, Alasdair Smith, Susan Davis, Mattia Cocco, Maloy K. Parai, Arjun Acharya, Fabio Tamaki, Paul Scullion, Ola Epemolu, Jennifer Riley, Laste Stojanovski, Eva Maria Lopez-Román, Pedro Alfonso Torres-Gómez, Ana Maria Toledo, Laura Guijarro-Lopez, Isabel Camino, Curtis A. Engelhart, Dirk Schnappinger, Lisa M. Massoudi, Anne Lenaerts, Gregory T. Robertson, Chris Walpole, David Matthews, David Floyd, James C. Sacchettini, Kevin D. Read, Lourdes Encinas, Robert H. Bates, Simon R. Green, Paul G. Wyatt

**Affiliations:** † Drug Discovery Unit, Division of Biological Chemistry and Drug Discovery, College of Life Sciences, University of Dundee, Dundee DD1 5EH, U.K.; ‡ Department of Biochemistry and Biophysics, Texas A&M University, College Station, Texas 77843, United States; § Global Health Pharma R&D, GlaxoSmithKline, Severo Ochoa 2, Tres Cantos, Madrid 28760, Spain; ∥ Department of Microbiology and Immunology, Weill Cornell Medical College, New York, New York 10065, United States; ⊥ Mycobacteria Research Laboratories, Department of Microbiology, Immunology, and Pathology, Colorado State University, 200 W. Lake Street, Fort Collins, Colorado 80523-1682, United States; # Structural Genomics Consortium, Research Institute of the McGill University Health Centre, 1001 Boulevard Décarie, Site Glen Block E, ES1.1614, Montréal, Québec H4A 3J1, Canada

## Abstract

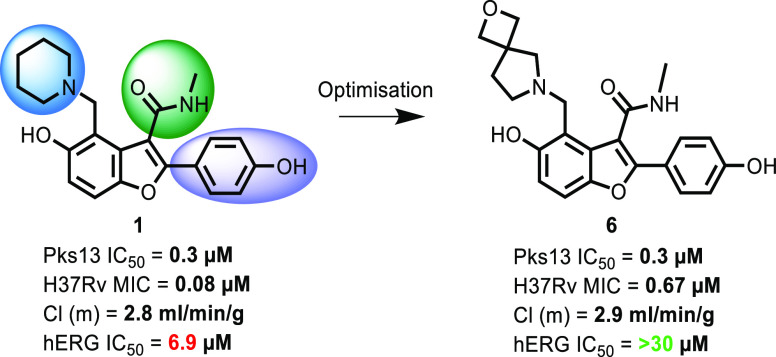

With increasing drug resistance in tuberculosis (TB) patient populations, there is an urgent need for new drugs. Ideally, new agents should work through novel targets so that they are unencumbered by preexisting clinical resistance to current treatments. Benzofuran **1** was identified as a potential lead for TB inhibiting a novel target, the thioesterase domain of Pks13. Although, having promising activity against *Mycobacterium tuberculosis*, its main liability was inhibition of the hERG cardiac ion channel. This article describes the optimization of the series toward a preclinical candidate. Despite improvements in the hERG liability in vitro, when new compounds were assessed in ex vivo cardiotoxicity models, they still induced cardiac irregularities. Further series development was stopped because of concerns around an insufficient safety window. However, the demonstration of in vivo activity for multiple series members further validates Pks13 as an attractive novel target for antitubercular drugs and supports development of alternative chemotypes.

## Introduction

Before the COVID-19 global pandemic, in 2019, tuberculosis (TB) was the leading cause of death worldwide from a single infectious agent (*Mycobacterium tuberculosis*), killing 1.4 million people (1.2 million HIV-negative and 0.2 million HIV-positive).^1^ Current front-line therapy for TB involves 6 months’ treatment, using a cocktail of four drugs for the initial 2 months. Treatment must be completed to achieve disease sterilization in patients; however, the lengthy course of therapy leads to poor compliance, which is one of the key drivers of drug resistance.^2,3^ Globally, an estimated 3% of new TB cases and 18% of previously treated cases have multidrug-resistant TB (MDR-TB).^1^ In previous years, an estimated 6% of MDR cases were extensively drug-resistant TB, defined as MDR-TB plus resistance to at least two of the second-line therapies (a fluoroquinolone and one of the injectable agents such as capreomycin or kanamycin).^4^ Treatment of MDR-TB can last for up to 2 years and can have severe side effects leading to increased patient noncompliance. As a consequence, there is a clear and pressing need for novel treatment-shortening drugs to provide more effective TB treatment.^5−8^

The majority of pathogenic organisms have complex and different cell wall architecture compared to that of higher eukaryotes. As such, drugs targeting cell wall synthesis have been historically very effective in treating bacterial and fungal infections. *M. tuberculosis* is no exception; it contains a waxy outer cell-wall layer consisting of extended long-chain fatty acids called mycolic acids. These mycolic acids are a characteristic of the genus *Mycobacterium* and are known to be critical for the pathogenicity, virulence, and survival of *M. tuberculosis*.^9^ The disruption of mycolic acid biosynthesis has been exploited in the current armory of antitubercular agents. Both isoniazid and ethionamide target InhA, one of the fatty acid synthetase II enzymes participating in the elongation of the long-chain (C40–C60) fatty acid components of mycolic acids. Because of the effectiveness of cell wall-targeting agents, there has been a continuous quest for novel agents that inhibit these enzymes or other enzymes involved in mycolic acid biosynthesis.^10^

Polyketide synthase 13 (Pks13) was identified as the enzyme responsible for the last stage of mycolic acid synthesis, the condensation of two fatty acid chains into an α-alkyl β-ketoacyl chain, a direct precursor of the mycolates.^11,12^ Pks13 contains the catalytic domains required for this condensation reaction: an acyl transferase domain, a ketosynthase domain, acyl carrier protein (ACP) domains, and a thioesterase (TE) domain.^11^ Pks13 is essential for mycobacterial survival^11,13,14^ and therefore represents an attractive new target for the potential discovery of novel antituberculosis agents. Phenotypic screening has recently identified two series of compounds that potently inhibit the growth of *M. tuberculosis* by targeting Pks13. A single nucleotide polymorphism (SNP) converting Phe 79 to Ser within the N-ACP domain of Pks13 conferred resistance to a novel thiophene, TP2,^15^ while, in a similar manner, two different SNPs within the TE domain of Pks13 (D1607N & D1644G) conferred resistance to a novel benzofuran phenotypic hit.^16^ These two reports confirm that Pks13 appears to be an excellent target for the identification of novel agents to inhibit the growth of *M. tuberculosis*. Based on the initial benzofuran starting point, a hit to lead (H2L) program was initiated to understand any limitations of the primary hit compound. As the target was already known, the H2L program was developed as a structure-based drug-design project using an in vitro assay to monitor Pks13 TE activity and an X-ray crystallography platform to generate structural information related to the TE domain.^17^ The H2L campaign identified TAM16 (**1**) as an early lead with improved potency and microsomal clearance; **1** demonstrated very good in vivo efficacy in murine models of TB infection.^17^

Although this early lead had very promising in vivo activity, we initiated a lead optimization program, investigating potential liabilities that could preclude its progression to the clinic. These included off-target activities and the two hydroxyl groups, with their potential for glucuronidation, a possible in vivo metabolic liability. Herein, we describe the lead optimization program for compound **1**. In the initial H2L publication, substitutions on the benzofuran were classified as piperidine P^3^, amide P^2^, and phenol P^1^;^17^ a consistent nomenclature will be used in this report.

## Chemistry

### Synthetic Routes

**Scheme 1:**
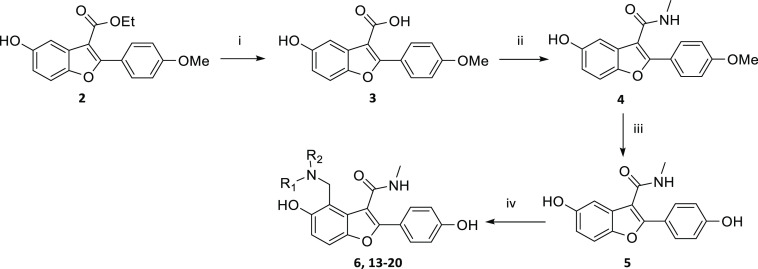
General Route to Synthesis of P^3^ Modifications *Reagents and conditions:* (i) NaOH, EtOH, H_2_0, 80 °C; (ii) MeNH_2._HCl, EDCI, pyridine, 15 °C; (iii) BBr_3_, DCM, 0 to 25 °C, N_2_; (iv) Formaldehyde, amine, AcOH, THF, microwave, 75 or 80 °C, or formaldehyde, amine, dioxane/water, 50 °C, or formaldehyde, amine, DIPEA, EtOH, H_2_0, 80 °C/Reflux or microwave, 120 °C.

To explore the SAR at P^3^, the synthesis of several analogues was achieved according to the route outlined in Scheme 1. Compound **2** was synthesized using a literature procedure.^17^ Carboxylic acid **3** was formed from hydrolysis of ester **2** and was then converted to amide **4**. The methoxy group of **4** was converted to the phenol **5** by reaction with boron tribromide in DCM. The Mannich reaction with **5** yielded **6** and **13–20**. Other routes and the experimental details for individual compounds are described in the Supporting Information.

**Scheme 2:**
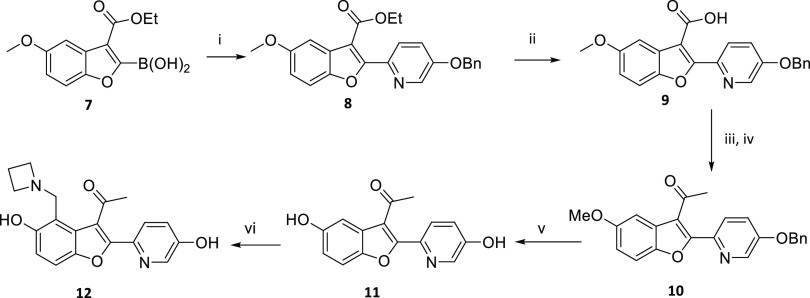
Synthesis of Compound **12** *Reagents and conditions:* (i) 5-(benzyloxy) −2-bromopyridine, K_2_CO_3_, Pd(dppf)Cl_2_, 1,4-dioxane, H_2_0, 80 °C; (ii) NaOH, EtOH, H_2_0, 80 °C; (iii) *N*-methoxymethanamine hydrochloride, HATU, DIPEA, DMF, rt.; (iv) MeLi, THF, −78 °C; (v) BBr_3,_ DCM, −78 °C to rt., N_2_; (vi) formaldehyde, azetidine, THF, H_2_0, 70 °C.

An example of a route to change substituents at P^3^, P^2^, and P^1^ is illustrated in Scheme 2 showing the synthesis of compound **12.** Compound **7** was synthesized using a literature procedure.^18^ A Suzuki reaction^19^ with compound **7** yielded the benzyl-protected pyridinol **8**. Hydrolysis of the ester of **8** gave the carboxylic acid **9**. The carboxylic acid of **9** was converted to the Weinreb amide and the methyl ketone **10** was formed by reaction of the Weinreb amide with methyl lithium in THF. The methoxy and the benzyloxy of **10** were converted to the phenols by reaction with boron tribromide in DCM. The Mannich reaction with **11** yielded **12**.

## Results and Discussion

**Figure 1:**
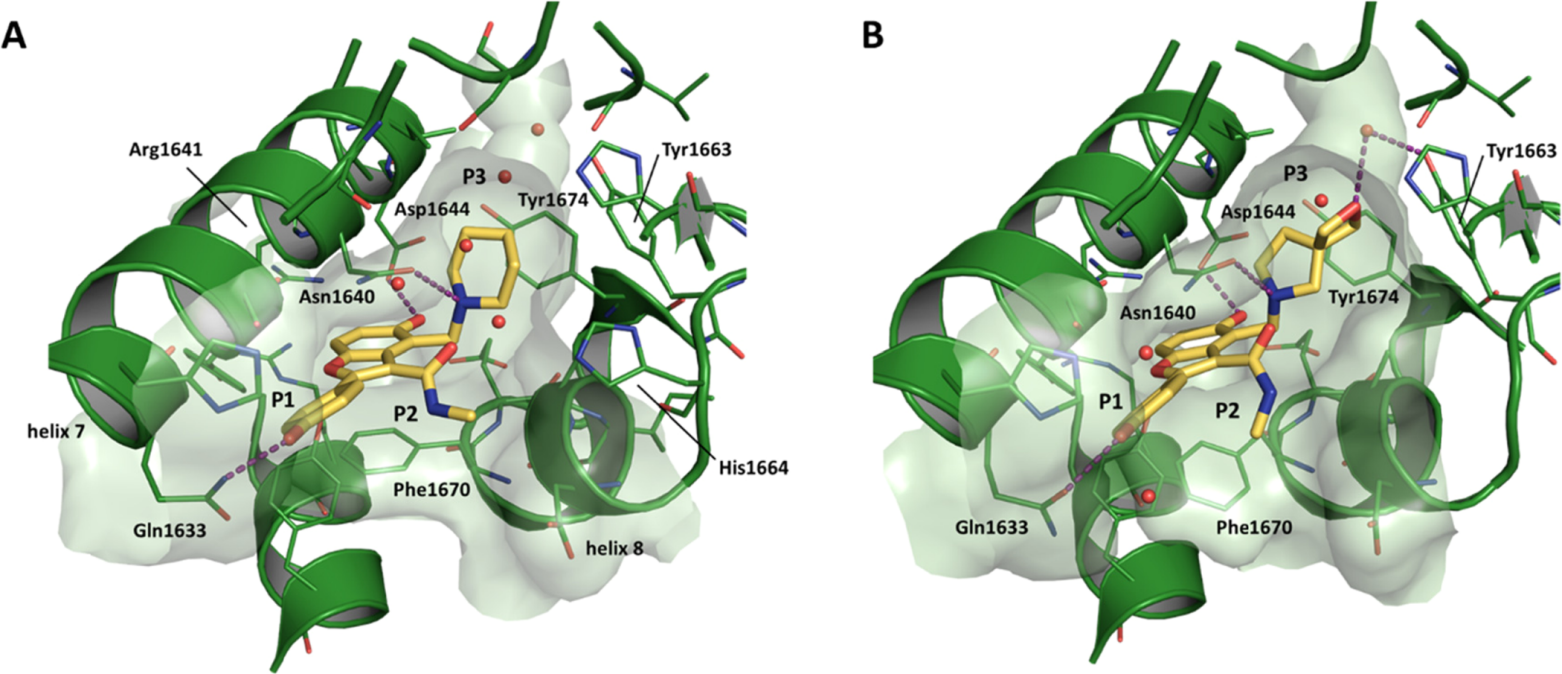
Crystal structure comparison for compounds **1** and **6** bound in the Pks13 TE domain. See text for details (**A)** compound **1** (PDB ID: 5V3Y); (**B)** compound **6** (PDB ID: 7M7V).

Compound **1** was synthesized originally during an H2L program^17^ based on an initial phenotypic hit that had been shown to target Pks13.^16^ Structural information generated during the H2L program identified the following interactions between Pks13 protein and **1**;^17^ after a ligand-induced rearrangement, **1** bound in the fatty acyl substrate binding groove (Figure 1), blocking access to the Pks13 TE active site where the catalytic triad (Ser1533, Asp1560, and His1699) is located. To accommodate the ligand, the helix α8 backbone moved 2 Ǻ outward compared to the apo form and the phenyl ring of the Phe1670 side chain, at the end of helix α8, rotated to stack against the benzofuran moiety of the ligand. The side chain of Arg1641 in helix α7 also moved to accommodate the ligand, making a stabilizing interaction with the side chain of Asp1607 of helix α6. The movement of Arg1641 permitted the Asp1644 backbone to move 2.8 Ǻ; its carboxylic group establishes a key interaction with the phenol hydroxyl on the benzofuran ring. One of the resistant mutants to the initial hit (D1644G) would eliminate this key interaction.^16^ The piperidine group at P^3^ has a calculated p*K*_a_^20^ of 10.2, and so at physiological pH, the molecule is positively charged. The piperidine moiety was positioned closest to the catalytic binding site, stacked between the aromatic rings of Tyr1663 and Tyr1674. The protonated nitrogen of the piperidine established a key hydrogen bond with the carbonyl oxygen of the Asn1640 side chain. The methyl amide in position P^2^ did not interact directly with the protein but there was a water-mediated interaction between the carbonyl oxygen and the side chain of His1664. The carbonyl oxygen also established an internal hydrogen bond with the protonated piperidine nitrogen contributing to the stabilization of the bioactive conformation. The phenol group in P^1^ was largely solvent-exposed with the *para* hydroxyl forming a hydrogen bond with the Gln1633 side chain.

As the early lead **1** looked very promising (Table 1), the focus of the studies reported herein was to optimize the all-round properties of this series to ensure the identification of a candidate suitable for clinical development. Particular attention was paid to off-target human Ether-à-go-go-Related Gene (hERG) ion channel activity (IC_50_), which was determined to be ∼7 μM (Table 1). Inhibition of the hERG ion channel is a common cause of in vivo cardiac toxicity that can result in termination of development of a chemical series if it cannot be mitigated. The hERG channel is blocked by a surprisingly diverse group of drugs, many of which, like **1**, are lipophilic amines.^21^ To most efficiently use synthetic chemistry resources and to guide optimization of compound **1**, the binding cavity of Pks13 was analyzed to identify potential interaction hotspots, and the designed compounds were evaluated in silico by molecular docking prior to synthesis.

**Table 1:** Profile of Initial Lead **1**

	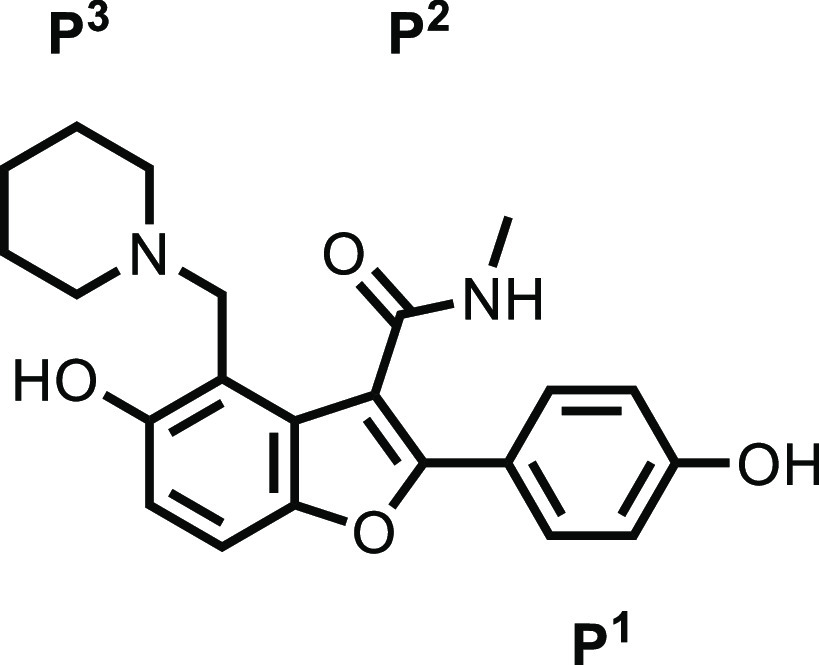
Pks13 IC_50_^a^	0.32 μM
H37Rv MIC^b^	0.08 μM
InMac MIC_90_^c^	0.01 μM
In vivo acute ED_99_^d^	13 mg/kg
hERG Q Patch^e^	6.9 μM
HepG2 IC_50_^f^	>100 μM
microsomal clearance^g^	2.8 mL/min/g
CHI Log*D*_pH7.4_^h^	1.8
aqueous solubility^i^	>250 μM
PAMPA (Pe)^j^	5.2 nm/s
*M*_w_^k^	380
TPSA^l^	86

a Pks13 IC_50_*M. tuberculosis* Pks13 TE domain 50% inhibitory concentration (μM).

b H37Rv MIC is the minimum concentration required to inhibit the growth of *M. tuberculosis* strain H37Rv in liquid culture.

c InMac MIC_90_ is the concentration required to inhibit 90% of the luminescent signal from a luciferase-expressing *M. tuberculosis* strain growing in THP1 monocytes.

d In vivo acute ED_99_ dose that causes a 2 log_10_ reduction in colony-forming units with respect to untreated mice.

e hERG Q-patch 50% inhibitory concentration (μM).

f HepG2 IC_50_ is an assessment of cytotoxicity in the HEPG2 human liver cancer cell line.

g Intrinsic microsomal clearance (Cli) using CD1 mouse liver microsomes.

h CHI-LogD_pH7.4_ is a measure lipophilicity at pH 7.4.

i Aqueous solubility is kinetic aqueous solubility.

j PAMPA Parallel artificial membrane permeability assay.

k Molecular weight.

l TPSA (Å^2^) is the total polar surface area.

### P^3^ SAR

The pocket where P^3^ binds is the most buried pocket and plays a significant role in molecular recognition. The piperidine group only partially occupies the cavity that extends into the TE catalytic site (Figure 1). An analysis of the binding pocket showed two water molecules located deep into the catalytic site that sit in a hydrophobic environment and are characterized by a low interaction energy. A third water molecule, closer to the ligand (4.2 Ǻ from the carbon atoms of the piperidine ring), is located in an area of favorable polar interactions and is stabilized by a hydrogen bond with the hydroxyl side chain of Tyr1663. Most of the carbon atoms of the piperidine ring match a favorable hydrophobic hotspot, whereas the carbon adjacent to the charged nitrogen atom faces the negatively charged Asp1644 side chain. The protonated piperidine nitrogen is important in establishing a H-bond interaction with Asn1640 and in stabilizing the bioactive conformation.^17^ However, the basic lipophilic amine was also the most likely contributor to the off-target hERG signal.^21^ Therefore, modification of the piperidine was a focus of the P^3^ SAR; the strategy focused on lowering log*D*_pH7.4_ and reducing the predicted p*K*_a_ of the amine to try and decrease inhibition of the hERG ion channel, while retaining excellent inhibition of the Pks13 TE domain.

It was known that removal of the ionizable group by exchanging the piperidine for either a cyclohexyl or phenyl group resulted in a significant drop in both Pks13 inhibition and growth inhibition.^17^ Thus, more modest changes were explored, that retained the amine but were designed to improve overall properties. Replacement of the piperidine **1** with a pyrrolidine **13** or an azetidine ring **14** to lower the log*D*_pH7.4_ was tolerated, but these molecules still retained activity against the hERG ion channel. The 3-*R*-hydroxy piperidine **15**, despite being potent in the Pks13 assay, had reduced MIC activity. The 1-(aminomethyl) cyclopropan-1-ol **16** and the 2-oxa-6-azaspiro[3.4]octane **6** were potent in the Pks13 assay and had good MIC activity and microsomal stability, with reduced activity in the hERG assay. Both modifications decreased the log*D*_pH7.4_ and amine p*K*_a_ (predicted p*K*_a_ was 9.4 and 9.2 respectively) so were considered worthy of further investigation. Various other spiro groups were explored, these usually retained activity in the Pks13 assay, but all had reduced MIC activity (**17–20**). Attempts to reduce the basicity of the piperidine nitrogen by introducing fluorine substitutions (**21–22**) had a detrimental impact on Pks13 potency and MIC; further confirming the importance of the interaction between the protonated N and Asn1640 in Pks13. To summarize the SAR at the P^3^ position, reducing the log*D*_pH7.4_ was tolerated in the Pks13 assay and some of these compounds retained sub μM MIC potency with decreased inhibition of the hERG channel inhibition. Reducing the basicity of the piperidine nitrogen by introducing fluorine substituents was detrimental; this was not entirely unexpected given the key interaction for ligand binding between the protonated nitrogen and Asn1640.

Compounds **1, 13, 16**, and **6** were selected for analysis in a murine acute TB efficacy study (results summarized in Table 6). During dosing, blood samples were taken (0.5, 1, 6, and 24 h) to obtain a preliminary evaluation of drug exposure. Of the compounds evaluated, the original lead **1** was the most potent giving a 3.9 log reduction in colony-forming units (CFU). Both **13** and **6** had good efficacy and were considered for follow-up, but later **13** was deprioritized because it retained some of the hERG channel inhibition seen with **1**. Compound **16**, although having promising properties, failed to show in vivo efficacy. The lack of in vivo activity appeared to be directly related to the very poor exposure achieved with this molecule, both in terms of *C*_max_ and area under the curve (AUC) (Table 6).

Because Pks13 crystals were available to guide the synthetic program, several ligand/Pks13 TE complexes were generated by crystal soaking as the project progressed. As **6** was the most promising molecule from the assessment of the P^3^ SAR, a crystal structure was obtained for the **6**/Pks13 TE complex (Figure 1 and Table S1). Compound **6** made the same key interactions with the Pks13 TE domain as **1** (Figure 1); these included a hydrogen bond between the hydroxyl of the P^1^ phenol and the amide side chain of Gln1633; a hydrogen bond between the hydroxyl at the 5 position of the benzofuran core with the carboxylic acid side chain of Asp1644; and a hydrogen bond between the protonated N of the P^3^ substituent and the side chain oxygen of Asn1640. There were also van der Waal interactions between the P^3^ substituents and the phenol sidechains of Tyr1663 and Ty1674. In compound **6**, the 2-oxa-6-azaspiro[3.4]octane group reached further than the piperidine into the TE active site; the oxygen atom was positioned in the area of favorable polar interactions further stabilizing the water molecule that in the complex with **1** was hydrogen bonded to the hydroxyl side chain of Tyr1663.

### P^2^ SAR

In the original H2L evaluation of the P^2^ position, only replacement of the ethyl ester with a methyl amide was reported; this was to address a potential metabolic liability because of serum esterases.^17^ Analysis of the crystal structure highlighted the importance of the carbonyl oxygen in stabilizing the bioactive conformation and establishing a water-mediated hydrogen bond with His1664.^17^ It also showed that the group was largely solvent-exposed, and there were no specific interactions with the protein that needed to be maintained. Within a representative sample of known TB active agents, the average number of hydrogen bond donors (HBDs) has been estimated to be <4.^22^ At physiological pH, both **1** and **6** will have four HBDs. As P^2^ had no specific interactions with the protein, to reduce the overall number of HBDs, groups where the carbonyl oxygen was retained and the amide NH was removed were evaluated (Table 3). Given the improvements related to hERG ion channel inhibition seen in **6**, changes were made with the 2-oxa-6-azaspiro[3.4]octane at P^3^. Overall, a range of P^2^ groups were tolerated at this position, with respect to inhibition of Pks13 TE activity. Unfortunately, because of reduced microsomal stability and increased hERG channel inhibition, none of them represented an improvement over the methylamide (**23–27**). Replacing the methylamide with a methylketone **26** resulted in a significant increase in MIC potency, which was also reflected in the intramacrophage assay (Table 6). However, **26** had considerably worse microsomal stability and increased hERG channel inhibition. Despite its liabilities, **26** was tested in vivo to confirm that improvements in potency were mirrored in improved activity in the acute model. On dosing of **26** with 1-aminobenzotriazole (ABT) to block CYP450-associated metabolism, good exposure was achieved and the resultant drop in CFU was equivalent to that seen for the original lead **1**. Thus although **26** was not developable itself, the methylketone substituent at P^2^ generated such a potent MIC activity; thus it remained of interest for further exploration.

**Table 2:**
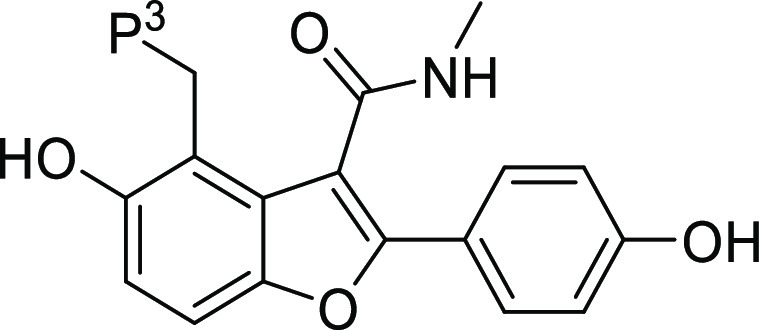

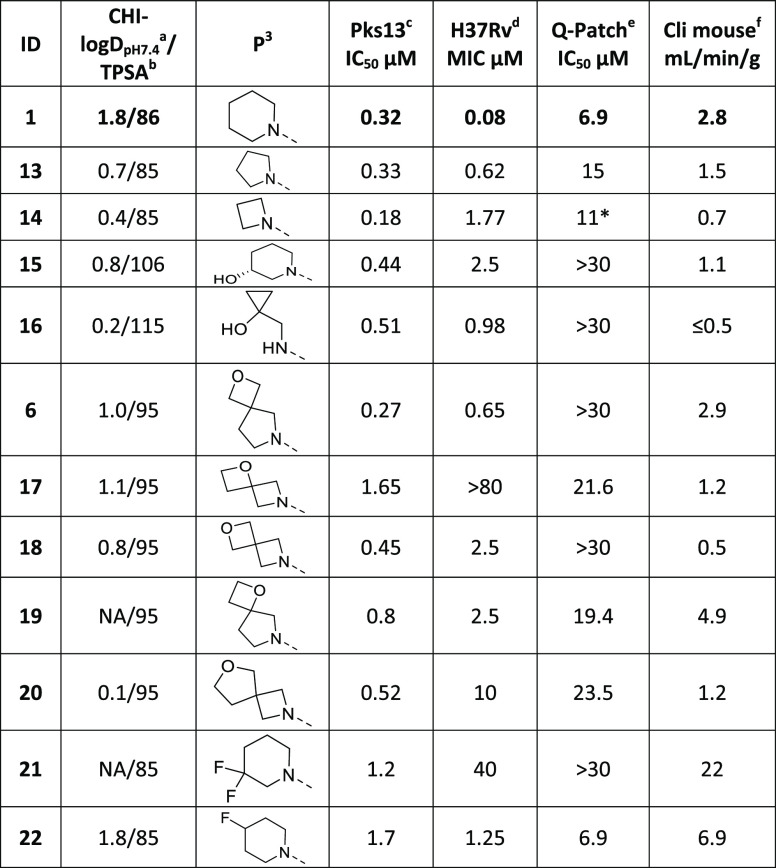
SAR at the P^3^ Position^a^^,^^b^^,^^c^^,^^d^^,^^e^^,^^f^

a CHI-Log*D*_pH7.4_ is a measure lipophilicity at pH 7.4.

b TPSA (Å^2^) is total polar surface area.

c *M. tuberculosis* Pks13 TE domain 50% inhibitory concentration as assessed using the reported methodology.^17^

d H32Rv MIC is the minimum concentration required to inhibit the growth of *M. tuberculosis* (H37Rv) in liquid culture.

e hERG functional Q-patch 50% inhibitory concentration. *hERG value was assessed in an alternative thallium flux assay.

f Intrinsic clearance (Cli) using CD1 mouse liver microsomes.

### P^1^ SAR

Although the original H2L evaluation showed that **1** did not appear to be a substrate for in vivo glucuronidation,^17^ the presence of two hydroxyl groups within the molecule was not considered a desirable drug-like feature. The hydroxyl at the 5 position of the benzofuran core makes an essential hydrogen bond with Asp1644 in the Pks13 binding pocket^17^ (Figure 1). In contrast, the phenol in P^1^ was primarily solvent-exposed, with a single point of interaction between the hydroxyl group and the Gln1633 side chain^17^ (Figure 1). Consequently, it was decided to explore modifications to the P^1^ phenol while retaining the reduction in CHI-log*D*_pH7.4_ achieved by the replacement of piperidine by 2-oxa-6-azaspiro[3.4]octane at P^3^. Efforts focused primarily on introducing bioisostere replacements (as exemplified by **32**), sterically hindering the phenol −OH by introducing F or Me substituents in the ortho position (**34** and **37**), changing the p*K*_a_ of the phenol by varying the electron distribution on the phenyl ring (**33, 35,** and **36**) or removing it (**28** and **31**). Table 4 summarizes the P^1^ modifications, simple changes to the hydroxyl group, including removal **28**, moving to the 3 position **29**, extension by the addition of a methyl linker **30** and capping to become a methoxy **31**, maintained good Pks13 activity but lost significant MIC activity (generally >10-fold). Replacing the hydroxyl HBD with an alternative bioisostere such as indole **32** again retained good Pks13 inhibition but showed a dramatic reduction in MIC activity. Replacing the phenol with a 5-hydroxypyridin-2-yl **33** resulted in an improvement in microsomal stability and retained low levels of hERG channel inhibition, although it did result in a reduction in both Pks13 and MIC potency. Addition of a single fluorine to the phenol had a minimal effect on the capacity to inhibit Pks13; while a 2-fluoro **35** retained good MIC activity, the introduction of a 3-fluoro **34** resulted in a reduced MIC. The incorporation of a 2,6-difluoro substitution **36** maintained good activity, and both Pks13 and MIC, as predicted, also gave rise to an improvement in microsomal stability. Introduction of a methyl group at the 3 position **37** had minimal impact on Pks13 inhibition and only a modest impact on MIC potency. However, it resulted in an increase in hERG channel inhibition and a modest decrease in microsomal stability. Conversely, a 2-methyl substituent **38** maintained its reduced hERG inhibition and metabolic stability but lost potency against both Pks13 and MIC. Overall, modifications to the phenol were generally well tolerated with respect to Pks13 activity, as was originally predicted because this region is solvent-exposed in the Pks13 crystal structure. Unexpectedly, these modifications often resulted in a loss of whole cell MIC activity despite retaining good Pks13 inhibition. Within the P^1^ analogues, the two most potent compounds **35** and **36,** which both maintained limited inhibition of the hERG ion channel and had good microsomal metabolic stability, were evaluated in vivo (Table 6). Neither compound had promising efficacy, **35** only achieved modest in vivo exposure which was reflected in a 1.4 log CFU reduction, whereas unfortunately **36** achieved very poor exposure and no in vivo activity was detected (Table 6).

**Table 3:**
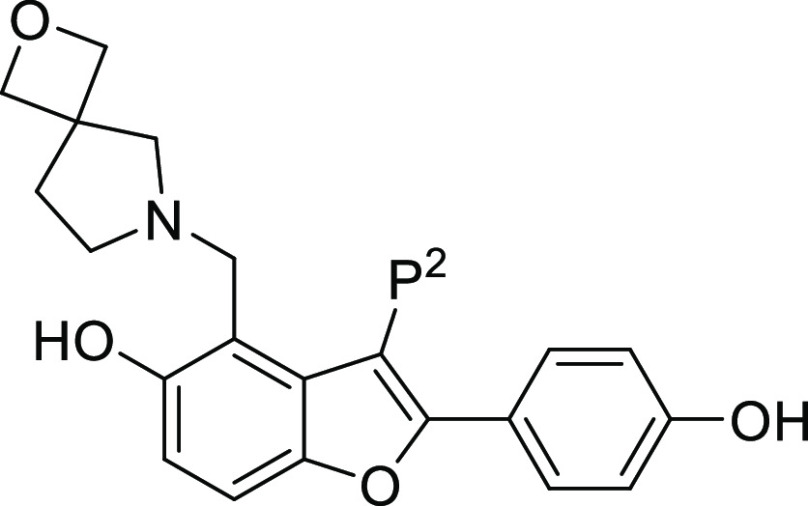

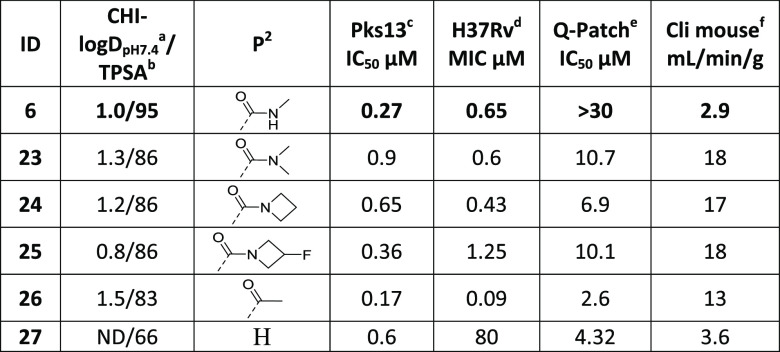
SAR at the P^2^ Position^a^

a See Table 2 for explanation.

**Table 4:**
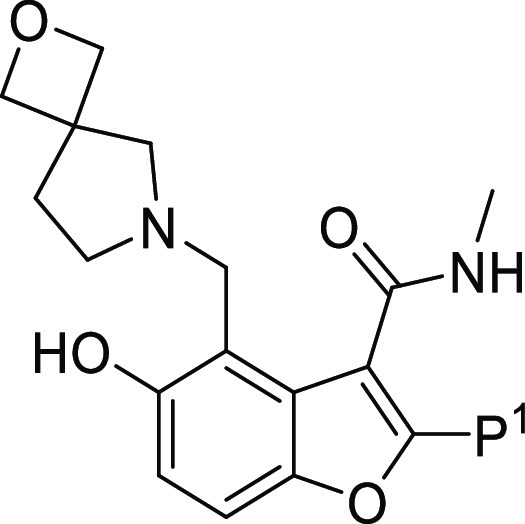

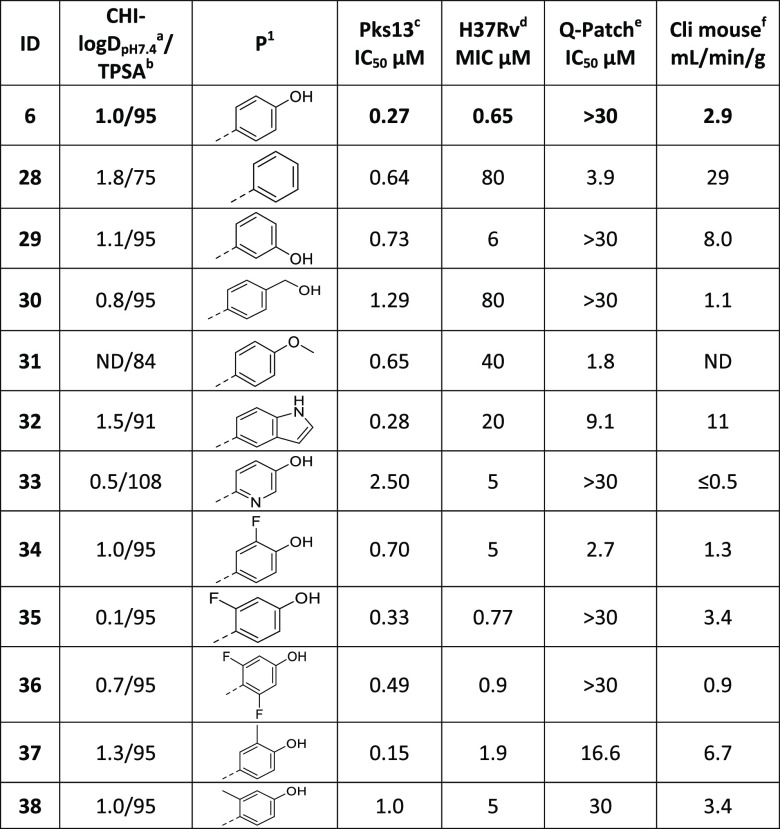
SAR at the P^1^ Position^a^

a See Table 2 for explanation.

### P^1^/P^2^/P^3^ Combinations

Based on the results of the modifications at individual positions around the molecule, combinations were explored evaluating different groups that had shown improvements in some of the key properties (Table 5). Focus was placed on preparing molecules with a clog*D*_pH7.4_ < 1.5 and TPSA < 110 as these were believed to be physiochemical criteria that were important for this series to retain MIC activity while reducing the hERG liability. Most of the compounds that met these criteria while retaining potency/microsomal stability contained the phenol or 5-hydroxypyridin-2-yl at P^1^ (**39–47** and **12**). 5-Hydroxypyridin-2-yl was particularly interesting because in some molecules it had reduced Pks13 activity with a concomitant reduction in MIC potency (**33** and **41**), while in other molecules, with a relatively minimal change, it looked promising (**43** and **12**). Once again, the most potent compounds which showed limited inhibition of the hERG ion channel and good microsomal metabolic stability **40, 43, 45,** and **12** were evaluated in vivo (Table 6). Three of the compounds (**40, 43,** and **45**) achieved poor in vivo exposure and correspondingly had no or minimal in vivo activity (Table 6). Compound **12**, while having modest PK exposure, retained good in vivo activity similar to that seen for **6** earlier (Table 6).

**Table 5:**
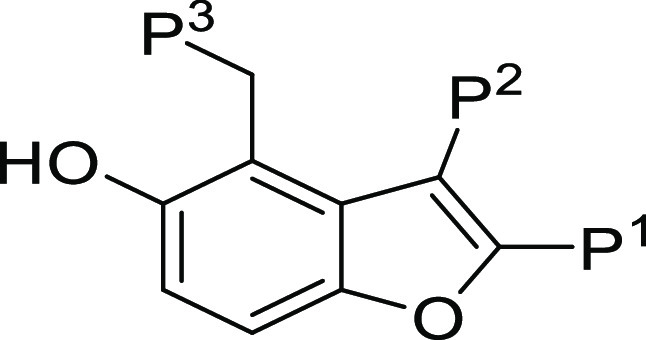

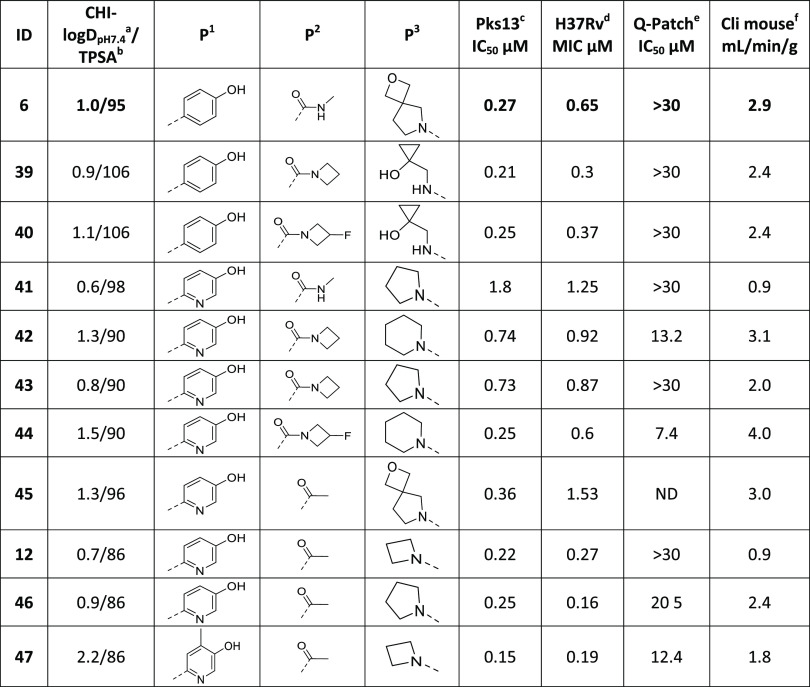
Combined SAR at the P^1^/P^2^/P^3^ Positions^a^

a See Table 2 for explanation.

### Confirmation of On-Target Mode of Action

**Figure 2:**
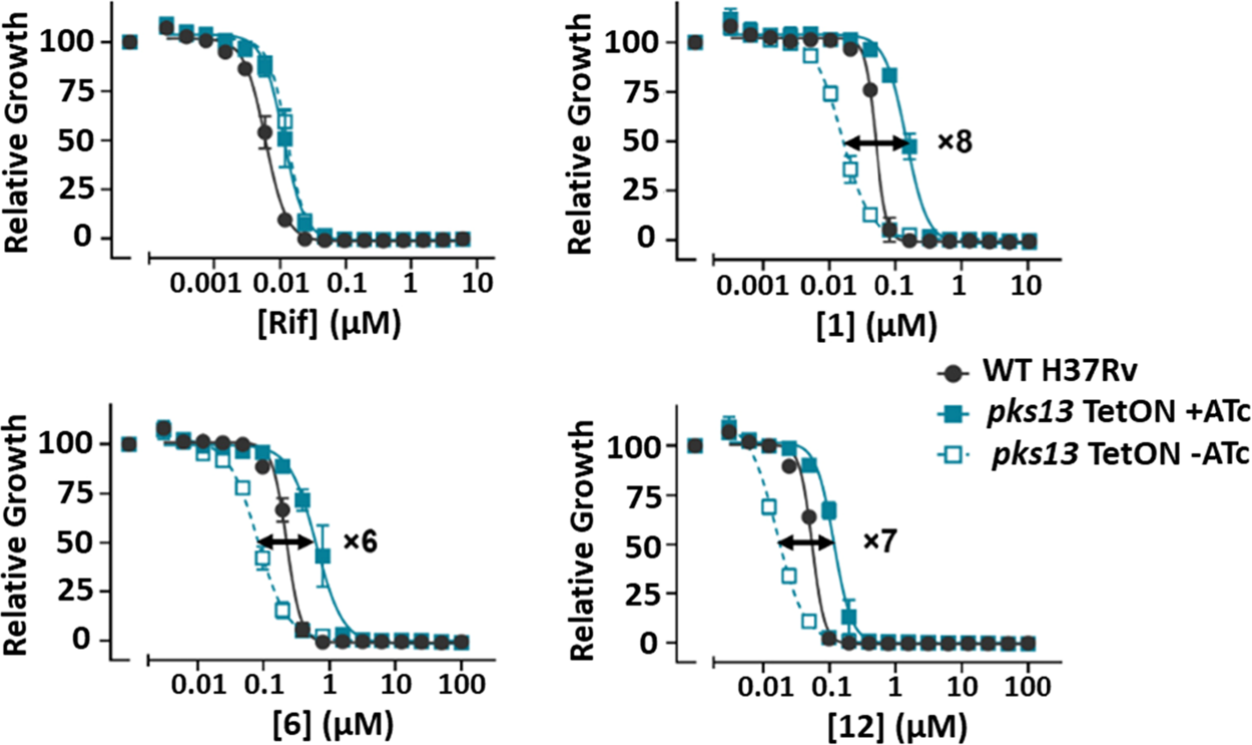
Dose response curves for compounds **1**, **6**, and **12** tested against H37Rv and a strain where *pks13* gene expression is regulated by the TetON promoter. Growth in the presence of anhydrotetracycline (ATc) results in modest transcriptional overexpression of *pks13* while removal of ATc results in transcriptional repression of *pks13* expression. Growth in the presence of negative control (rifampicin), **1**, **6**, and **12** are shown relative to DMSO-treated samples. Data are representative of two independent experiments.

To demonstrate the modified compounds remained on target, against Pks13, we used a strain in which *pks13* gene expression was transcriptionally regulated by the tetracycline repressor, TetR. In this strain (*pks13*-TetON), anhydrotetracycline (ATc at 500 ng/mL) induced expression of Pks13 to about four times that of wild-type (WT) levels, while in the absence of ATc, Pks13 was reduced to about one-fifth of the WT level. Thus, there was an approximately 20-fold shift in the amount of Pks13 in the regulated strain ±ATc with the WT level of Pks13 expression lying between these two extremes. The most promising molecules, **6** and **12**, were tested alongside the original lead **1** for their impact on the growth of the *pks13*-TetON ±ATc. Figure 2 shows that the three compounds had a clear shift in potency when the level of Pks13 expression was reduced by the absence of ATc compared to the same strain grown with ATc. All the compounds had a similar shift in potency, being six- to eightfold more sensitive to the compound when the strain expressed less Pks13. This was not due to any growth defect or phenotype caused by underexpression of Pks13, as shown by overlapping normalized growth of the strain ±ATc in the presence of rifampicin, which targets RNA synthesis, a pathway unrelated to Pks13 function. Thus, the molecules from the benzofuran series (**1, 6,** and **12**) are most likely to be killing *M. tuberculosis* by targeting Pks13.

### Assessment of In Vivo Activity

As discussed, compounds of interest from Tables 2–5 were selected for further evaluation. Initially, their activity was assessed against intracellular bacteria replicating inside macrophages (Table 6), under which conditions, all the compounds retained good activity against *M. tuberculosis*. The compounds were then tested in vivo in an acute murine model of TB infection as summarized in Table 6.

**Table 6:** Intramacrophage Activity and In Vivo Efficacy

ID	intramacrophage MIC IC_90_ (μM)^a^	dose mg/kg	Cmax (μg/mL)	AUC 24 h (μg/mL·h)	mean Log CFU reduction
1	<0.01	58	2.4	16.3	3.9^b^
13	0.08	70	0.6	3.9	2.8^b^
16	0.51	70	0.4	1.3	0.5
6	0.07	75^d^	3.0	15.7	3.1^b^
26^c^	<0.01	70	5.0	11.6	3.7^b^
35	0.17	70	1.8	5.8	1.4^b^
36	0.23	70	0.5	1.7	0.2
40	0.03	70	0.3	1.2	0.1
43	0.24	70	0.7	2.9	0
45	0.31	70	0.3	0.7	1^b^
12	0.03	75^d^	1.8	5.7	2.8^b^

a Intramacrophage MIC_90_ is the concentration required to inhibit 90% of the luminescent signal from a luciferase-expressing *M. tuberculosis* strain growing in THP1 monocytes.

b *p* < 0.05 ANOVA analysis.

c With 50 mg/kg ABT boosting.

d In general, initial acute efficacy was tested at a single dose with two animals per group, except where marked when for direct comparison the value shown was extracted from the nearest dose in the dose response curve (where 1 animal was used per dose).

**Figure 3:**
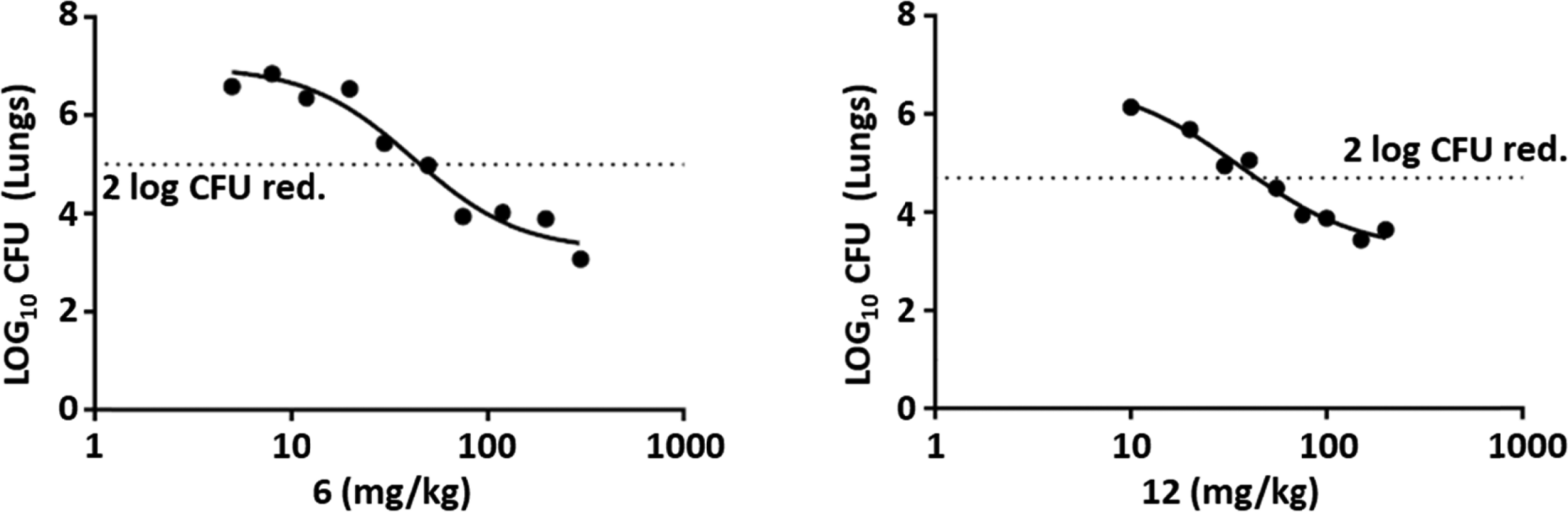
Dose response curves for compounds **6** and **12** in a murine model of acute *M. tuberculosis* H37Rv infection. Female C57BL/6 mice were intratracheally infected with ∼10^5^ CFU/mouse. Compounds were administered over a range of doses, once a day on day 1 to day 8 postinfection. On day 9, lungs were harvested, 24 h after the last drug administration. In these experiments, one animal was treated at each dose. The dotted line represented a 2 log_10_ reduction in CFU compared to the untreated control.

As highlighted during the SAR analysis, compounds **6** and **12** had the most promising in vivo activity, treatments with both agents resulting in an ∼3 log_10_ reduction in CFU. Compared to **1**, both **6** and **12** had decreased CHI log*D*_pH7.4_ (1.8 vs 1.0 and 0.7, respectively) and reduced predicted p*K*_a_ (10.2 vs 9.2 and 9.7, respectively). Such improvements had been the primary focus of the synthetic program and were reflected in reduced hERG ion channel inhibition compared to **1**. Given the positive progression in the molecules’ overall drug-like properties, **6** and **12** were evaluated in dose response efficacy studies (Figure 3). Both compounds showed good in vivo efficacy with a maximum effect of 3.8 log_10_ CFU reduction **6** and 3.6 log_10_ CFU reduction **12**. From the plots, the calculated 2 log_10_ reduction in CFU (ED_99_) was 46 and 49 mg/kg with an estimated AUC at this value of 9110 and 4666 ng/mL·h, respectively. Thus, while both compounds seemed roughly equipotent, the exposure required to achieve this potency was lower for **12**.

### Treatment of Chronic TB Infection in Mice

**Figure 4:**
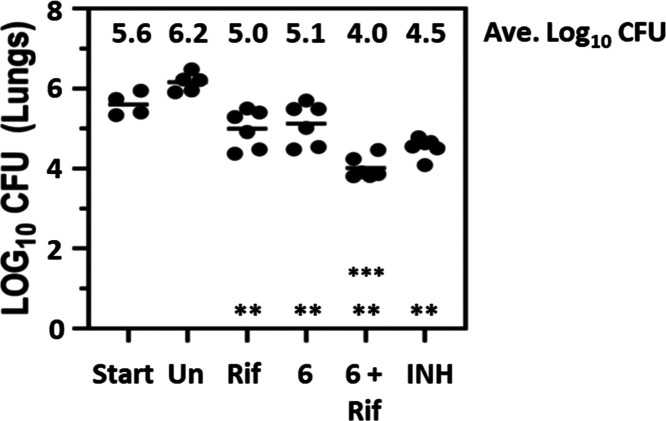
Activity of **6** in a murine chronic model of *M. tuberculosis* infection as a single agent and when combined with rifampicin. Female Balb/c mice were infected by aerosol with ∼150 CFU/mouse. Compound **6** (500 mg/kg) was dosed orally as either a single agent or in combination with rifampicin (10 mg/kg) once per day, 5 of 7 days per week, for 4 weeks from day 28 to day 56 postaerosol infection. Isoniazid (25 mg/kg) was used as a positive control for this experiment. Lungs were harvested 3 days following the last drug administration. Six mice were used for each treatment group. ** all single agents had statistically lower CFU than the untreated control (*p* < 0.005); *** combination of **6** and Rif had statistically lower CFU than the individual single agents (*p* < 0.005). Statistical analysis was by pairwise multiple comparison procedures (Tukey test).

The initial lead for the series **1** had been shown to act synergistically with rifampicin in murine models of chronic TB infection.^17^ As such, it was important to demonstrate that the compounds with improved hERG selectivity still retained this synergistic character. Compound **6** was studied in a chronic infection model (Figure 4), and it was dosed for 4 weeks (5 days out of 7) starting 4 weeks postinfection. At the end of the treatment period, lungs were harvested, and the extent of bacterial load was assessed by determination of the number of CFUs. In the lungs, both single agents had a significant >1 log_10_ reduction in bacterial load compared to the untreated control (*p* < 0.005). The combination of Rif and **6** had a >2 log_10_ reduction compared to the untreated control and was a significant improvement over either single agent alone (*p* < 0.005). A similar improvement in bacterial load was also seen in the spleens from the same experiments (data not shown). The results mirror those seen previously for compound **1** and indicate the potential for combining agents that target Pks13 with rifampicin, the most significant component of current clinical combination therapy.

### Evaluation of Potential Cardiac Liabilities

As mentioned, **6** and **12** both had reduced hERG ion channel inhibition compared to **1**, which had been the primary focus for the synthetic program. During the in vivo evaluation, no tolerability issues were reported for either **6** or **12**, even when **6** was dosed at 500 mg/kg for 4 weeks during the chronic study. However, considering the initial concerns related to hERG channel inhibition, it was prudent to assess if any cardiovascular liability remained by performing an ex vivo perfused rabbit ventricular wedge (RVW) study (Table 7). The compounds were evaluated over a concentration range (3, 10, 30, and 100 μM); unexpectedly, both compounds showed a concentration-dependent prolongation of QT, QRS, and Tp-e intervals (data for 30 μM tests shown in Table 7). Both **6** and **12** had a torsades de pointes (TdP) score^23^ of 4 and 4.5, respectively, at 100 μM, with scores >2.5 indicating a risk of cardiovascular toxicity because of the development of TdP arrhythmias. At 30 μM, a TdP-like arrhythmia was observed for **12**, but none was reported for **6**.

**Table 7:** RVW Data for Compounds **6** and **12**^a^

	% change			
compounds (30 μM)	QRS	QT	Tp-e	TdP score
**6**	62	28	20	1.5
**12**	43	12	128	4

a Different cardiac peak intervals were monitored in an ex vivo RVW assay (stimulation frequency 0.5 Hz) at four different compound concentrations (only data for 30 μM shown) as described previously.^24^

To further explore these unexpected cardiotoxicity findings, **6** (as the compound with a better profile in the RVW experiment) was sent for analysis in a Cardiacprofiler panel looking at the impact on eight different cardiac ion channels (Eurofins). The compound was assessed at 30 μM, and the % inhibition was determined for each of the ion channels (Table 8). As can be seen, none of the ion channels showed greater than 50% inhibition at 30 μM, and it was the hERG channel that had the greatest % inhibition, at 25%.

**Table 8:** Inhibition of Cardiac Ion Channels by Compound **6** at 30 μM^a^

	Nav1.5	Kv4.3/ KChIP2	Cav1.2	hKv1.5	KCNQ1/minK	hERG	HCN4	Kir2.1
**6**	19%	15%	–1%	–1%	1%	25%	–2%	11%

a Compound **6** was profiled in a Cardiacprofiler panel (Eurofins); each data point equates to an average from *n* = 7 replicates.

## Conclusions

Given the urgent need for new agents to treat TB infections, there was significant interest in the identification of a benzofuran series that inhibited Pks13, a novel target for TB drugs.^17^ Although the initial lead molecule **1** had very good in vivo efficacy, it had a clear hERG liability which suggested potential for cardiotoxicity. The liability was hypothesized to be related to the lipophilic amine^21^ present in the piperidine moiety that was involved in making direct interactions between the molecule and the enzyme active site. The focus of this report describes the lead optimization approach taken to try and reduce this hERG liability. Compound **6** had the best all-round properties of the series, but as its in vivo efficacious dose was likely to be high, a cardiotoxicity study was performed to ensure that the hERG liability had been eliminated. Unexpectedly, the ex vivo study highlighted a significant cardiovascular risk for **6,** despite the reduction in its hERG channel inhibition. The Cardiacprofiler panel indicated that even allowing for the improvements over **1**, the hERG channel inhibition of **6** was still the most likely liability. These results support the ongoing debate about whether it is more appropriate to reduce hERG inhibition below even IC_20_ values, although the complexity of measuring such low levels of inhibition on a sigmoid curve can introduce variabilities/inaccuracies.^25,26^ The current report has shown that multiple molecules from this series can demonstrate excellent in vivo activity against *M. tuberculosis*, thereby confirming that the TE domain of Pks13 is an attractive target for identifying potential novel TB drugs. In addition, it also demonstrates that agents which inhibit Pks13 also synergize well with rifampicin in vivo, the main stay of current combination therapies. Unfortunately, within this benzofuran series there is a clear association of the lipophilic amine for both direct target binding and off-target cardiovascular toxicity associated with hERG channel inhibition. This is important to highlight to the field as the original TAM16 molecule was considered an exciting early lead being referenced in TB development pipelines (https://www.newtbdrugs.org/pipeline/discovery) and in new agent literature.^27,28^ Furthermore, other groups have recently reported two related series as promising early start points, that are also dependent on a similar basic group interaction between molecule and Pks13 and thus have the same potential liability.^29,30^ To fully exploit the Pks13 TE domain as a promising target, it will require: more extensive evaluation of changes to the piperidine in P^3^ to reduce further the lipophilicity/basicity; a more significantly in-depth exploration, replacing the piperidine/benzofuran core with an alternative core, which was beyond the scope of this lead optimization project; or the initiation of a screening campaign to identify alternative chemical start points.

## Experimental Section

### General Chemistry Methods

Chemicals and solvents were purchased from commercial vendors and were used as received, unless otherwise stated. Dry solvents were purchased in Sure Seal bottles stored over molecular sieves. Unless otherwise stated, herein reactions have not been optimized. Analytical thin-layer chromatography (TLC) was performed on precoated TLC plates (Kieselgel 60 F254, BDH). Developed plates were air-dried and analyzed under a UV lamp (UV 254/365 nm), and/or KMnO_4_ was used for visualization. Flash chromatography was performed using Combiflash Companion Rf (Teledyne ISCO), and prepacked silica gel columns were purchased from Grace Davison Discovery Science or SiliCycle. Mass-directed preparative high-performance liquid chromatography (HPLC) separations were performed using a Waters HPLC (2545 binary gradient pumps, 515 HPLC make-up pump, 2767 sample manager) connected to a Waters 2998 photodiode array and a Waters 3100 mass detector. Preparative HPLC separations were performed with a Gilson HPLC (321 pumps, 819 injection module, and 215 liquid handler/injector) connected to a Gilson 155 UV/vis detector. On both instruments, HPLC chromatographic separations were conducted using Waters XBridge C18 columns, 19 mm × 100 mm, 5 μm particle size, using 0.1% ammonia in water (solvent A) and acetonitrile (solvent B) as the mobile phase.^1^H NMR spectra were recorded on a Bruker Advance II 500 or 400 spectrometer operating at 500 and 400 MHz (unless otherwise stated) using CDCl_3_, DMSO-*d*_6_, or CD_3_OD solutions. Chemical shifts (δ) are expressed in ppm recorded using the residual solvent as the internal reference in all cases. Signal splitting patterns are described as singlet (s), doublet (d), triplet (t), multiplet (m), broadened (br), or a combination thereof. Coupling constants (*J*) are quoted to the nearest 0.1 Hertz (Hz). Low-resolution electrospray (ES) mass spectra were recorded on a Bruker Daltonics MicrOTOF mass spectrometer run in positive mode. High-resolution mass spectroscopy (HRMS) was performed using a Bruker Daltonics MicrOTOF mass spectrometer. Liquid chromatography–mass spectrometry (LC–MS) analysis and chromatographic separation were conducted with either a Bruker Daltonics MicrOTOF mass spectrometer connected to an Agilent diode array detector or a Thermo Dionex Ultimate 3000 RSLC system with a diode array detector, the column used was a Waters XBridge column (50 mm × 2.1 mm, 3.5 μm particle size), and the compounds were eluted with a gradient of 5–95% acetonitrile/water +0.1% ammonia, or with an Agilent Technologies 1200 series HPLC connected to an Agilent Technologies 6130 quadrupole LC/ MS, connected to an Agilent diode array detector, the column used was a Waters XBridge column (50 mm × 2.1 mm, 3.5 μm particle size) or a Waters X-select column ( 30 mm × 2.1 mm, 2.5 μm particle size) with a gradient of 5–90% acetonitrile/water +0.1% formic acid, or with an Advion Expression Mass Spectrometer connected to a Thermo Dionex Ultimate 3000 HPLC with diode array detector, the column used was a Waters XBridge column (50 mm × 2.1 mm, 3.5 μm particle size) or a Waters X-select column ( 30 mm × 2.1 mm, 2.5 μm particle size) with a gradient of 5–90% acetonitrile/water +0.1% formic acid. All final compounds showed a chemical purity of ≥95% as determined by the UV chromatogram (190–450 nm) obtained by LC–MS analysis. Microwave-assisted chemistry was performed using a CEM or a Biotage microwave synthesizer.

#### 5-hydroxy-2-(4-methoxyphenyl)benzofuran-3-carboxylic Acid (3)

Ethyl 5-hydroxy-2-(4-methoxyphenyl)benzofuran-3-carboxylate (0.50 g, 1.60 mmol) was dissolved in EtOH (4 mL) and water (4 mL), and sodium hydroxide (0.32 g, 8.00 mmol) was added. The reaction mixture was heated to 80 °C overnight. After cooling, the reaction mixture was taken to acidic pH by 2 M HCl addition, extracted with EtOH (×3), and evaporated to give the title compound (440 mg, 1.47 mmol, 91%). LCMS: *m*/*z* 285 [M + H]^+^. ^1^H NMR (400 MHz, DMSO) δ 12.93 (br s, 1H), 9.40 (s, 1H), 8.02 (d, *J* = 9.0 Hz, 2H), 7.50 (d, *J* = 8.7 Hz, 1H), 7.44 (d, *J* = 2.5 Hz, 1H), 7.13 (d, *J* = 9.0 Hz, 2H), 6.85 (dd, *J* = 2.6, 8.8 Hz, 1H), 3.90 (s, 3H).

#### 5-hydroxy-2-(4-methoxyphenyl)-*N*-methylbenzofuran-3-carboxamide (4)

A mixture of 5-hydroxy-2-(4-methoxyphenyl)benzofuran-3-carboxylic acid (58.00 g, 204.04 mmol), methanamine hydrochloride (41.33 g, 612.12 mmol), and EDCI (78.23 g, 408.08 mmol) in pyridine (600.00 mL) was stirred at 15 °C for 12 h. The reaction solution was concentrated under reduced pressure to give a crude product (80 g). The residue was purified by silica chromatography (petroleum ether/ethyl acetate = 20/1, 0/1) to give the title compound (30.00 g, 96.87 mmol, 47%) as a yellow solid. LCMS: *m*/*z* 298 [M + H]^+^. ^1^H NMR (400 MHz, DMSO) δ 9.3 (br s, 1H), 8.30 (d, *J* = 4.4 Hz, 1H), 7.82 (d, *J* = 8.5 Hz, 2H), 7.42 (d, *J* = 8.8 Hz, 1H), 7.07 (d, *J* = 8.4 Hz, 2H), 6.93 (s, 1H), 6.77 (d, *J* = 8.7 Hz, 1H), 3.83 (s, 3H), 2.81 (d, *J* = 4.5 Hz, 3H).

#### 5-hydroxy-2-(4-hydroxyphenyl)-*N*-methylbenzofuran-3-carboxamide (5)

To a solution of 5-hydroxy-2-(4-methoxyphenyl)-*N*-methylbenzofuran-3-carboxamide (27 g, 90.82 mmol) in DCM (270 mL) was added BBr_3_ (68.25 g, 272.46 mmol) at 0 °C under N_2_. The mixture was stirred at 25 °C for 1 h. The reaction solution was quenched with aqueous NaHCO_3_ (1 M) until pH 7 at 0 °C. The reaction mixture was filtered, and the filtered cake was washed with water (15 mL) and dried under vacuum to give the title compound with an impurity (20 g, 67.78 mmol, 75%). LCMS: *m*/*z* 284 [M + H^+^]. ^**1**^H NMR (400 MHz, MeOD) δ 9.93 (s, 1H), 9.27 (s, 1H), 8.23 (d, *J* = 4.5 Hz, 1H), 7.70 (d, *J* = 8.7 Hz, 2H), 7.45–7.31(m, 1H), 6.97–6.83 (m, 3H), 6.79–6.68 (m, 1H), 6.53 (s, 1H, impurity), 2.80 (d, *J* = 4.5 Hz, 3H).

#### 4-((2-oxa-6-azaspiro[3.4]octan-6-yl)methyl)-5-hydroxy-2-(4-hydroxyphenyl)-*N*-methylbenzofuran-3-carboxamide (6)

To a solution of 5-hydroxy-2-(4-hydroxyphenyl)-*N*-methyl-benzofuran-3-carboxamide (400 mg, 1.41 mmol), 2-oxa-6-azaspiro[3.4]octane oxalate (315 mg, 1.55 mmol) and DIPEA (237 mg, 1.83 mmol) in EtOH (15 mL) and water (3 mL) was added formaldehyde 37% solution in water (137 mg, 1.69 mmol), and the mixture was stirred at 80 °C overnight. Saturated aqueous NaHCO_3_ was added, and the mixture was extracted with EtOAc and evaporated to afford a residue. The residue was purified by preparative HPLC (acidic, 5–50% ACN). Further purification was carried out by HPLC (basic, 5–40%) to give the title compound (118 mg, 0.27 mmol, 19%). LCMS: *m*/*z* 409 [M + H]^+^. ^1^H NMR (400 MHz, DMSO) δ 9.9 (br s, 1H), 8.53–8.50 (m, 1H), 7.56 (d, *J* = 8.9 Hz, 2H), 7.33 (d, *J* = 8.7 Hz, 1H), 6.87 (d, *J* = 8.8 Hz, 2H), 6.73 (d, *J* = 8.8 Hz, 1H), 4.50–4.44 (m, 4H), 3.83 (s, 2H), 2.82–2.74 (m, 5H), 2.54 (dd, *J* = 7.3, 7.3 Hz, 2H), 2.08 (dd, *J* = 7.0, 7.0 Hz, 2H). ^13^C NMR (125 MHz, DMSO) δ 166.2, 158.8, 153.8, 152.3, 147.2, 128.0, 126.9, 120.9, 116.2, 113.9, 113.8, 112.7, 110.5, 82.4, 63.7, 53.0, 52.2, 45.1, 35.9, 26.6. HRMS (ESI): *m*/*z* calcd for C_23_H_25_N_2_O_5_ [M + H^+^]: 409.1763. Found 409.1773.

#### 5-Hydroxy-2-(4-hydroxyphenyl)-*N*-methyl-4-(pyrrolidin-1-ylmethyl)benzofuran-3-carboxamide Formate (13)

To a solution of 5-hydroxy-2-(4-hydroxyphenyl)-*N*-methyl-benzofuran-3-carboxamide (400 mg, 1.41 mmol) in THF (6 mL), were added pyrrolidine (110 mg, 1.55 mmol), formaldehyde 37% solution in water (114 mg, 1.41 mmol), and acetic acid (0.05 mL). The mixture was sealed under nitrogen and reacted in the microwave at 80 °C for 20 min. EtOAc (10 mL) and saturated aqueous NaHCO_3_ (10 mL) were added. The aqueous phase was extracted with ethyl acetate (2 × 5 mL), dried, and concentrated to afford the crude product (357 mg), which was purified by a preparative HPLC reverse phase column using gradient eluent 5–9% MeCN in water (using 0.1% formic acid as the modifier) to give an off-white solid (165 mg). The solid was suspended in 2-butanol (8 mL), and the mixture was heated with swirling for 10 min. The mixture was allowed to stand for 18 h, and then the solid was filtered, dried, and freeze-dried from MeCN-H_2_O (1:1, 3 mL) to give the title compound as an off-white powder (76 mg, 0.18 mmol, 12%). LCMS *m*/*z* 367 [M + H]^+^ .^1^H NMR (500 MHz, MeOD) δ 8.53 (s, 1H), 7.59–7.56 (m, 2H), 7.46 (d, *J* = 8.9 Hz, 1H), 6.93–6.88 (m, 3H), 4.44 (s, 2H), 3.33–3.31 (m, 4H), 2.89 (s, 3H), 2.09 (t, *J* = 6.6 Hz, 4H). HRMS (ESI): *m*/*z* calcd for C_21_H_23_N_2_O_4_ [M + H^+^]: 367.1658. Found 367.1677.

#### 4-(Azetidin-1-ylmethyl)-5-hydroxy-2-(4-hydroxyphenyl)-*N*-methyl-benzofuran-3-carboxamide (14)

To a stirred solution of 5-hydroxy-2-(4-hydroxyphenyl)-*N*-methyl-benzofuran-3-carboxamide (60 mg, 0.21 mmol) in THF (3 mL) were added azetidine (13 mg, 0.23 mmol), formaldehyde 37% solution in water (17 mg, 0.21 mmol), and acetic acid (0.05 mL), and the mixture was stirred at 75 °C in the microwave for 15 min. Saturated aqueous NaHCO_3_ was added, and the mixture was extracted with EtOAc and evaporated to afford a residue. The residue was purified by preparative HPLC (basic, 20–95%) to give the title compound (8 mg, 0.02 mmol, 10%). LCMS: *m*/*z* 353 [M + H]^+^. ^1^H NMR (500 MHz, MeOD) δ 7.60 (d, *J* = 8.7 Hz, 2H), 7.27 (d, *J* = 8.8 Hz, 1H), 6.86 (d, *J* = 8.7 Hz, 2H), 6.74 (d, *J* = 8.8 Hz, 1H), 3.98 (s, 2H), 3.43 (t, *J* = 7.3 Hz, 4H), 2.94 (s, 3H), 2.18 (m, 2H).

#### (*R*)-5-hydroxy-2-(4-hydroxyphenyl)-4-((3-hydroxypiperidin-1-yl)methyl)-*N*-methylbenzofuran-3-carboxamide Formate (15)

To a solution of 5-hydroxy-2-(4-hydroxyphenyl)-*N*-methylbenzofuran-3-carboxamide (100 mg, 353 μmol) in dioxane (800 μL) and H_2_O (800 μL) was added formaldehyde 37% solution in water (28 mg, 353 μmol, 26 μL) and (*3R*)-piperidin-3-ol hydrochloride (35 mg, 261 μmol). The mixture was stirred at 50 °C for 3 h. The mixture was concentrated under vacuum. The residue was extracted with ethyl acetate (2 mL × 2). The combined organic phase was dried with anhydrous Na_2_SO_4_, filtered, and concentrated in vacuum. The crude product was purified by prep-HPLC (column: Phenomenex Synergi C18 150 × 25 × 10 μm; mobile phase: [water (0.225% formic acid)-ACN]; B%: 1–32%, 10 min) to give the title compound (68 mg, 154 μmol, 44%) as a white solid. LCMS: *m*/*z* 397 [M + H]^+^. ^1^H NMR (500 MHz, DMSO) δ 8.53 (q, *J* = 4.4 Hz, 1H), 8.19 (s, 1H), 7.60–7.56 (m, 2H), 7.35–7.33 (m, 1H), 6.89–6.86 (m, 2H), 6.74–6.71 (m, 1H), 3.86–3.66 (m, 2H), 3.58–3.48 (m, 1H), 2.82–2.75 (m, 4H), 2.63 (d, *J* = 9.9 Hz, 1H), 2.13–1.97 (m, 2H), 1.77–1.67 (m, 2H), 1.47–1.37 (m, 1H), 1.25–1.19 (m, 1H). HRMS (ESI): *m*/*z* calcd for C_22_H_25_N_2_O_5_ [M + H^+^]: 397.1763. Found 397.1988.

#### a5-hydroxy-4-((((1-hydroxycyclopropyl)methyl)amino)methyl)-2-(4-hydroxyphenyl)-*N*-methylbenzofuran-3-carboxamide (16)

A mixture of 5-hydroxy-2-(4-hydroxyphenyl)-*N*-methylbenzofuran-3-carboxamide (70 mg, 0.25 mmol), 1-(aminomethyl)cyclopropanol (23 mg, 0.27 mmol), DIPEA (32 mg, 0.27 mmol), and formaldehyde 37% solution in water (22 mg, 0.27 mmol) in ethanol (2 mL) and water (0.5 mL) was heated at 80 °C for 12 h. Water was then added, and the reaction mixture was extracted with DCM (×2). The combined DCM extracts were dried via a hydrophobic filter and concentrated under reduced pressure. The product was purified via prep. HPLC 5–50% variable gradient using formic acid method to give a solid. The product was further purified via SCX ion exchange chromatography to give the title compound (15 mg, 0.04 mmol, 16%). LCMS: *m*/*z* 383 [M + H]^+ 1^H NMR (500 MHz, MeOD) δ 7.60 (d, *J* = 8.7 Hz, 2H), 7.28 (d, *J* = 8.9 Hz, 1H), 6.86 (d, *J* = 8.7 Hz, 2H), 6.77 (d, *J* = 8.7 Hz, 1H), 4.17 (s, 2H), 2.91 (s, 3H), 2.77 (s, 2H), 0.75–0.71 (m, 2H), 0.58–0.54 (m, 2H). HRMS (ESI): *m*/*z* calcd for C_21_H_23_N_2_O_5_ [M + H^+^]: 383.1607 Found 383.1589.

#### 4-((1-oxa-6-azaspiro[3.3]heptan-6-yl)methyl)-5-hydroxy-2-(4-hydroxyphenyl)-*N*-methylbenzofuran-3-carboxamide Formate (17)

5-hydroxy-2-(4-hydroxyphenyl)-*N*-methylbenzofuran-3-carboxamide (112 mg, 0.39 mmol), 1-oxa-6-azaspiro[3.3]heptane; oxalic acid (89 mg, 0.47 mmol), formaldehyde 37% solution in water (51 mg, 0.63 mmol), and DIPEA (112 mg, 0.86 mmol) were dissolved in ethanol (3.5 mL), and the reaction mixture was heated at 80 °C overnight. The reaction mixture was reduced *in vacuo* and purified by HPLC (1–50%) MeCN in H_2_O (formic acid modifier) to give an impure compound, and the material was repurified on the HPLC (1–40%) MeCN in H_2_O (formic acid modifier) to give the title compound (6 mg, 0.01 mmol, 3%) as an off-white solid. LCMS: *m*/*z* 395 [M + H]^+^. ^1^H NMR (400 MHz, DMSO) δ 9.93 (br s, 1H), 8.59 (q, *J* = 4.8 Hz 1H,), 8.17 (s, 1H), 7.59–7.56 (m, 2H), 7.34 (d, *J* = 8.8 Hz), 1H, 6.89–6.86 (m, 2H), 6.73 (d, *J* = 8.8 Hz, 1H) , 4.37 (t, *J* = 7.5 Hz, 2H), 3.82 (s, 2H), 3.57 (d, *J* = 7.8 Hz, 2H), 3.19 (d, *J* = 7.8 Hz, 2H), 2.79 (d, *J* = 4.8, 3H), 2.77 (t, *J* = 7.5 Hz, 2H).

#### 4-((2-oxa-6-azaspiro[3.3]heptan-6-yl)methyl)-5-hydroxy-2-(4-hydroxyphenyl)-*N*-methylbenzofuran-3-carboxamide Formate (18)

To a solution of 5-hydroxy-2-(4-hydroxyphenyl)-*N*-methyl-benzofuran-3-carboxamide (300 mg, 1.05 mmol), 2-oxa-6-azaspiro[3.3]heptane, oxalic acid (220 mg, 1.16 mmol) and DIPEA (177 mg, 1.37 mmol) in ethanol (10 mL), and water (2 mL) was added formaldehyde 37% solution in water (94 mg, 1.16 mmol), and the mixture was refluxed overnight. Saturated aqueous NaHCO_3_ was added, and the mixture was extracted with EtOAc, dried, and evaporated. The residue was purified by silica chromatography (5% MeOH in DCM) and then prep-HPLC (acidic, 5–40% ACN) to give the title compound (155 mg, 0.33 mmol, 31%). LCMS *m*/*z* 395 [M + H]^+^. ^1^H NMR (400 MHz, DMSO) δ 8.56 (q, *J* = 4.5 Hz, 1H), 8.16 (s, 1H), 7.59–7.56 (m, 2H), 7.34 (d, *J* = 8.8 Hz, 1H), 6.89–6.86 (m, 2H), 6.74 (d, *J* = 8.8 Hz, 1H), 4.61 (s, 4H), 3.80 (s, 2H), 3.41 (s, 4H), 2.79 (d, *J* = 4.6 Hz, 3H). HRMS (ESI): *m*/*z* calcd for C_22_H_23_N_2_O_5_ [M + H^+^]: 395.1607. Found 395.1620.

#### 4-((1-oxa-6-azaspiro[3.4]octan-6-yl)methyl)-5-hydroxy-2-(4-hydroxyphenyl)-*N*-methylbenzofuran-3-carboxamide (19)

5-hydroxy-2-(4-hydroxyphenyl)-*N*-methyl-benzofuran-3-carboxamide (150 mg, 0.52 mmol), formaldehyde 37% solution in water (64 mg, 0.79 mmol), DIPEA (102 mg, 0.79 mmol), and 1-oxa-7-azaspiro[3.4]octane; oxalic acid (107 mg, 0.52 mmol) were added to ethanol (3 mL) and heated under microwave conditions at 120 °C for 1 h. Water was added with DCM, and organics were separated and dried via a hydrophobic filter and evaporated. The residue was purified via silica column chromatography (DCM-MeOH 10% gradient elution) to give a gum. The gum was further purified by trituration in MeOH to give the title compound as a beige solid. (20 mg, 0.04 mmol, 9%). LCMS: *m*/*z* 409 [M + H^+^]. ^1^H NMR (500 MHz, MeOD) δ 7.60–7.56 (m, 2H), 7.27–7.24 (m, 1H), 6.85–6.82 (m, 2H), 6.74–6.71 (m, 1H), 4.45–4.37 (m, 2H) 3.95–3.92 (m, 2H), 3.13–3.08 (m, 1H), 2.87 (s, 3H), 2.85–2.79 (m, 1H), 2.75–2.66 (m, 3H), 2.57–2.52 (m, 1H), 2.30–2.23 (m, 1H), 2.20–2.13 (m, 1H).

#### 4-((6-oxa-2-azaspiro[3.4]octan-2-yl)methyl)-5-hydroxy-2-(4-hydroxyphenyl)-*N*-methylbenzofuran-3-carboxamide (20)

To a solution of 5-hydroxy-2-(4-hydroxyphenyl)-*N*-methyl-benzofuran-3-carboxamide (40 mg, 0.14 mmol) and the 6-oxa-2-azaspiro[3.4]octane (17 mg, 0.15 mmol) in ethanol (1 mL) and water (0.2 mL) was added DIPEA (27 mg, 0.21 mmol) and formaldehyde 37% solution in water (14 mg, 0.18 mmol), and the mixture was refluxed overnight. Saturated aqueous NaHCO_3_ was added, and the mixture was extracted with EtOAc, dried, and concentrated. The residue was purified by silica column chromatography (5% MeOH in DCM). The residue was then purified by prep-HPLC (acidic, 5–90% ACN) to give the title compound (6 mg, 0.014 mmol, 9%). LCMS: *m*/*z* 409 [M + H^+^]. ^1^H NMR (400 MHz, DMSO) δ 8.63 (d, *J* = 4.6 Hz, 1H), 7.60–7.56 (m, 2H), 7.40–7.37 (m, 1H), 6.90–6.87 (m, 2H), 6.80–6.77 (m, 1H), 3.96–3.95 (m, 2H), 3.73 (s, 2H), 3.67–3.63 (m, 2H), 3.45–3.35 (m, 4H), 2.82 (d, *J* = 4.6 Hz, 3H), 2.06 (t, *J* = 6.9 Hz, 2H).

#### Ethyl 2-(5-(benzyloxy)pyridin-2-yl)-5-methoxybenzofuran-3-carboxylate (8)

A mixture of (3-(ethoxycarbonyl)-5-methoxybenzofuran-2-yl)boronic acid (2.00 g, 7.57 mmol), 5-(benzyloxy)-2-bromopyridine (2.00 g, 7.57 mmol), potassium carbonate (2.09 g, 15.15 mmol), and Pd(dppf)Cl_2_ (309 mg, 0.38 mmol) in 1,4-dioxane (30 mL) and water (7 mL) was degassed and purged with N_2_, and the reaction mixture was stirred at 80 °C overnight. Water was added, and the aqueous layer was extracted with EtOAc. The combined organic extracts were washed with brine, evaporated, and purified by silica column chromatography (20–30% EtOAc/heptanes) to afford the title compound (1.35 g, 3.02 mmol, 40%): LCMS: m/z 404 [M + H]^+^. ^1^H NMR (400 MHz, CDCl_3_) δ 8.58 (d, *J* = 2.8 Hz, 1H), 8.22 (d, *J* = 8.8 Hz, 1H), 7.55–7.36 (m, 8H), 7.00 (dd, *J* = 2.7, 9.0 Hz, 1H), 5.22 (s, 2H), 4.44 (q, *J* = 7.1 Hz, 2H), 3.92 (s, 3H), 1.44 (t, *J* = 7.2 Hz, 3H).

#### 2-(5-(benzyloxy)pyridin-2-yl)-5-methoxybenzofuran-3-carboxylic Acid (9)

To a mixture of sodium hydroxide (545 mg, 13.63 mmol) in ethanol (6 mL) was added a solution of ethyl 2-(5-(benzyloxy)pyridin-2-yl)-5-methoxybenzofuran-3-carboxylate (1.10 g, 2.73 mmol) in water (6 mL), and the reaction mixture was stirred at 80 °C overnight. The reaction mixture was cooled and acidified with HCl (2 M), extracted with EtOAc, and evaporated to give the title compound (985 mg, 2.36 mmol, 86%). LCMS: *m*/*z* 376 [M + H]^+^. ^1^H NMR (400 MHz, DMSO) δ 16.99 (s, 1H), 8.69 (d, *J* = 2.8 Hz, 1H), 8.26 (d, *J* = 8.9 Hz, 1H), 7.94 (dd, *J* = 2.9, 9.0 Hz, 1H), 7.77 (d, *J* = 2.8 Hz, 1H), 7.65 (d, *J* = 9.0 Hz, 1H), 7.52 (d, *J* = 7.0 Hz, 2H), 7.47–7.39 (m, 3H), 7.08 (dd, *J* = 2.7, 9.0 Hz, 1H), 5.36 (s, 2H), 3.84 (s, 3H).

#### 1-(2-(5-(benzyloxy)pyridin-2-yl)-5-methoxybenzofuran-3-yl)ethan-1-one (10)

To a mixture of 2-(5-(benzyloxy)pyridin-2-yl)-5-methoxybenzofuran-3-carboxylic acid (400 mg, 1.07 mmol) and *N*-methoxymethanamine hydrochloride (156 mg, 1.60 mmol) in DMF (10 mL) were added HATU (446 mg, 1.17 mmol) and DIPEA (413 mg, 3.20 mmol), and the reaction mixture was stirred at room temperature overnight. Water was added, and the aqueous layer was extracted with EtOAc, washed with brine, evaporated, and purified by silica column chromatography (50% EtOAc/heptanes) to give 2-(5-(benzyloxy)pyridin-2-yl)-N,5-dimethoxy-*N*-methylbenzofuran-3-carboxamide (360 mg, 0.82 mmol, 77%). To a solution of 2-(5-(benzyloxy)pyridin-2-yl)-N,5-dimethoxy-*N*-methylbenzofuran-3-carboxamide (280 mg, 0.67 mmol) in THF (7 mL) at −78 °C under N_2_ was added methyllithium (29 mg, 1.34 mmol, 1.6 M solution), and the reaction mixture was stirred at −78 °C for 30 min. Water (3 mL) and HCl (3 mL, 2 M) were added, and the mixture was warmed to room temperature and stirred at room temperature for 30 min. Saturated NaHCO_3_ was added, and the aqueous layer was extracted with EtOAc, evaporated, and purified by silica column chromatography (20–30% EtOAc/heptanes) to afford the title compound (195 mg, 0.50 mmol, 74%). LCMS: *m*/*z* 374 [M + H]^+^. ^1^H NMR (400 MHz, CDCl_3_) δ 8.53 (d, *J* = 2.8 Hz, 1H), 7.90 (d, *J* = 8.8 Hz, 1H), 7.53–7.48 (m, 3H), 7.46–7.39 (m, 5H), 6.98 (dd, *J* = 2.7, 8.9 Hz, 1H), 5.23 (s, 2H), 3.91 (s, 3H), 2.59 (s, 3H).

#### 1-(5-hydroxy-2-(5-hydroxypyridin-2-yl)benzofuran-3-yl)ethan-1-one (11)

To a solution of 1-(2-(5-(benzyloxy)pyridin-2-yl)-5-methoxybenzofuran-3-yl)ethan-1-one (190 mg, 0.51 mmol) in DCM (5 mL) at −78 °C under N_2_ was added tribromoborane (637 mg, 2.5 mmol), and the reaction mixture was warmed to room temperature and stirred at room temperature for 3 h. Saturated NaHCO_3_ was added, and the layers were separated. The aqueous layer was extracted with EtOAc, evaporated, and purified by silica column chromatography (50–100% EtOAc/heptanes) to give the title compound (120 mg, 0.42 mmol, 83%). LCMS: *m*/*z* 270 [M + H]^+^. ^1^H NMR (400 MHz, MeOD) δ 8.29 (d, *J* = 2.8 Hz, 1H), 7.86 (d, *J* = 8.6 Hz, 1H), 7.41–7.37 (m, 3H), 6.88 (dd, *J* = 2.7, 8.8 Hz, 1H), 2.46 (s, 3H).

#### 1-(4-(azetidin-1-ylmethyl)-5-hydroxy-2-(5-hydroxypyridin-2-yl)benzofuran-3-yl)ethan-1-one Formate (12)

To a solution of 1-(5-hydroxy-2-(5-hydroxypyridin-2-yl)benzofuran-3-yl)ethan-1-one (50 mg, 0.19 mmol) in THF (1 mL) and water (0.2 mL) were added azetidine (12 mg, 0.20 mmol) and formaldehyde 37% solution in water (20 mg, 0.24 mmol), and the reaction mixture was stirred at 70 °C for 3 h. Saturated NaHCO_3_ was added, and the aqueous layer was extracted with EtOAc, evaporated, and purified by HPLC (5–95% MeCN/water, acidic method) to give the title compound (12 mg, 0.03 mmol, 16%). LCMS: *m*/*z* 339 [M + H]^+^. ^1^H NMR (400 MHz, DMSO) δ 8.21 (d, *J* = 2.8 Hz, 1H), 8.17 (s, 1H), 7.78 (d, *J* = 8.6 Hz, 1H), 7.36–7.31 (m, 2H), 6.84 (d, *J* = 8.8 Hz, 1H), 3.65 (s, 2H), 3.11 (t, *J* = 7.1 Hz, 4H), 2.61 (s, 3H), 1.98–1.91 (m, 2H). ^13^C NMR (125 MHz, DMSO) δ 199.6, 163.9, 154.5, 152.8, 151.8, 147.8, 139.5, 138.6, 126.5, 123.4, 122.3, 119.6, 114.8, 114.7, 110.7, 54.1, 53.4, 32.4, 17.3. HRMS (ESI): *m*/*z* calcd for C_19_H_19_N_2_O_4_ [M + H^+^]: 339.1339. Found 339.1348.

### Pks13 Enzyme Assays and Crystallography

A previous report describes the methods used in this study to assay Pk13 enzyme inhibition, soaking of Pks13 TE domain crystals with compounds, data acquisition, and interpretation.^17^ Atomic coordinates and structure factors for the reported crystal structure (Table S1) have been deposited with the Protein Data Bank under accession codes: PDB: 7M7V (Pks13-TE:6).

### *M. tuberculosis* H37Rv MIC Determination

All methods used for both extra and intracellular MIC determinations have been described previously.^31^

### MIC Comparison for H37Rv and *pks13*-TetON

WT H37Rv and *pks13*-TetON (which produces about four times the WT Pks13 level when grown with anhydrotetracycline [ATc] and one-fifth of WT Pks13 when grown without ATc [manuscript in preparation]) were each cultured in 10 mL of Middlebrook 7H9 (with Hygromycin at 50 μg/mL, Zeocin at 25 μg/mL, and ATc at 500 ng/mL for the mutant strain) supplemented with 0.2% (v/v) glycerol, 0.05% (v/v) Tyloxapol, and ADNaCl (0.5% [w/v] BSA, 0.2% [w/v] dextrose, and 0.85% [w/v] NaCl) in a 25 cm^2^ tissue culture flask with a vented cap. After approx. 7 d at 37 °C and 5% CO_2_ in a humidified incubator, growing to approx. midlog phase, each of the cultures was washed with fresh 7H9 and suspended to a final OD_580_ of 0.01 in 7H9 (+/–ATc at 500 ng/mL for the mutant). Compounds were solubilized in DMSO and dispensed into black, clear-bottom 384-well tissue culture plates using an HP D300e Digital Dispenser as 16-point, 2-fold dilution series in triplicate. OD_580_ 0.01 suspension (50 μL) was pipetted to each well, and cultures were incubated for 7–14 days at 37 °C under the same conditions as above. Final OD_580_ values were normalized to no-drug (1% [v/v] DMSO) control wells.

### HepG2 Cytotoxicity

Compound dilution curves were plated directly using a Labcyte Echo 550 acoustic dispenser (125 nL) in 384-well white clear-bottomed plates (Greiner). HepG2 cells (ECACC 85011430) were cultured in Minimum Essential Medium (supplemented with glutamax) with 10% FCS and plated (25 μL) using a WellMate dispenser (1 × 10^5^ per well) and incubated for 72 h. Doxorubicin was used as a positive control drug. Resazurin was then added to each well at a final concentration of 45 μM, and fluorescence was measured using PHERAstar LS (BMG Labtech) after 4 h of further incubation (excitation of 528 nm and emission of 590 nm). Raw data were normalized to controls and expressed as % growth. The IC_50_ was defined as the compound concentration that resulted in 50% inhibition.

### Intrinsic Clearance (Cli) Experiments

Mouse microsomal stability studies were performed exactly as reported.^32^

### CHI Log*D*_pH7.4_ Measurement

Test compounds were prepared as 0.5 mM solutions in 50:50 acetonitrile/water and analyzed by reversed-phase HPLC-UV (wavelength 254 nm) using a Phenomenex Luna C18 100 Å 150 × 4.6 mm 5 micron column with a gradient of aqueous phase (50 mM ammonium acetate (pH 7.4)) and mobile phase (acetonitrile) as described.^33,34^

### Calculation of p*K*_a_

The protonation state of each designed compound at pH 7.4 was calculated using MoKa from the Molecular Discovery suite.^20^

### Murine Models of TB Infection

All studies were conducted either in accordance with the European Directive 2010/63/EEC and the GSK Policy on the Care, Welfare and Treatment of Laboratory Animals or were reviewed by the Institutional Animal Care and Use Committee at the institution where the work was performed. Both single-point and dose response acute studies were performed as described.^35^ In short, pathogen-free 8- to 10-week-old female C57BL/6 mice (Harlan Labs) were intratracheally infected with approximately 100,000 CFU/mouse (*M. tuberculosis* H37Rv). Agents were administered by oral gavage in 1% methylcellulose day 1 to day 8 postinfection. Lungs were harvested on day 9 after infection, 24 h after the last administration. Blood samples were obtained at different time points from infected mice to measure the levels of the tested compounds. The chronic model including combinations with rifampicin has been described previously.^17^ Briefly, 6- to 8-week-old female specific-pathogen-free immunocompetent BALB/c mice (Charles River, Wilmington, MA) were infected via a low-dose aerosol exposure to *M. tuberculosis* Erdman (150 CFU/mouse). Following infection, the mice were randomly divided into treatment groups (6 mice/group). At day 28 postaerosol, four mice were euthanized as the start of treatment controls. All drugs were administered 5 days per week (Monday to Friday) for 4 weeks. Negative control mice remained untreated. The final sacrifice occurred 3 days following the last compound dose. Compound **6** (500 mg/kg) was administered in 1% carboxymethylcellulose. In cases where animals received two drugs, RIF was administered first, and then compound **6** was administered 1 h later.

### Cardiovascular Assays

Routine in vitro Q-patch assays were performed as previously described.^36,37^ More involved follow-up analysis using an ex vivo RVW assay was as described.^24^

### Molecular Modeling—Molecular Docking

For each designed compound, a low-energy conformer was generated using LigPrep in the Schrödinger platform. Compounds were docked into the published Pks13 binding site for **1** (PDB 5V3Y) using Glide, part of the Schrödinger suite of programs.^38,39^ A 20 Å cubic box centered on the **1** ligand was used to generate the docking grid. All the protein hydroxyl groups in the binding site area were allowed to rotate to optimize hydrogen bonding interactions. The crystallographic water molecules were removed, and the default settings were used for the docking. Up to 10 docking poses were generated and visually inspected.

### Molecular Modeling—Binding Site Analysis

Energetically favorable interaction hotspots were determined using FLAP by Molecular Discovery.^40^ In particular, the probe N1 was used to assess areas where a ligand HBD motif could be positioned, the probe O for HBDs, N1+ for positively ionizable groups, and DRY and C1 were used as probes for hydrophobic interactions. Crystallographic water molecules were assessed as for their structural, displaceable, or bulk character using the WaterFLAP module.

## Supporting Information Available

Table S1. X-ray data collection and refinement statistics for Pks13 TE–Compound **6** complex, synthetic Schemes S1–S20 and chemistry experimental section for molecules **21–47**, and representative in vivo compound HPLC-UV traces (PDF)
Manuscript key in vitro data table with PAINS alert analysis and SMILES (CSV)

## Notes

PDB code for Pks13 with bound TAM16 (**1**) is 5V3Y and with Compound **6** is 7M7V.

## Abbreviations

ABT: aminobenzotriazole
AcOH: acetic acid
ACN: MeCN, acetonitrile
ANOVA: analysis of variance
ATc: anhydrotetracycline
CFU: colony-forming units
Cli: intrinsic clearance
DIPEA: N,N-diisopropylethylamine
EDCI: 1-(3-dimethylaminopropyl)-3-ethylcarbodiimide hydrochloride
EtOAc: ethyl acetate
EtOH: ethanol
HATU: 1-[bis(dimethylamino)methylene]-1H-1,2,3-triazolo[4,5-*b*]pyridinium 3-oxide hexafluorophosphate
HOBt: hydroxybenzotriazole
H2L: hit to lead
INH: isoniazid
InMac: intermacrophage
LO: lead optimization
Log*D*: logarithm of distribution coefficient
MeLi: methyllithium
MeOH: methanol
MeOD: deuterated methanol
NIS: N-iodosuccinimide
PE: petroleum ether
p*K*_a_: logarithm of acid dissociation constant
Pks13: polyketide synthase 13
rif: rifampicin
RVW: rabbit ventricular wedge
TE: thioesterase
TdP: torsades de pointes
TPSA: total polar surface area.
