# Supplementary material for: Optimization of TAM16, a Benzofuran That Inhibits the Thioesterase Activity of Pks13; Evaluation toward a Preclinical Candidate for a Novel Antituberculosis Clinical Target
Source: J Med Chem. 2021 Dec 15;65(1):409–23. doi: 10.1021/acs.jmedchem.1c01586 (PMC8762665; doi:10.1021/acs.jmedchem.1c01586)

Supporting information for:

Optimisation of TAM16, a benzofuran that inhibits the thioesterase activity of Pks13; evaluation towards a preclinical candidate for a novel anti-tuberculosis clinical target.

Caroline Wilson[‡ƛαμν], Peter Ray[‡ƛμν], Fabio Zuccotto[‡ƛμν], Jorge Hernandez[‡α], Anup Aggarwal[ƚπμ], Claire Mackenzie[‡α], Nicola Caldwell[‡α], Malcolm Taylor[‡α], Margaret Huggett[‡α], Michael Mathieson[‡α], Dinakaran Murugesan[‡α], Alasdair Smith[‡α], Susan Davis[‡α], Mattia Cocco[‡α], Maloy K. Parai[ƚαμ], Arjun Acharya[ƚα], Fabio Tamaki[‡π], Paul Scullion[‡ö], Ola Epemolu[‡öμ], Jennifer Riley[‡ö], Laste Stojanovski[‡ö], Eva Maria Lopez-Román[ɣπ], Pedro Alfonso Torres-Gómez[ɣπ], Ana Maria Toledo[ɣπ], Laura Guijarro-Lopez[ɣπ], Isabel Camino[ɣö], Curtis A. Engelhart[¥πβ], Dirk Schnappinger[¥πβ], Lisa M. Massoudi[ɋπ], Anne Lenaerts[ɋπ], Gregory T. Robertson[ɋπβ], Chris Walpole[≠μβ], David Matthews[≠μ], David Floyd[≠μ], James C. Sacchettini[ƚμβ], Kevin D. Read[‡öβ], Lourdes Encinas[ɣμ], Robert H Bates[ɣμβ], Simon R Green[‡πμνβ*] and Paul G Wyatt[‡μβ*]

[‡]Drug Discovery Unit, Division of Biological Chemistry and Drug Discovery, College of Life Sciences, University of Dundee, Dundee, DD1 5EH, UK
[ƚ]Department of Biochemistry and Biophysics, Texas A&M University, College Station, Texas 77843, USA
[ɣ]Global Health Pharma R&D, GlaxoSmithKline, Severo Ochoa 2, Tres Cantos, 28760, Madrid, Spain
[¥]Department of Microbiology and Immunology, Weill Cornell Medical College, New York, NY, 10065, USA
[ɋ]Mycobacteria Research Laboratories, Department of Microbiology, Immunology, and Pathology, Colorado State University, 200 W. Lake Street, Fort Collins, Colorado 80523-1682, USA
[≠]Structural Genomics Consortium, Research Institute of the McGill University Health Centre, 1001 Boulevard Décarie, Site Glen Block E, ES1.1614, Montréal, QC H4A 3J1, Canada

[ƛ]contributed equally to medicinal chemistry design and planning, [α]molecule design and synthesis, [π]planned/executed biological/crystallographic studies, [ö]planned/executed DMPK/safety studies, [μ]contributed to monthly project planning meetings, [ν]wrote draft manuscript, [β]edited manuscript and *corresponding authors.

***Corresponding Authors**

Simon R Green (s.r.green@dundee.ac.uk)
Paul G Wyatt (p.g.wyatt@dundee.ac.uk)

Supporting Information contents:

Table S1. X-ray data collection and refinement statistics for Pks13 TE – Compound 6 complex
Synthetic schemes S1-S20 and chemistry experimental for molecules 21-47
Representative *in vivo* compound HPLC UV traces
Manuscript key in *vitro* data table with PAINS alert analysis and SMILES (separate csv file)

Table S1. X-ray data collection and refinement statistics for Pks13 TE – Compound 6 complex

	**Pks13 TE – Compound 6 complex**
**PDB ID**	**7M7V**
**Data collection**	
Space group	*P 21 21 2*
Unit cell dimensions	
a, *b*, *c* (Å)	89.97, 110.28, 57.63
α, β, γ (°)	90, 90, 90
Resolution range (Å)	48.53 - 2.29 (2.37 - 2.29)[a]
Total reflections	161516 (16353)
Unique reflections	26061 (2545)
R_{merge} (%)	15.1 (173.8)
$I / \sigma I$	9.6 (1.7)
Completeness (%)	97.7 (98.2)
Redundancy	6.2 (6.4)
$CC_{1/2}$ (%)	99.4 (44.3)
**Refinement**	
Reflections used in refinement	25910 (2545)
R_{work} (%)	22.3 (30.6)
R_{free} (%)	26.9 (36.2)
Number of atoms	
Protein	4035
Ligands	60
Water	96
Average B-factor (Å^2)	51.2
Protein	51.2
Ligand/ion	57.8
Water	46.2
RMS deviations	
Bond lengths (Å)	0.010
Bond angles (°)	0.97
Ramachandran Plot	
Favored (%)	98.11
Allowed (%)	1.89
Outliers (%)	0

[a]Statistics for the highest-resolution shell are shown in parentheses. Data for the structure was collected from a single crystal.

Chemistry Experimental Section

Scheme S1

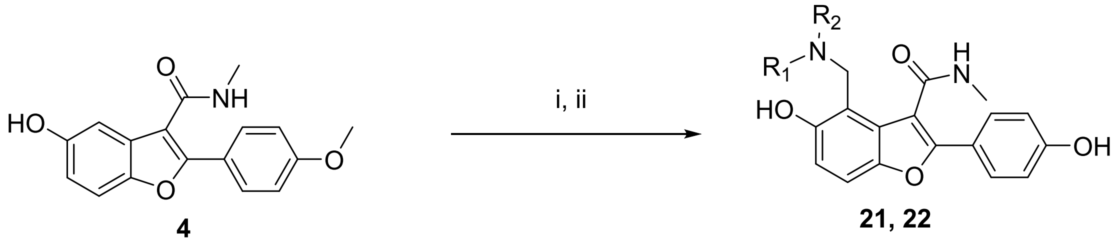

[a]*Reagents and conditions:* (i) Formaldehyde, amine, dioxane, water, 80 °C; (ii) BBr_3, DCM, 0 °C to 25 °C or pyridine hydrochloride, 180 °C.

4-((3,3-difluoropiperidin-1-yl)methyl)-5-hydroxy-2-(4-hydroxyphenyl)-N-methylbenzofuran-3-carboxamide (21)

To a solution of 5-hydroxy-2-(4-methoxyphenyl)-N-methylbenzofuran-3-carboxamide (100 mg, 336 µmol) in dioxane (1.6 mL) and H_2O (1.6 mL) was added formaldehyde 37% solution in water (32 mg, 403 µmol) and 3,3-difluoropiperidine (40 mg, 336 µmol). The mixture was stirred at 80 °C for 15 h. The mixture was concentrated under reduced pressure, extracted with ethyl acetate and evaporated to give a residue. The residue was purified by preparative HPLC (0.225% Formic Acid-ACN) to give impure intermediate. (30 mg, 69 µmol, 21%). The reaction was repeated and batches combined. To a solution of 4-((3,3-difluoropiperidin-1-yl)methyl)-5-hydroxy-2-(4-methoxyphenyl)-N-methylbenzofuran-3-carboxamide (60 mg, 139 µmol) in DCM (1 mL) was added BBr_3 (104 mg, 418 µmol) in one portion at 0 °C under N_2. The mixture was stirred at 25 °C for 15 h. The reaction solution was quenched by aqueous $NaHCO_3$ (1 M) till pH =7 at 0 °C. The reaction solution was purified by preparative HPLC (0.225% Formic Acid-ACN) to afford the title compound (30 mg, 71 µmol, 52%). LCMS: m/z 417 $[M+H^+]$. ^1H NMR (400 MHz, DMSO) δ 10.09 (br s, 2H), 8.51 (d, *J*=4.6 Hz, 1H), 7.55 7.59 (m, 2H), 7.39 - 7.35 (m, 1H), 6.90 - 6.86 (m, 2H), 6.79 - 6.76 (m, 1H), 3.83 (s, 2H), 2.79 - 2.73 (m, 5H), 2.50-2.45 (m, 2H), 1.96 - 1.65 (m, 4H).

4-((4-fluoropiperidin-1-yl)methyl)-5-hydroxy-2-(4-hydroxyphenyl)-N-methylbenzofuran-3-carboxamide formate (22)

To a solution of 5-hydroxy-2-(4-methoxyphenyl)-N-methylbenzofuran-3-carboxamide (200 mg, 672 µmol) in dioxane (1.6 mL) and H_2O (1.6 mL) was added formaldehyde 37% solution in water (65 mg, 807 µmol) and 4-fluoropiperidine (112 mg, 807 µmol). The mixture was stirred at 80 °C overnight, concentrated under vacuum, extracted with ethyl acetate and evaporated to afford a residue. The residue was purified by preparative HPLC (0.1%TFA-ACN) to give impure intermediate (20 mg, 48 µmol, 7 %). Reaction repeated and batches combined. A solution of 4-((4-fluoropiperidin-1-yl)methyl)-5-hydroxy-2-(4-methoxyphenyl)-N-methyl benzofuran-3-carboxamide (50 mg, 121 µmol) and pyridine hydrochloride (3.22 g, 27.88 mmol) was stirred at 180°C for 2 h. The mixture was evaporated to give a residue. The residue was purified by preparative HPLC (water (0.225%Formic Acid)-ACN) to give the title compound (25 mg, 61.59 µmol, 51%). LCMS: m/z 399 $[M+H^+]$. ^1H NMR (500 MHz, DMSO) δ 9.92 (br s, 1H), 8.52 - 8.47 (m, 1H), 7.59 - 7.56 (m, 2H), 7.36 - 7.33 (m, 1H), 6.90 - 6.87 (m, 2H), 6.76 - 6.74 (m, 1H), 4.82 - 4.66 (m, 1H), 3.76 (s, 2H), 2.79 - 2.77 (m, 3H), 2.62-2.53 (m, 2H), 2.47-2.38 (m, 2H), 1.93 - 1.81 (m, 2H), 1.77 - 1.72 (m, 2H).

Scheme S2

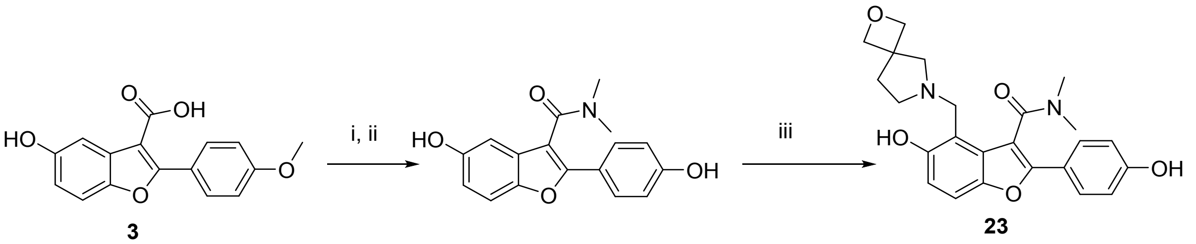

[a]*Reagents and conditions:* (i) HATU, DIPEA, N-methylmethanamine, rt; (ii) BBr_3, DCM, -78 °C to rt; (iii) formaldehyde, 2-oxa-7-azaspiro[3.4]octane; oxalic acid, DIPEA, EtOH, H_20, 80 °C.

5-hydroxy-2-(4-hydroxyphenyl)-N,N-dimethylbenzofuran-3-carboxamide

To a suspension of 5-hydroxy-2-(4-methoxyphenyl)benzofuran-3-carboxylic acid (250 mg, 0.87 mmol) in THF (5mL) were successively added HATU (367 mg, 0.96 mmol) , DIPEA (227 mg, 1.75 mmol) and N-methylmethanamine (47 mg , 1.05 mmol) and the mixture was stirred for 3 h at rt. Water was added and the mixture was extracted with EtOAc (x3), washed with brine, dried and concentrated. The residues were purified by column chromatography (50% EtOAc in heptane) to give impure 5-hydroxy-2-(4-methoxyphenyl)-N,N-dimethyl-benzofuran-3-carboxamide (270 mg, 0.73 mmol 83%). To a solution of 5-hydroxy-2-(4-methoxyphenyl)-N,N-dimethyl-benzofuran-3-carboxamide (250 mg, 0.80 mmol) in DCM (5 mL) at under N_2 was added tribromoborane (442 mg, 1.76 mmol) at -78°C and the mixture was stirred at rt for 2 h. Aqueous sat $NaHCO_3$ was added, phases were separated and the aqueous phase was extracted with EtOAc (x3), dried and concentrated. The residue was purified by silica column chromatography (50-70% EtOAc in heptane) to give the title compound (74 mg, 0.23 mmol, 26%). LCMS: m/z 298 $[M+H]^+$. ^1H NMR (400 MHz, DMSO) δ 9.99 - 9.99 (m, 1H), 9.30 (s, 1H), 7.57 - 7.54 (m, 2H), 7.43 - 7.40 (m, 1H), 6.90 - 6.87 (m, 2H), 6.78 - 6.72 (m, 2H), 3.09 (s, 3H), 2.81 (s, 3H).

4-((2-oxa-6-azaspiro[3.4]octan-6-yl)methyl)-5-hydroxy-2-(4-hydroxyphenyl)-N,N-dimethylbenzofuran-3-carboxamide formate (23)

To a solution of 5-hydroxy-2-(4-hydroxyphenyl)-N,N-dimethyl-benzofuran-3-carboxamide (30 mg, 0.10 mmol) 2-oxa-7-azaspiro[3.4]octane; oxalic acid (22mg, 0.11 mmol) and DIPEA (16 mg, 0.13 mmol) in ethanol (1 mL) and water (0.2 mL) was added formaldehyde 37% solution in water (9 mg, 0.12 mmol) and the mixture was stirred at 80°C overnight. Saturated aqueous $NaHCO_3$ was added and the mixture was extracted with EtOAc dried and concentrated to afford a residue. Purification by prep HPLC (acidic, 5-95% ACN) to give the title compound (12 mg, 0.02 mmol 24%). LCMS: m/z 423 $[M+H]^+$. ^1H NMR (400 MHz, DMSO) δ 8.16 (s, 1H), 7.51 - 7.48 (m, 2H), 7.37 - 7.34 (m, 1H), 6.90 - 6.87 (m, 2H), 6.80 - 6.77 (m, 1H), 4.49 - 4.40 (m, 4H), 3.82 (d, *J*=13.0 Hz, 1H), 3.65 (d, *J*=13.0 Hz, 1H), 3.05 (s, 3H), 2.70 - 2.69 (m, 5H), 2.63 - 2.54 (m, 1H), 2.46-2.37 (m, 1H), 2.10 - 2.05 (m, 2H).

Scheme S3

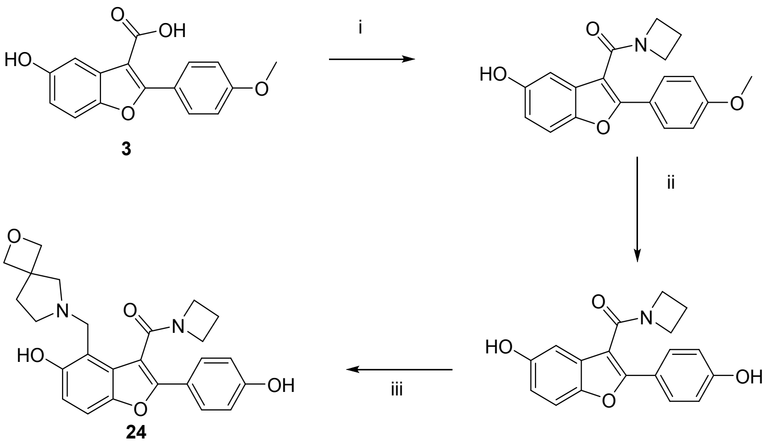

[a]Reagents and conditions: (i) Azetidine, HATU, DIPEA, rt; (ii) BBr_3, DCM, 0 °C- to 25 °C; (iii) formaldehyde, 2-oxa-7-azaspiro[3.4]octane; oxalic acid, DIPEA, EtOH, H_20, 60 °C.

azetidin-1-yl(5-hydroxy-2-(4-methoxyphenyl)benzofuran-3-yl)methanone

To a suspension of 5-hydroxy-2-(4-methoxyphenyl)benzofuran-3-carboxylic acid (100 mg, 0.35 mmol) and azetidine (24 mg, 0.42 mmol) in DMF (2 mL) were successively added HATU (147 mg, 0.38 mmol) and DIPEA (136 mg, 1.05mmol) and the mixture was stirred at rt overnight. Water was added and the mixture was extracted with EtOAc (x3), washed with brine, dried and concentrated. The residue was purified by silica column chromatography (30-50% EtOAc in heptane) to give impure title compound (98 mg, 0.255 mmol, 72%). LCMS: m/z 324 $[M+H]^+$. ^{1}H NMR (400 MHz, CDCl3) δ 8.62 (s, 1H), 7.81 - 7.78 (m, 2H), 7.46 (d, *J*=2.4 Hz, 1H), 7.38 - 7.35 (m, 1H), 7.06 - 7.03 (m, 2H), 6.91 (dd, *J*=2.6, 8.8 Hz, 1H), 4.30 - 4.24 (m, 2H), 3.92 (s, 3H), 3.69 - 3.63 (m, 2H), 2.20 - 2.11 (m, 2H).

azetidin-1-yl(5-hydroxy-2-(4-hydroxyphenyl)benzofuran-3-yl)methanone

To a solution of azetidin-1-yl(5-hydroxy-2-(4-methoxyphenyl)benzofuran-3-yl)methanone (5.00 g, 15.46 mmol) in DCM (50 mL) was added BBr_3 (11.62 g, 46.38 mmol) at 0 °C under N_2.The mixture was stirred at 25 °C for 15 h. The reaction solution was quenched by EtOH at 0 °C. The reaction mixture was filtered and the filtered cake was washed with water, dried under vacuum to give the title compound (3.00 g, 9.70 mmol, 63%) as a brown solid. ^{1}H NMR (400MHz, DMSO) δ 10.04 (s, 1H), 9.32 (s, 1H), 7.71 - 7.62 (m, 2H), 7.40 (d, *J*=8.8 Hz, 1H), 6.91 (d, *J*=8.8 Hz,3H), 6.75 (dd, *J*=2.5, 8.8 Hz, 1H), 4.07 (t, J=7.7 Hz, 2H), 3.70 - 3.60 (m, 2H), 2.14 (q, *J*=7.7 Hz, 2H).

(4-(2-oxa-6-azaspiro[3.4]octan-6-ylmethyl)-5-hydroxy-2-(4-hydroxyphenyl)benzofuran-3-yl)(azetidin-1-yl)methanone formate (24)

A solution of azetidin-1-yl(5-hydroxy-2-(4-hydroxyphenyl)benzofuran-3-yl)methanone (500 mg, 1.62 mmol), 2-oxa-7-azaspiro[3.4]octane.oxalic acid (362 mg, 1.78 mmol) and formaldehyde 37% solution in water (144 mg, 1.78 mmol) in EtOH (10 mL) and H_2O (2 mL) was stirred at 60 °C for 40 h. The reaction solution was concentrated under vacuum. The reaction was purified by prep-HPLC (water (0.225% formic acid)-ACN; 6%-36%) to give the title compound (300 mg, 0.62 mmol, 37%). LCMS: Rt m/z 435 $[M+H^+]$. ^{1}H NMR (400MHz, DMSO) δ 8.22 (s, 1H), 7.57 (d, *J*=8.7 Hz, 2H), 7.33 (d, *J*=8.7 Hz, 1H), 6.92 (d, *J*=8.8 Hz, 2H), 6.79 (d, *J*=8.8 Hz, 1H), 4.59 - 4.39 (m, 4H), 4.02 (t, *J*=7.6 Hz, 2H), 3.84 (br s, 2H), 3.55 (br s, 2H), 2.82 (s, 2H), 2.65 - 2.54 (m, 2H), 2.17 - 2.02 (m, 4H). HRMS (ESI): m/z calcd for $C_{25}H_{27}N_2O_5$ $[M+H^+]$: 435.1904. Found 435.1929.

Scheme S4

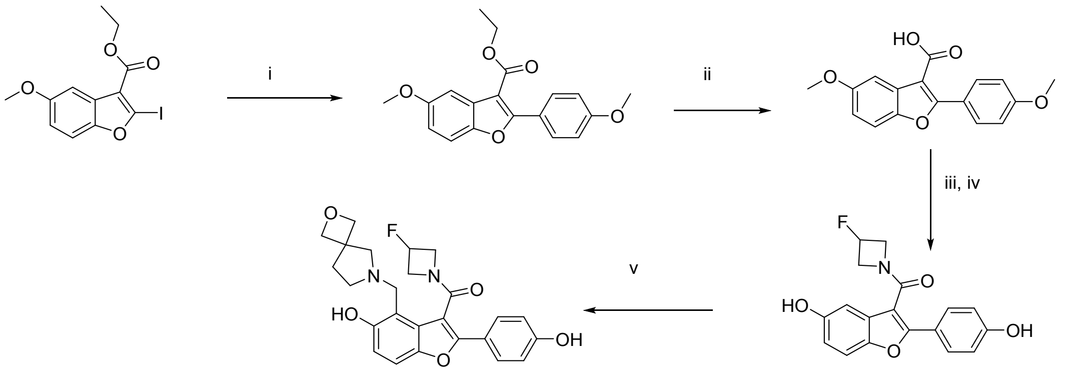

25

aReagents and conditions: (i) (4-methoxyphenyl)boronic acid, K_3PO_4, $Pd(PPh_3)_4$, dioxane, water, reflux, N_2; (ii) NaOH, water, EtOH, 80 °C ; (iii) 3-fluoroazetidine hydrochloride, HATU, DIPEA, THF, rt; (iv) BBr_3, DCM, -78 °C to rt; (v) formaldehyde, 2-oxa-7-azaspiro[3.4]octane; oxalic acid, DIPEA, EtOH, H_20, 80 °C.

Ethyl 5-methoxy-2-(4-methoxyphenyl)benzofuran-3-carboxylate

Ethyl 2-iodo-5-methoxybenzofuran-3-carboxylate, (1.5 g, 4.3 mmol), (4-methoxyphenyl)boronic acid (0.790 g, 5.2 mmol), K_3PO_4 (2.3 g, 10.83 mmol) and $Pd(PPh_3)_4$ (0.250 g, 0.22 mmol) were dissolved in 1,4-dioxane (15 mL) and water (3 mL). Reaction was then heated under reflux conditions under blanket of N_2 for 12 h. Reaction was cooled and water added. Reaction was extracted with DCM (x3). DCM extracts were combined and dried via hydrophobic filter and concentrated under reduced pressure. Product was purified by silica column chromatography (EtOAc 20% gradient elution with Heptane) to give the title compound (0.930 g, 2.7 mmol, 62%). LCMS: Rt m/z 327 $[M+H]^+$. ^1H NMR (400 MHz, DMSO) δ 7.97 (d, *J*=9.0 Hz, 2H), 7.59 (d, *J*=9.0 Hz, 1H), 7.46 (d, *J*=2.7 Hz, 1H), 7.10 (d, *J*=8.9 Hz, 2H), 7.00 (dd, *J*=2.7, 8.9 Hz, 1H), 4.34 (q, *J*=7.1 Hz, 2H), 3.86 - 3.83 (m, 6H), 1.34 (t, *J*=7.1 Hz, 3H).

5-Methoxy-2-(4-methoxyphenyl)benzofuran-3-carboxylic acid

Sodium hydroxide (0.48 g, 12 mmol) in water (6 mL) was added to a solution of ethyl 5-methoxy-2-(4-methoxyphenyl)benzofuran-3-carboxylate (0.75 g, 2.3 mmol) in ethanol (6 mL). Reaction was then heated at 80 ^{0}C for 12 h. Reaction was cooled and acidified with 2M HCl and extracted with EtOAc (x3). EtOAc extracts were combined and dried over anhydrous Na_2SO_4 and concentrated under reduced pressure to give the title compound (0.64 g, 2.04 mmol, 61%). LCMS: m/z 299 $[M+H]^+$. ^1H NMR (400 MHz, DMSO) δ 13.01 (s, 1H), 7.98 (d, *J*=8.9 Hz, 2H), 7.57 (d, *J*=8.9 Hz, 1H), 7.47 (d, *J*=2.6 Hz, 1H), 7.09 (d, *J*=8.9 Hz, 2H), 6.98 (dd, *J*=2.8, 8.9 Hz, 1H), 3.86 - 3.82 (m, 6H).

(3-fluoroazetidin-1-yl)(5-hydroxy-2-(4-hydroxyphenylbenzofuran-3-yl)methanone

To a solution of 5-methoxy-2-(4-methoxyphenyl)benzofuran-3-carboxylic acid (0.250 g, 0.83 mmol) and 3-fluoroazetidine hydrochloride (0.112 g, 1.01 mmol) in THF (5 mL) was added DIPEA (0.325g , 2.51 mmol) and HATU (0.350 g, 0.92 mmol). Reaction mixture was then stirred at room temperature for 12 h. Water was then added and reaction mixture extracted with EtOAc (x3). EtOAc extracts were combined, washed with brine, dried over anhydrous Na_2SO_4 and concentrated under reduced pressure. Product was purified by silica column chromatography using EtOAc – Heptane 1:1 to give a solid (0.270 g). A solution of the solid (0.270 g, 0.722 mmol) in DCM (7 mL) was cooled to -78 ^{0}C under N_2. BBr_3 (666 mg, 2.65 mmol) was then added dropwise and reaction was allowed to warm to room temperature over 12 h. Saturated $NaHCO_3$ was then added. DCM was separated and aqueous layer extracted with EtOAc (x3), dried over Na_2SO_4 and concentrated under reduced pressure to give the title compound (0.220 g, 0.605 mmol, 68%). LCMS: m/z 328 $[M+H]^+$.^1H NMR (400 MHz, DMSO) δ

10.06 (s, 1H), 9.34 (s, 1H), 7.67 (d, *J*=8.8 Hz, 2H), 7.42 (d, *J*=8.7 Hz, 1H), 6.95 - 6.90 (m, 3H), 6.77 (dd, *J*=2.5, 8.8 Hz, 1H), 5.45 - 5.26 (m, 1H), 4.35 - 4.35 (m, 1H), 4.16-3.92 (m, 2H), 3.72-3.57 (m, 1H)

(4-((2-oxa-6-azaspiro[3.4]octan-6-yl)methyl)-5-hydroxy-2-(4-hydroxyphenyl)benzofuran-3-yl)(3-fluoroazetidin-1-yl)methanone formate (25)

To a suspension of (3-fluoroazetidin-1-yl)-[5-hydroxy-2-(4-hydroxyphenyl)benzofuran-3-yl]methanone (50 mg, 0.15 mmol) and 2-oxa-7-azaspiro[3.4]octane; oxalic acid (34 mg, 0.16 mmol) in ethanol (1mL) and water (0.2 mL) were successively added DIPEA (29.614mg,0.2291mmol) and formaldehyde 37% solution in water (16.mg, 0.19 mmol) and the mixture was stirred at 80°C for 3 h. Saturated aqueous $NaHCO_3$ was added and the mixture was extracted with EtOAc, dried and concentrated to afford a residue. Purification by prep HPLC (acidic, 5-50% ACN) gave the title compound (29 mg, 0.05 mmol, 36%). LCMS: m/z 453 $[M+H]^+$. ^1H NMR (400 MHz, DMSO) δ 8.17 (d, *J*=2.8 Hz, 1H), 7.59 - 7.55 (m, 2H), 7.37 - 7.34 (m, 1H), 6.94 - 6.90 (m, 2H), 6.84 - 6.81 (m, 1H), 5.43-5.18 (m, 1H), 4.48 - 4.45 (m, 5H), 4.12-3.99 (m, 1H) 3.83 (br s, 3H), 3.59 (br s, 1H), 2.83 - 2.80 (m, 2H), 2.66-2.50 (m, 2H), 2.11 - 2.07 (m, 2H).

Scheme S5

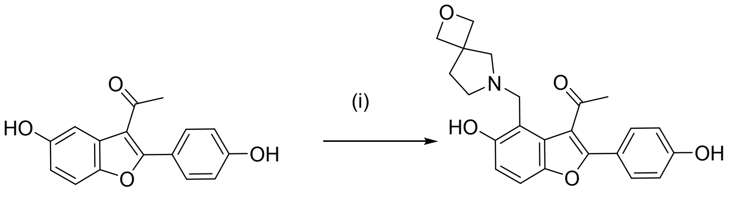

26

[a]*Reagents and conditions:* (i) Formaldehyde, 2-oxa-7-azaspiro[3.4]octane; oxalic acid, DIPEA, EtOH, H_20, 80 °C.

1-(4-((2-oxa-6-azaspiro[3.4]octan-6-yl)methyl)-5-hydroxy-2-(4-hydroxyphenyl)benzofuran-3-yl)ethan-1-one formate (26)

To a solution of 1-[5-hydroxy-2-(4-hydroxyphenyl)benzofuran-3-yl]ethanone (30 mg, 0.11 mmol) and 2-oxa-7-azaspiro[3.4]octane; oxalic acid (24 mg, 0.12 mmol) in ethanol (1 mL) and water (0.2 mL) was added DIPEA (21 mg, 0.16 mmol) and formaldehyde 37% solution in water (11 mg, 0.14 mmol) and the mixture was stirred at 80°C for 6 h. Saturated aqueous NaHCO3 was added and the mixture was extracted with EtOAc, dried and evaporated. The residue was purified by prep HPLC (acidic, 5-95% ACN) to give the title compound (11 mg, 0.02mmol, 21%). LCMS: Rt m/z 394 $[M+H]^+$. ^1H NMR (400 MHz, DMSO) δ 8.16 (s, 1H), 7.46 (d, *J*=8.7 Hz, 2H), 7.33 (d, *J*=8.7 Hz, 1H), 6.91 (d, *J*=8.7 Hz, 2H), 6.85 (d, *J*=8.8 Hz, 1H), 4.41 (s, 4H), 3.68 (s, 2H), 2.62 (s, 2H), 2.39 - 2.35 (m, 2H), 2.24 (s, 3H), 1.99 (t, *J*=7.0 Hz, 2H). HRMS (ESI): m/z calcd for $C_{23}H_{24}NO_5$ $[M+H^+]$: 394.1654. Found 394.1726.

Scheme S6

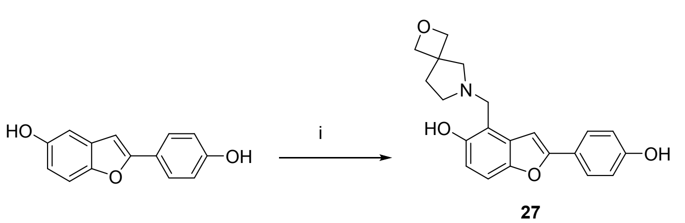

27

[a]*Reagents and conditions:* (i) Formaldehyde, 2-oxa-7-azaspiro[3.4]octane; oxalic acid, EtOH, H_20, 80°C.

4-((2-oxa-6-azaspiro[3.4]octan-6-yl)methyl)-2-(4-hydroxyphenyl)benzofuran-5-ol (27)

A mixture of 2-oxa-7-azaspiro[3.4]octane; oxalic acid (98 mg, 0.48 mmol), 2-(4-hydroxyphenyl)benzofuran-5-ol (100 mg, 0.44 mmol), formaldehyde 37% solution in water (46 mg, 0.57 mmol), in ethanol (3 mL) was heated at 80°C overnight. The cooled reaction mixture was treated with saturated $NaHCO_3$ (10ml), extracted with EtOAc (2 x 20 ml). The combined EtOAc extracts were evaporated in vacuo and half of the residue was purified by mass directed HPLC (basic, 5-95% ACN) to give the title compound (17 mg, 0.04 mmol, 9%) as a white solid. LCMS: m/z 352 $[M+H]^+$. ^1H NMR (400 MHz, DMSO) δ 9.96 - 9.82 (br s, 2H), 7.71 - 7.68 (m, 2H), 7.29 - 7.27 (m, 1H), 7.17 - 7.17 (m, 1H), 6.88 - 6.85 (m, 2H), 6.69 - 6.67 (m, 1H), 4.52 - 4.46 (m, 4H), 3.93 - 3.91 (m, 2H), 2.88 - 2.86 (m, 2H), 2.62 - 2.57 (m, 2H), 2.10 (t, *J*=6.7 Hz, 2H). HRMS (ESI): m/z calcd for $C_{22}H_{22}NO_4$ $[M+H^+]$: 352.1549. Found 352.1560.

Scheme S7

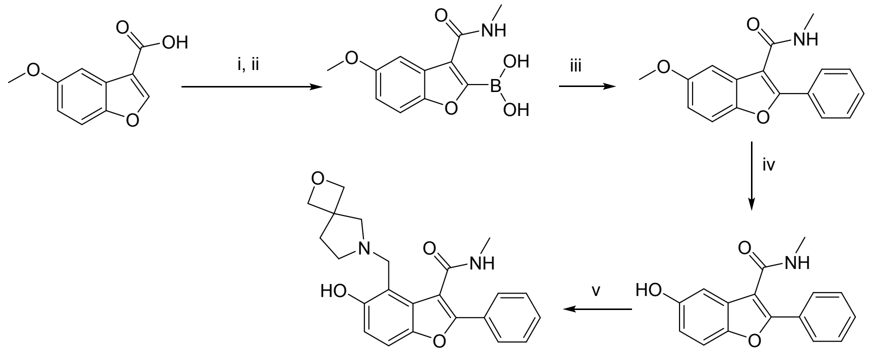

28

ᵃReagents and conditions: (i) methanamine hydrochloride, EDCI, Pyridine, 30 °C; (ii) trimethyl borate, LDA, THF, -78°C; (iii) bromobenzene, K_2CO_3, Pd(dppf)Cl_2, dioxane, water, 80 °C; (iv) pyridine hydrochloride, microwave, 180 °C; (v) formaldehyde, 2-oxa-7-azaspiro[3.4]octane; oxalic acid, EtOH, H_20, 80 °C.

(5-methoxy-3-(methylcarbamoyl)benzofuran-2-yl)boronic acid

A solution of 5-methoxybenzofuran-3-carboxylic acid (94.00 g, 489.15 mmol) methanamine;hydrochloride (46.38 g, 686.89 mmol) and EDCI (219.46 g, 1.14 mol) in pyridine (1.0 L) was stirred at 30 °C for 16 h. The reaction was poured into water (2 L) and stirred for 15 min. The aqueous phase was extracted with ethyl acetate (300 mL x 5). The combined organic phase was washed with brine (300 mL x 2), dried over anhydrous Na_2SO_4, filtered and concentrated under vacuum to give crude product. The residue solid was purified through slurry in MTBE/PE=5/1 (600 ml) to give a white solid (67.00 g). To a solution the white solid (67.00 g, 326.30 mmol) and trimethyl borate (135.62 g, 1.30 mol) in THF (600 mL) was added LDA (2 M, 685.23 mL) drop-wise at -78 °C under N_2. The reaction was stirred at -78 °C for 2 h. The reaction mixture was poured into aqueous HCl solution (2 M, 1.5 L). The solid was collected, washed with water (500 mL x 3) and dried under vacuum to give the title compound (81.0 g, 326.65 mmol, 99%) as a white solid. LCMS: m/z 250 $[M+H]^+$. ^1H NMR (400MHz, DMSO) δ 8.54 (d, *J*=4.4 Hz, 1H), 7.59 (d, *J*=8.8 Hz, 1H), 7.43 (d, *J*=2.4 Hz, 1H), 7.07-7.04 (m, 1H), 3.85 (s, 3H), 2.91 (d, *J*=4.8 Hz, 3H).

5-methoxy-N-methyl-2-phenylbenzofuran-3-carboxamide

Potassium carbonate (1.665 g, 12 mmol), Pd(dppf)Cl_2 (327 mg, 0.40 mmol), bromobenzene (630 mg, 4.0156mmol) and [5-methoxy-3-(methylcarbamoyl)benzofuran-2-yl]boronic acid (1 g, 4.01 mmol) were mixed in water (4 mL) / 1,4-dioxane (20 mL) and the reaction mixture was degassed by

bubbling N_2 through the reaction mixture for 10mins and purged with N_2. The reaction mixture was heated at 80 °C for 3 h. The reaction mixture was cooled and quenched with water (10 ml) and then extracted with EtOAc. (2 x 15 ml) and evaporated in vacuo and the residue purified by silica (40g) eluting with 0-50% EtOAc/ heptane to give the title compound (640 mg, 2.16 mmol, 53%) as a brown solid. LCMS: m/z 282 $[M+H]^+$. ^1H NMR (500 MHz, DMSO) δ 8.45 - 8.40 (m, 1H), 7.89 - 7.86 (m, 2H), 7.60 - 7.46 (m, 4H), 7.09 (d, J=2.6 Hz, 1H), 6.99 (dd, J=2.6, 8.9 Hz, 1H), 3.82 (s, 3H), 2.85 - 2.84 (m, 3H).

5-hydroxy-N-methyl-2-phenylbenzofuran-3-carboxamide

A mixture of 5-methoxy-N-methyl-2-phenyl-benzofuran-3-carboxamide (240 mg, 0.85 mmol), pyridine hydrochloride (492 mg, 4.26 mmol) was heated at 180 $^{°C}$ in the microwave for 30 mins. The reaction mixture was purified by SCX (10g) and eluted with MeOH. The MeOH fractions were evaporated in vacuo. The solid was purified by silica (12 g, 0-100% EtOAc/heptane) to give the title compound as a light brown solid (72 mg, 0.25 mmol, 29%). LCMS: m/z 268 $[M+H]^+$. ^1H NMR (400 MHz, DMSO) δ 9.36 (s, 1H), 8.39 (m, 1H), 7.85 (d, *J*=7.2 Hz, 2H), 7.53 - 7.42 (m, 4H), 6.94 (d, *J*=2.3 Hz, 1H), 6.82 (dd, *J*=2.5, 8.8 Hz, 1H), 2.83 - 2.81 (m, 3H).

4-((2-oxa-6-azaspiro[3.4]octan-6-yl)methyl)-5-hydroxy-N-methyl-2-phenylbenzofuran-3-carboxamide (28)

A mixture of 5-hydroxy-N-methyl-2-phenyl-benzofuran-3-carboxamide (50 mg, 0.18 mmol) 2-oxa-7-azaspiro[3.4]octane; oxalic acid (57 mg, 0.28 mmol) in ethanol (2 mL) and water (0.4 mL) was treated with DIPEA (79 mg,0. 61 mmol) and formaldehyde 37% solution in water (106 mg, 1.30 mmol) and heated at 80°C overnight. The cooled reaction mixture was evaporated in vacuo and the residue purified by mass directed HPLC (basic, 5-95% ACN) to give the title compound (38 mg, 0.09 mmol, 49%) as a yellow glassy solid. LCMS: m/z 393 $[M+H]^+$. ^1H NMR (500 MHz, DMSO) δ 10.66 (s, 1H), 8.63 - 8.58 (m, 1H), 7.76 - 7.74 (m, 2H), 7.53 - 7.49 (m, 2H), 7.46 - 7.40 (m, 2H), 6.82 - 6.80 (m, 1H), 4.50 - 4.46 (m, 4H), 3.86 (s, 2H), 2.83 - 2.77 (m, 5H), 2.58-2.53 (m, 2H), 2.11-2.06 (m, 2H). HRMS (ESI): m/z calcd for $C_{23}H_{25}N_2O_4$ $[M+H^+]$: 393.1814. Found 393.1616.

Scheme S8

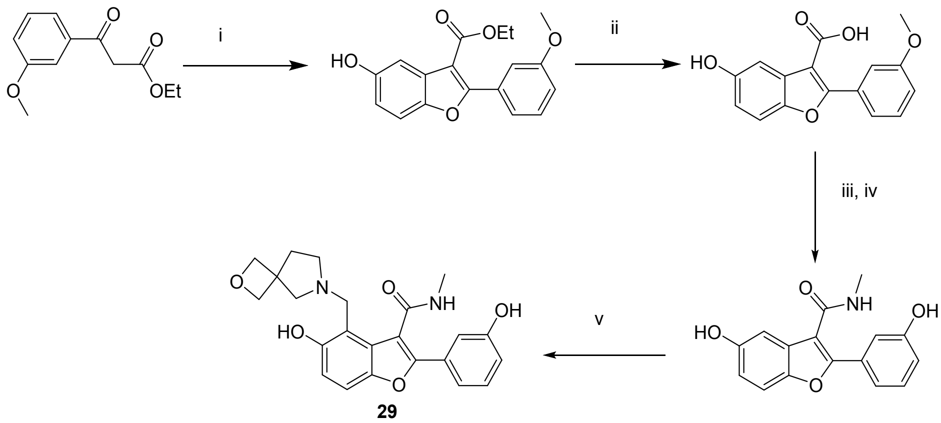

29

[a]*Reagents and conditions:* (i) 1,4-benzoquinone, $ZnCl_2$, toluene, 110 °C ; (ii) NaOH, EtOH, H_20, 50 °C; (iii) methanamine hydrochloride, HOBt, EDCI, DIPEA, DMF, 15 °C; (iv) BBr_3, DCM, 0 °C to 15 °C; (v) formaldehyde, 2-oxa-7-azaspiro[3.4]octane; oxalic acid, dioxane, H_20, 80 °C.

ethyl 5-hydroxy-2-(3-methoxyphenyl)benzofuran-3-carboxylate

To a mixture of ethyl 3-(3-methoxyphenyl)-3-oxopropanoate (17.50 g, 78.74 mmol) and 1, 4-benzoquinone (9.36 g, 86.61 mmol, 19.50 mL) in toluene (175 mL) was added $ZnCl_2$ (11.81 g, 86.61

mmol, 4.06 mL) in one portion at 15 °C. The mixture was stirred at 110 °C for 16 h. The reaction mixture was concentrated under reduced pressure to remove solvent. The residue was diluted with water (100 mL) and extracted with ethyl acetate (50 mL x 3). The combined organic layers were washed with brine (50 mL), dried over anhydrous Na_2SO_4, filtered and concentrated under reduced pressure to give a residue. The residue was purified by column chromatography (SiO_2, Petroleum ether/Ethyl acetate=30/1 to 1/1) to give the title compound as a yellow oil. (10.00 g, 28.82 mmol, 36%) LCMS: m/z 313 $[M+H]^+$. ^1H NMR (400 MHz, DMSO) δ 9.48 (s, 1H), 7.53 - 7.50 (m, 3H), 7.49 - 7.48 (m, 1H), 7.43 - 7.36 (d , 1H), 7.15 - 7.05 (m, 1H), 6.86 - 6.84 (d, 1H), 4.34 - 4.28 (m, 2H), 3.82 (s, 3H), 1.32 - 1.29 (t, 3H)

5-hydroxy-2-(3-methoxyphenyl)benzofuran-3-carboxylic acid

To a solution of ethyl 5-hydroxy-2-(3-methoxyphenyl)benzofuran-3-carboxylate (10.00 g, 32.02 mmol) in EtOH (50 mL) was added a solution of NaOH (3.84 g, 96.06 mmol) in H_2O (50 mL) at 15 °C. The mixture was stirred at 50 °C for 16 h. The reaction mixture was concentrated under reduced pressure. The residue was diluted with water (300 mL), acidified with conc.HCl untill pH =4~5 and extracted with ethyl acetate (100 mL * 3). The combined organic layers were washed with brine (150 mL), dried over anhydrous Na_2SO_4, filtered and concentrated under reduced pressure to give a residue. The residue was purified by re-crystallization from MTBE (100 mL) to give the title compound as a light yellow solid. (8.50 g, 28.71mmol, 89%) LCMS: m/z 307 $[M+Na]^+$. ^1H NMR (400 MHz, DMSO) δ 7.62 (s, 1H), 7.53 - 7.46 (d, 1H), 7.44 - 7.39 (m, 3H), 7.07 - 7.04 (m , 1H), 6.84 - 6.81 (m, 1H), 3.81 (s, 3H)

5-hydroxy-2-(3-hydroxyphenyl)-N-methylbenzofuran-3-carboxamide

To a mixture of compound 5-hydroxy-2-(3-methoxyphenyl)benzofuran-3-carboxylic acid (1.90 g, 6.68 mmol), HOBt (1.35 g, 10.03 mmol) and EDCI (1.92 g, 10.03 mmol) in DMF (19 mL) was added methanamine hydrochloride (676 mg, 10.03 mmol) and DIPEA (2.59 g, 20.05 mmol, 3.50 mL) at 0 °C. The mixture was stirred at 15 °C for 12 h. The mixture was diluted with water (60 mL) and extracted with ethylacetate (30 mL x 3). The combined organic layers were washed with brine (30 mL), dried over anhydrous Na_2SO_4, filtered and concentrated under reduced pressure to give a brown solid (2.00g, crude). To a solution of the brown solid (2.00 g, 6.73 mmol) in DCM (20 mL) was added BBr_3 (5.06 g, 20.19 mmol, 1.95 mL) at 0 °C under N_2. The mixture was stirred at 15 °C for 4 h. The reaction solution was quenched by aqueous $NaHCO_3$ (20 mL), then adjusted to pH of 4~5 with conc. HCl and filtered. The filtered cake was washed with water (20 mL), dried in vacuum to give the title compound as a brown solid (1.70 g, 5.10 mmol, 76%, 85% purity). LCMS: m/z 284 $[M+H]^+$. ^1H NMR (400 MHz, DMSO) δ 8.38 - 8.37 (q, 1H), 7.45 - 7.42 (d, 1H), 7.29 - 7.26 (m, 3H), 6.90 - 6.89 (d, 1H), 6.83 - 6.80 (m, 2H), 2.81 - 2.80 (d, 3H)

4-((2-oxa-6-azaspiro[3.4]octan-6-yl)methyl)-5-hydroxy-2-(3-hydroxyphenyl)-N-methylbenzofuran-3-carboxamide formate (29)

To a solution of 5-hydroxy-2-(3-hydroxyphenyl)-N-methylbenzofuran-3-carboxamide (100 mg, 353 µmol) and formaldehyde 37% solution in water (31 mg, 388 µmol, 28 µL) in dioxane (1.5 mL) and H_2O (0.5 mL) was added 2-oxa-6-azaspiro[3.4]octane oxalic acid (86 mg, 423 µmol). The reaction mixture was stirred at 80 °C for 30 h. The reaction mixture was concentrated under reduced pressure. The residue was purified by prep-HPLC (Formic Acid condition) to give the title compound (15 mg, 32 µmol, 9%) as a brown solid. LCMS: m/z 409 $[M+H]^+$. ^1H NMR (400 MHz, DMSO) δ 8.59 (q, *J*=4.6 Hz, 1H), 8.19 (s, 1H), 7.41 - 7.37 (m, 1H), 7.28 (t, *J*=8.2 Hz, 1H), 7.20 - 7.16 (m, 2H), 6.83 - 6.78 (m, 2H), 4.51 - 4.46 (m, 4H), 3.85 (s, 2H), 2.84 - 2.77 (m, 5H), 2.59-2.53 (m, 2H), 2.09 (t, *J*=7.1 Hz, 2H).

Scheme S9

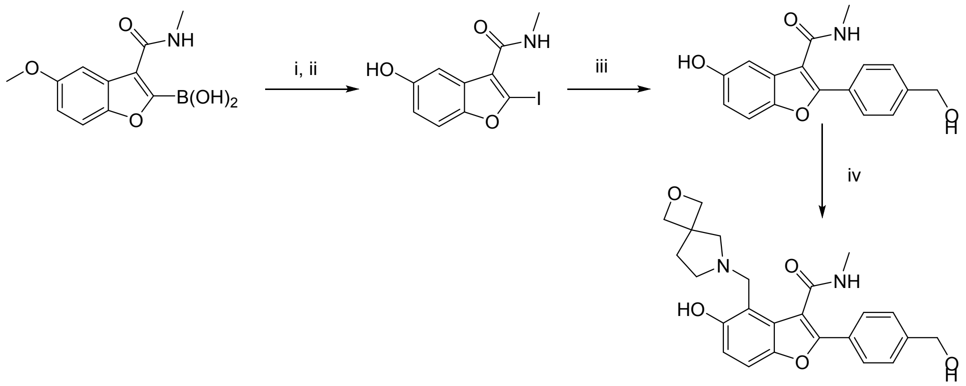

30

[a]Reagents and conditions: (i) NIS, DMF, 25°C; (ii) BBr_3, DCM, 0 °C; (iii) [4-(hydroxymethyl)phenyl]boronic acid, K_3PO_4 , $Pd(PPh_3)_4$, dioxane, water, N_2, 100°C; (iv) formaldehyde, 2-oxa-7-azaspiro[3.4]octane; oxalic acid, EtOH, H_20, 80 °C.

5-hydroxy-2-iodo-N-methylbenzofuran-3-carboxamide

A mixture of (5-methoxy-3-(methylcarbamoyl)benzofuran-2-yl)boronic acid (81.00 g, 321.25 mmol) and NIS (79.50 g, 353.38 mmol) in DMF (800 mL) under N_2 was stirred at 25 °C for 16 h. The reaction mixture was poured into water (1.5 L). The solid was collected, washed with H_2O (200 mL x 3) and dried in vacuum to afford 2-iodo-5-methoxy-N-methylbenzofuran-3-carboxamide (60.00 g, 181.21 mmol, 56%) as a white solid. To a solution of 2-iodo-5-methoxy-N-methylbenzofuran-3-carboxamide (40.00 g, 120.81 mmol) in DCM (400 mL) was added BBr_3 (121.06 g, 483.24 mmol, 46.56 mL) drop-wise at 0 °C under N_2. The reaction was stirred at 30 °C for 12 h. The reaction solution was quenched with EtOH (20 mL) at 0 °C. The mixture was filtered, the filter cake was washed with H_2O (50 mL x 3) and dried in vacuum to afford the title compound (38.00 g, 119.84 mmol, 99%) as a white solid. LCMS: m/z 318 $[M+H]^+$. ^1H NMR (400MHz, DMSO) δ 9.42 (s, 1H), 8.04 (d, *J*=4.3 Hz, 1H), 7.43 (d, *J*=9.0 Hz, 1H), 7.04 (d, *J*=2.4 Hz, 1H), 6.75 (dd, *J*=2.4, 8.9 Hz, 1H), 2.81 (d, *J*=4.6 Hz, 3H).

5-hydroxy-2-(4-(hydroxymethyl)phenyl)-N-methylbenzofuran-3-carboxamide

A mixture of 5-hydroxy-2-iodo-N-methyl-benzofuran-3-carboxamide (200 mg, 0.63 mmol), $Pd(PPh_3)_4$ (36 mg, 0.03mmol), K_3PO_4 (334 mg, 1.57mmol) and [4-(hydroxymethyl)phenyl]boronic acid (115 mg, 0.75 mmol) in 1,4-dioxane (4 mL) and water (0.8 mL) was degassed under N_2 for 5 min and then heated at 100°C for 2 h. Water was added and the mixture was extracted with EtOAc (x3), dried and concentrated to give a residue, which was purified by silica column chromatography (50-100% EtOAc in heptane) to give the title compound (94 mg, 0.30mmol, 47%). LCMS: m/z 298 $[M+H]^+$. ^1H NMR (400 MHz, DMSO) δ 9.35 (s, 1H), 8.35 (d, *J*=4.6 Hz, 1H), 7.83 - 7.79 (m, 2H), 7.44 (m, 3H), 6.93 (d, *J*=2.2 Hz, 1H), 6.80 (dd, *J*=2.3, 8.8 Hz, 1H), 5.30 (t, *J*=5.7 Hz, 1H), 4.56 (d, *J*=5.7 Hz, 2H), 2.81 (d, *J*=4.6 Hz, 3H).

4-((2-oxa-6-azaspiro[3.4]octan-6-yl)methyl)-5-hydroxy-2-(4-(hydroxymethyl)phenyl)-N-methylbenzofuran-3-carboxamide (30)

To a solution of 5-hydroxy-2-(4-(hydroxymethyl)phenyl)-N-methylbenzofuran-3-carboxamide (40 mg, 0.13 mmol) and 2-oxa-7-azaspiro[3.4]octane; oxalic acid (30 mg, 0.14 mmol) in ethanol (1 mL) and water (0.2 mL) was added DIPEA (22 mg, 0.17 mmol) and formaldehyde 37% solution in water (13 mg, 0.16 mmol) and the mixture was stirred at 80°C overnight. Saturated aqueous $NaHCO_3$ was added and the mixture was extracted with EtOAc, dried and concentrated to afford a residue. Purification by prep HPLC (basic, 5-95% ACN) gave the title compound (12 mg, 0.02 mmol, 20%). LCMS: m/z 423 $[M+H]^+$. ^1H NMR (400 MHz, DMSO) δ 8.59 (q, *J*=4.5 Hz, 1H), 7.71 - 7.68 (m, 2H), 7.46 - 7.38 (m, 3H), 6.81 - 6.78

(m, 1H), 5.34 - 5.29 (m, 1H), 4.57 - 4.45 (m, 6H), 3.86 (s, 2H), 2.84 - 2.76 (m, 5H), 2.59-2.52 (m,2H), 2.12 - 2.06 (m, 2H).

Scheme S10

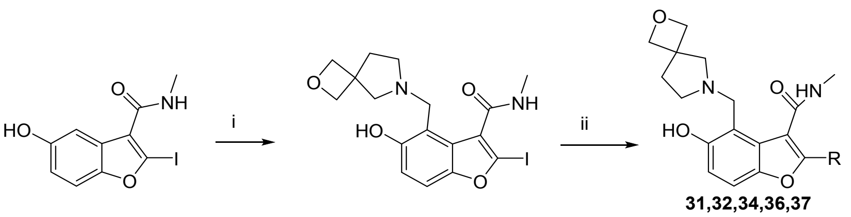

31,32,34,36,37

[a]*Reagents and conditions:* (i) Formaldehyde, 2-oxa-7-azaspiro[3.4]octane oxalic acid, EtOH, water, 60°C; (ii) Boronic acid, K_3PO_4, Pd(dppf)Cl_2, dioxane, water, 80 °C, N_2, or boronic acid, Na_2CO_3, Pd(dppf)Cl_2, dioxane, water, microwave, 120 °C, N_2.

4-((2-oxa-6-azaspiro[3.4]octan-6-yl)methyl)-5-hydroxy-2-iodo-N-methylbenzofuran-3-carboxamide

A solution of 5-hydroxy-2-iodo-N-methylbenzofuran-3-carboxamide (5.00 g, 15.77 mmol), 2-oxa-7-azaspiro[3.4]octane oxalic acid (3.52 g, 17.35 mmol) and formaldehyde (1.41 g, 17.35 mmol, 1.29 mL, 37% solution in water) in EtOH (100.00 mL) and H_2O (20.00 mL) was stirred at 60 °C for 40 h. The reaction was concentrated under vacuum. The residue was purified by re-crystallization from MeOH/EtOAc (1/1,10 mL) to get crude product (3.10 g) as a white solid. The combined crude product (16 g, 5 batches) was purified by re-crystallization from MeOH/EtOAc/H_2O (1/1/1, 20 mL) to give the title compound (11.00 g, 23.63 mmol, 65%) as a white solid. LCMS: m/z 443 $[M+H]^+$. ^1H NMR: (400MHz, MeOD) δ 8.67 (d, *J*=4.8 Hz, 1H), 7.54 (d, *J*=9.2 Hz, 1H), 6.97 (d, *J*=9.6 Hz, 1H), 4.56-4.55 (m, 2H), 4.48-4.47 (m, 2H), 4.26 (s, 2H), 3.48 (s, 2H), 3.21 (s, 2H), 2.85 (d, *J*=4.8 Hz, 3H), 2.32-2.28 (m, 2H).

General Procedure A: A mixture of 5-hydroxy-2-iodo-N-methyl-4-(2-oxa-7-azaspiro[3.4]octan-7-ylmethyl)benzofuran-3-carboxamide (96 mg, 0.21 mmol), K_3PO_4 (115 mg, 0.54 mmol), the corresponding boronic acid (32 mg, 0.21 mmol), in 1,4-dioxane (4 mL) and water (1 mL) was degassed for 10mins. Pd(dppf)Cl_2 (14 mg, 0.018 mmol) was added, the flask flushed with nitrogen and the reaction mixture heated at 80°C for 2 h. The cooled reaction mixture was partitioned between water (5 ml) and extracted with EtOAc (2 x 10 ml) or DCM and evaporated in vacuo. The residue purified by mass directed HPLC (acidic, 5-95% ACN).

4-((2-oxa-6-azaspiro[3.4]octan-6-yl)methyl)-5-hydroxy-2-(4-methoxyphenyl)-N-methylbenzofuran-3-carboxamide formate (31)

Prepared using general procedure A starting from (4-methoxyphenyl)boronic acid (32 mg, 0.21 mmol) to give the title compound (60 mg, 0.12 mmol, 56%) as a white solid. LCMS: m/z 423 $[M+H]^+$. ^1H NMR (400 MHz, DMSO) δ 8.57 (q, *J*=4.6 Hz, 1H), 8.16 (s, 1H), 7.70 - 7.67 (m, 2H), 7.39 - 7.36 (m, 1H), 7.09 - 7.06 (m, 2H), 6.79 - 6.76 (m, 1H), 4.51 - 4.46 (m, 4H), 3.86 (s, 2H), 3.82 (s, 3H), 2.85 (s, 2H), 2.77 (d, *J*=4.7 Hz, 3H), 2.59-2.54 (m, 2H), 2.12-2.07 (m, 2H). HRMS (ESI): m/z calcd for $C_{24}H_{27}N_2O_5$ $[M+H^+]$: 423.1920. Found 423.1964.

4-((2-oxa-6-azaspiro[3.4]octan-6-yl)methyl)-5-hydroxy-2-(1H-indol-5-yl)-N-methylbenzofuran-3-carboxamide (32)

Prepared using general procedure A starting from 5-(4,4,5,5-tetramethyl-1,3,2-dioxaborolan-2-yl)-1H-indole (54 mg, 0.22 mmol) , 5-hydroxy-2-iodo-N-methyl-4-(2-oxa-7-azaspiro[3.4]octan-7-ylmethyl)benzofuran-3-carboxamide (100 mg, 0.22 mmol) and K_3PO_4 (143 mg, 0.67 mmol) and

Pd(dppf)Cl_2 (18 mg, 0.02 mmol). Purification by prep HPLC (XBridge column, 0.1% NH_4OH modifier) gave the title compound as a white solid (50mg, 0.11mmol, 48%). LCMS: m/z 432 [M+H]. ^{1}H NMR (400 MHz, DMSO) δ 11.31 (s, 1H), 8.53 (q, *J*=4.5 Hz, 1H), 7.98 - 7.97 (m, 1H), 7.50 (s, 2H), 7.44 - 7.36 (m, 2H), 6.76 - 6.73 (m, 1H), 6.54 (d, *J*=2.0 Hz, 1H), 4.51 - 4.46 (m, 4H), 3.87 (s, 2H), 2.84 (s, 2H), 2.79 - 2.77 (m, 3H), 2.59-2.54 (m, 2H), 2.13 - 2.08 (m, 2H). HRMS (ESI): m/z calcd for $C_{25}H_{26}N_3O_4$ [M+H$^+$]: 432.1923. Found 432.1891.

2-(3-fluoro-4-hydroxy-phenyl)-5-hydroxy-2-*N*-methyl-4-(2-oxa-7-azaspiro[3.4]octan-7-ylmethyl)benzofuran-3-carboxamide (34)

To a solution of 5-hydroxy-2-iodo-*N*-methyl-4-(2-oxa-7-azaspiro[3.4]oxtan-7-ylmethyl)benzofuran-3-carboxamide (150 mg, 0.34 mmol) in 1,4-dioxane (4 mL) and water (0.5 mL) was added sodium carbonate (72 mg, 0.68 mmol). Nitrogen was bubbled through the suspension for 5 mins, after which 3-fluoro-4-hydroxyphenylboronic acid (79 mg, 0.51 mmol) and [1,1'-Bis(diphenylphosphino)ferrocene]dichloropalladium(II) (12 mg, 0.02 mmol) were added. The reaction was heated by microwave irradiation at 120 °C for 1 h. Water was added and the mixture was extracted three times with CH_2Cl_2 and filtered through a phase separator. The filtrate was reduced *in vacuo* and the residue was purified by preparative HPLC (basic, 0-95% ACN). Further purification carried out by HPLC (basic, 0-50% ACN) to give the title compound (27 mg, 0.06 mmol, 18%). LCMS: m/z 427 [M+H]$^+$. ^{1}H NMR (400 MHz, DMSO) δ 10.52 (br s, 2H), 8.59 (q, *J*=4.5 Hz, 1H), 7.48 - 7.34 (m, 3H), 7.06 (t, *J*=8.8 Hz, 1H), 6.79 - 6.75 (m, 1H), 4.50 - 4.45 (m, 4H), 3.83 (s, 2H), 2.83 - 2.77 (m, 5H), 2.57-2.52 (m, 2H), 2.11 - 2.06 (m, 2H). HRMS (ESI): m/z calcd for $C_{23}H_{24}N_2O_5F$ [M+H$^+$]: 427.1669. Found 427.1772.

4-((2-oxa-6-azaspiro[3.4]octan-6-yl)methyl)-2-(2,6-difluoro-4-hydroxyphenyl)-5-hydroxy-N-methylbenzofuran-3-carboxamide formate (36)

Using general procedure A starting from 5-hydroxy-2-iodo-N-methyl-4-(2-oxa-7-azaspiro[3.4]octan-7-ylmethyl)benzofuran-3-carboxamide (100 mg, 0.22 mmol), (2,6-difluoro-4-hydroxy-phenyl)boronic acid (39 mg, 0.22 mmol), Pd(dppf)Cl_2 (18 mg, 0.02 mmol) and K_3PO_4 (48 mg, 0.22 mmol) in 1,4-dioxane (1.6 mL) and water (0.4 mL). Purification by prep LCMS (XBridge column, 0.1% formic acid modifier, 5-95% MeCN in H_2O) gave the title compound as a brown solid (25 mg, 0.05 mmol, 21%). LCMS: m/z 445 [M+H]$^+$. ^{1}H NMR (400 MHz, DMSO) δ 8.18 - 8.16 (m, 2H), 7.38 (d, *J*=8.8 Hz, 1H), 6.85 (d, *J*=8.9 Hz, 1H), 6.59 (d, *J*=10.0 Hz, 2H), 4.47 - 4.43 (m, 4H), 3.97 (s, 2H), 2.84 (s, 2H), 2.65 (d, *J*=4.6 Hz, 3H), 2.59 - 2.54 (m, 2H), 2.07 (m, 2H). HRMS (ESI): m/z calcd for $C_{23}H_{23}N_2O_5F_2$ [M+H$^+$]: 445.1575. Found 445.1564.

4-((2-oxa-6-azaspiro[3.4]octan-6-yl)methyl)-5-hydroxy-2-(4-hydroxy-3-methylphenyl)-N-methylbenzofuran-3-carboxamide (37)

Using general procedure A starting from 5-hydroxy-2-iodo-N-methyl-4-(2-oxa-7-azaspiro[3.4]octan-7-ylmethyl)benzofuran-3-carboxamide (82 mg, 0.18 mmol), (4-hydroxy-3-methyl-phenyl)boronic acid (28 mg, 0.18 mmol), Pd(dppf)Cl_2 (15 mg, 0.01 mmol) and K_3PO_4 (118 mg, 0.55 mmol) in dioxane (2 mL) and water (0.5 mL). Purifiication by prep LCMS (XBridge column, 0.1% NH4OH modifier, 5-95% MeCN in H_2O) gave the title compound as a brown solid (47 mg, 0.10 mmol, 57%). LCMS: m/z 423 [M+H]$^+$. ^{1}H NMR (400 MHz, DMSO) δ 8.49 (q, *J*=4.6 Hz, 1H), 7.45 (d, *J*=1.7 Hz, 1H), 7.40 - 7.31 (m, 2H), 6.87 - 6.83 (m, 1H), 6.73 - 6.70 (m, 1H), 4.50 - 4.44 (m, 4H), 3.83 (s, 2H), 3.17 (s, 1H), 2.82 - 2.74 (m, 5H), 2.57-2.50 (m, 2H), 2.16 (s, 3H), 2.08 (t, *J*=7.2 Hz, 2H). HRMS (ESI): m/z calcd for $C_{24}H_{27}N_2O_5$ [M+H$^+$]: 423.1920. Found 423.2954.

Scheme S11

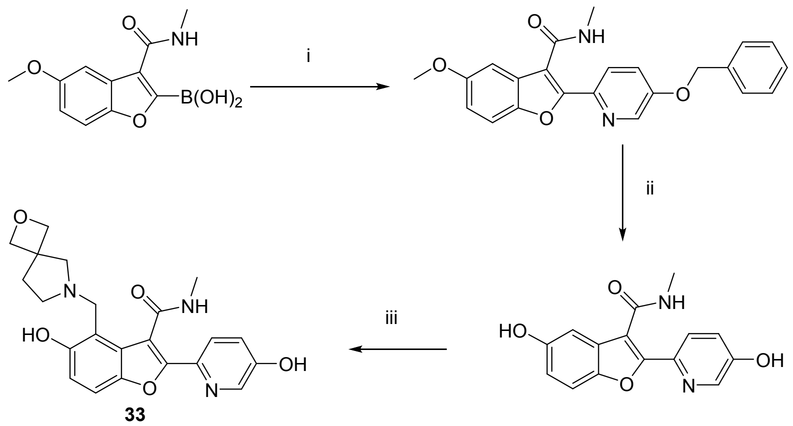

ᵃReagents and conditions: (i) 5-benzyloxy-2-bromo-pyridine, K_2CO_3, $Pd(dppf)Cl_2$, dioxane, water, 80 °C; (ii) BBr_3, DCM, -78 °C to rt; (iii) formaldehyde, 2-oxa-7-azaspiro[3.4]octane oxalic acid, EtOH, water, reflux.

2-(5-(benzyloxy)pyridin-2-yl)-5-methoxy-N-methylbenzofuran-3-carboxamide

(5-methoxy-3-(methylcarbamoyl)benzofuran-2-yl)boronic acid (0.4 g, 1.61 mmol), 5-benzyloxy-2-bromo-pyridine (0.424 g, 1.61 mmol), potassium carbonate (0.444 g, 3.21 mmol) and $Pd(dppf)Cl_2$ (0.132 g, 0.16 mmol) were added to 1,4-dioxane (8 mL) and water (2 mL). Reaction vessel was degassed and purged with N_2 then heated at 80 ^{0}C for 2 h. Reaction was cooled then water added and extracted with EtOAc. The EtOAc extracts were combined and washed with brine, dried over anhydrous Na_2SO_4 and concentrated under reduced pressure. Product was purified by silica column chromatography using 10% EtOAc – Heptane to give the title compound (0.37 g, 0.90 mmol, 56%). LCMS: m/z 389 $[M+H]^+$. ^{1}H NMR (400 MHz, DMSO) δ 10.79 (d, *J*=4.5 Hz, 1H), 8.62 (d, *J*=2.8 Hz, 1H), 8.12 - 8.09 (m, 1H), 7.79 - 7.75 (m, 2H), 7.59 - 7.38 (m, 6H), 7.01 (dd, *J*=2.7, 8.9 Hz, 1H), 5.33 (s, 2H), 3.82 (s, 3H), 2.90 (d, *J*=4.7 Hz, 3H).

5-hydroxy-2-(5-hydroxypyridin-2-yl)-N-methylbenzofuran-3-carboxamide

2-(5-(benzyloxy)pyridin-2-yl)-5-methoxy-N-methylbenzofuran-3-carboxamide (0.25 g, 0.64 mmol) was added to DCM (6 mL) and cooled to -78 ^{0}C under N_2. BBr_3 (0.806 g, 3.21 mmol) was then added slowly and reaction warmed to room temperature over 12 h. Saturated $NaHCO_3$ was then added and DCM layer separated. Aqueous $NaHCO_3$ was extracted with EtOAc (x3). Organic extracts were combined, dried over anhydrous Na_2SO_4 and concentrated under reduced pressure to give the title compound (0.175 g, 0.55 mmol, 86%). LCMS: m/z 285 $[M+H]^+$. ^{1}H NMR (400 MHz, DMSO) δ 10.93 - 10.91 (m, 1H), 10.77 (s, 1H), 9.32 (s, 1H), 8.34 (d, *J*=2.8 Hz, 1H), 8.01 - 7.98 (m, 1H), 7.66 (d, *J*=2.6 Hz, 1H), 7.47 - 7.41 (m, 2H), 6.82 (dd, *J*=2.5, 8.8 Hz, 1H), 2.88 (d, *J*=4.5 Hz, 3H).

4-((2-oxa-6-azaspiro[3.4]octan-6-yl)methyl)-5-hydroxy-2-(5-hydroxypyridin-2-yl)-N-methylbenzofuran-3-carboxamide formate (33)

To a suspension of 5-hydroxy-2-(5-hydroxy-2-pyridyl)-N-methyl-benzofuran-3-carboxamide (50 mg, 0.17 mmol), 2-oxa-7-azaspiro[3.4]octane; oxalic acid (39 mg, 0.19 mmol) and DIPEA (29 mg, 0.22 mmol) in ethanol (1 mL) and water (0.2 mL) was added formaldehyde 37% solution in water (15 mg, 0.19 mmol) and the mixture was refluxed for 24 h. Saturated aqueous $NaHCO_3$ was added and the mixture was extracted with EtOAc, dried and concentrated. The residue was purified by prep HPLC (acidic, 5-50% ACN) to give the title compound (12 mg, 0.02 mmol, 14%). LCMS: m/z 410 $[M+H]^+$. ^{1}H NMR (400 MHz, DMSO) δ 8.37 (q, *J*=4.5 Hz, 1H), 8.21 - 8.16 (m, 2H), 7.68 - 7.65 (m, 1H), 7.40 - 7.37

(m, 1H), 7.28 (dd, *J*=2.8, 8.7 Hz, 1H), 6.81 - 6.78 (m, 1H), 4.51 - 4.46 (m, 4H), 3.89 (s, 2H), 2.85 (s, 2H), 2.77 - 2.75 (m, 3H), 2.61-2.55 (m, 2H), 2.12 - 2.07 (m, 2H). HRMS (ESI): m/z calcd for $C_{22}H_{24}N_3O_5$ [M+H$^+$]: 410.1716. Found 410.1612.

Scheme S12

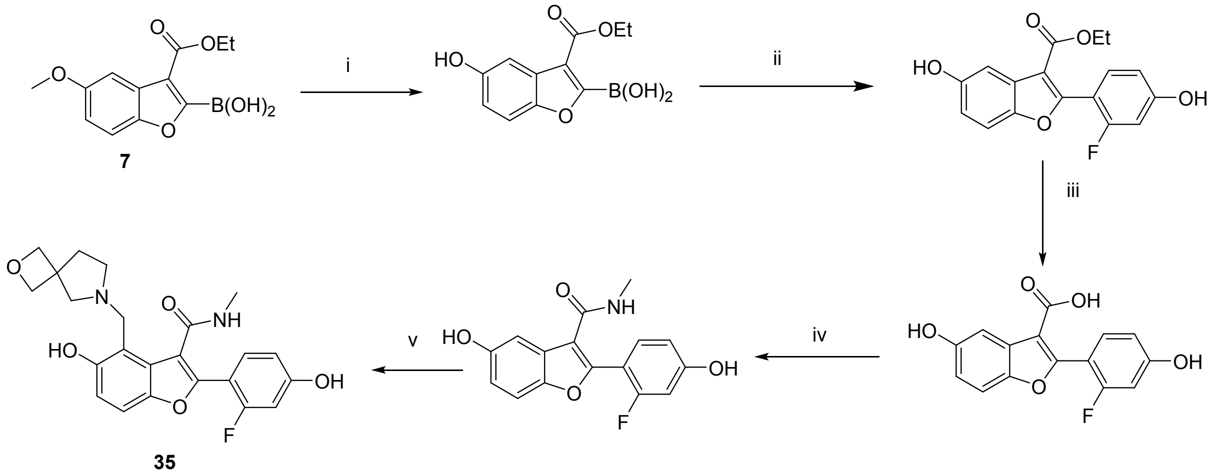

[a]Reagents and conditions: (i) BBr_3, DCM, 0 °C; (ii) 4-bromo-3-fluorophenol, K_3PO_4, Pd(dtbpf)Cl_2, THF, water, 60 °C, N_2; (iii) KOH, water, EtOH, 50 °C; (iv) methanamine hydrochloride, EDCI, HOBt, DIPEA, DMF, 30 °C; (v) formaldehyde, 2-oxa-7-azaspiro[3.4]octane oxalic acid, EtOH, water, 60°C.

(3-(ethoxycarbonyl)-5-hydroxybenzofuran-2-yl)boronic acid

To a solution of (3-(ethoxycarbonyl)-5-methoxybenzofuran-2-yl)boronic acid (16.00 g, 60.60 mmol) in DCM (160 mL) was added BBr_3 (45.54 g, 181.80 mmol, 17.52 mL) drop-wise at 0 °C under N_2. The reaction was stirred at 30 °C for 1h. The reaction solution was quenched by EtOH (5 mL) at 0 °C. The solution was poured into conc. $NaHCO_3$ (500 mL). The mixture was filtered and the filtered cake was washed with H_2O (50 mL x 3), dried in vacuum to give the title compound as a white solid (25.70 g, 102.28 mmol, 84%). LCMS: m/z 251 [M+H]$^+$. ^{1}H NMR (400 MHz, DMSO) δ 9.47 (s, 1H), 9.13 (s, 2H), 7.51 (d, *J*=8.9 Hz, 1H), 7.33 (d, *J*=2.5 Hz, 1H), 6.89 (dd, *J*=2.6, 8.9 Hz, 1H), 4.41 (q, *J*=7.1 Hz, 2H), 1.41 (t, *J*=7.1 Hz, 3H).

ethyl 2-(2-fluoro-4-hydroxyphenyl)-5-hydroxybenzofuran-3-carboxylate

To a solution of (3-(ethoxycarbonyl)-5-hydroxybenzofuran-2-yl)boronic acid (2.00 g, 8.00 mmol), [1,1′-Bis(di-tert-butylphosphino)ferrocene]dichloropalladium(II) (521 mg, 0.8 mmol) and 4-bromo-3-fluorophenol (1.60 g, 8.40 mmol) in THF (80 mL) was added a solution of K_3PO_4 (3.40 g, 16.00 mmol) in H_2O (16 mL) drop-wise at 0°C under N_2. After the mixture changed to a yellow solution, the reaction mixture was stirred at 60°C for 2 h. The mixture was poured into ice-water (w/w = 1/1) (200 mL) and stirred for 5 min. The aqueous phase was extracted with ethyl acetate (150 mL x 3). The combined organic phase was washed with brine (100 mL), dried with anhydrous Na_2SO_4, concentrated in vacuo. The residue was purified by column chromatography (SiO_2, Petroleum ether/Ethyl acetate=10/1 to 2:1) to give the title compound as a yellow solid (2.23 g, 6.67 mmol, 83%). LCMS: m/z =317 [M+H]$^+$. ^{1}H NMR (400 MHz, MeOD-d$_4$) δ 7.48 - 7.46 (m, 1H), 7.40 (d, *J*=2.4 Hz, 1H), 7.35 (d, *J*=8.8 Hz, 1H), 6.84 (dd, *J*=2.4, 8.8 Hz, 1H), 6.73 (dd, *J*=2.4, 8.4 Hz, 1H), 6.63 (dd, *J*=2.4, 12.0 Hz, 1H), 4.28 (q, *J*=7.2 Hz, 2H), 1.29 (t, *J*=7.2 Hz, 3H)

2-(2-fluoro-4-hydroxyphenyl)-5-hydroxybenzofuran-3-carboxylic acid

To a solution of ethyl 2-(2-fluoro-4-hydroxyphenyl)-5-hydroxybenzofuran-3-carboxylate (2.20 g, 6.96 mmol) in EtOH (80 mL) was added a solution of KOH (1.56 g, 27.84 mmol) in H_2O (20 mL) at 25°C. The mixture was stirred at 50°C for 5 h. The reaction mixture was poured into water (30 mL), adjusted to pH=4 with aq.HCl (2M). The mixture was filtered and the filtered cake was washed with Petroleum ether (50 mL) to give the title compound as a yellow solid (1.10 g, 3.09 mmol, 44%). LCMS: m/z =289 $[M+H]^+$. ^1H NMR (400 MHz, DMSO-d_6) δ 12.78 (s, 1H), 10.48 (s, 1H), 9.40 (s, 1H), 7.60 - 7.34 (m, 3H), 6.87 - 6.68 (m, 3H)

2-(2-fluoro-4-hydroxyphenyl)-5-hydroxy-N-methylbenzofuran-3-carboxamide

To a mixture of 2-(2-fluoro-4-hydroxyphenyl)-5-hydroxybenzofuran-3-carboxylic acid (2.0 g, 6.94 mmol) and methanamine hydrochloride (562 mg, 8.33 mmol) in DMF (50 mL) was added HOBt (1.88 g, 13.88 mmol) EDCI (2.00 g, 10.41 mmol) and DIPEA (2.69 g, 20.82 mmol, 3.63 mL) in one portion at 0° C. Then the mixture was stirred at 30°C for 12 h. The mixture was concentrated in vacuo. The residue was poured into ice-H_2O (200 mL). The mixture was filtered and the filtered cake was washed with Petroleum ether (200 mL) to give the title compound as an off-white solid (1.1 g, 3.01 mmol, 43%). LCMS: m/z =302 $[M+H]^+$. ^1H NMR (400 MHz, DMSO-d_6) δ 10.41 (br s, 1H), 9.32 (s, 1H), 7.99 (d, *J*=8.4 Hz, 1H), 7.73 (d, *J*=8.4 Hz, 1H), 7.58 - 7.51(m, 2H), 7.42 (d, *J*=8.8 Hz, 1H), 7.03 (d, J=2.4 Hz, 1H), 6.70 - 6.67 (m, 1H), 2.73 (d, J=4.8 Hz, 3H).

4-((2-oxa-6-azaspiro[3.4]octan-6-yl)methyl)-2-(2-fluoro-4-hydroxyphenyl)-5-hydroxy-N-methylbenzofuran-3-carboxamide formate (35)

To a solution of 2-(2-fluoro-4-hydroxyphenyl)-5-hydroxy-N-methylbenzofuran-3-carboxamide (400 mg, 1.33 mmol) and 2-oxa-7-azaspiro[3.4]octane oxalic acid (293 mg, 1.46 mmol) in EtOH (15 mL) and H_2O (3 mL) was added formaldehyde (161.64 mg, 1.99 mmol, 148.29 µL, 37% solution in water). The reaction mixture was stirred at 60° C for 12 h. The mixture was concentrated in vacuo. The crude product was purified by prep-HPLC (column: Phenomenex Synergi C18 150*25*10µm; mobile phase: [water (0.225%Formic acid)-ACN]; B%: 10%-40%, 11min) to give the title compound as a yellow solid (156 mg, 351.35 µmol, 26%). LCMS: m/z = 427 $[M+H]^+$. ^1H NMR (500 MHz, DMSO) δ 8.27 - 8.20 (m, 2H), 7.45 (t, *J*=8.6 Hz, 1H), 7.38 - 7.35 (m, 1H), 6.82 - 6.67 (m, 3H), 4.49 - 4.44 (m, 4H), 3.93 - 3.91 (m, 2H), 2.83 (s, 2H), 2.69 - 2.67 (m, 3H), 2.60-2.53 (m, 2H) 2.10 - 2.05 (m, 2H). HRMS (ESI): m/z calcd for $C_{23}H_{24}N_2O_5F$ $[M+H^+]$: 427.1669. Found 427.1649.

Scheme S13

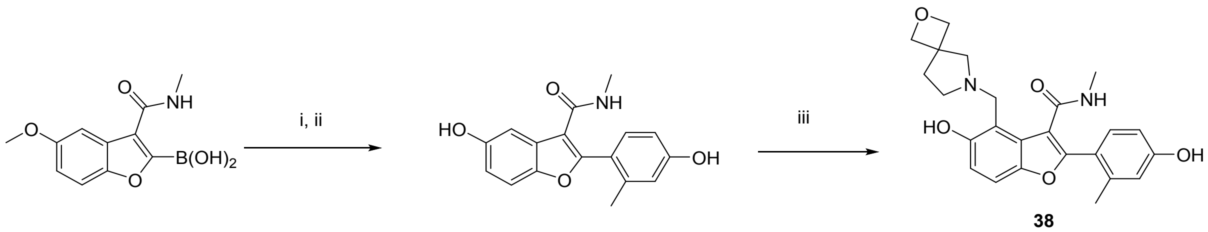

38

[a]*Reagents and conditions:* (i) 4-bromo-3-methyl-phenol, K_2CO_3, Pd(dppf)Cl_2, dioxane, water, 80 °C; (ii) BBr_3, DCM, -78 °C; (iii) formaldehyde, 2-oxa-7-azaspiro[3.4]octane oxalic acid, DIPEA, EtOH, water, 80°C.

5-hydroxy-2-(4-hydroxy-2-methylphenyl)-N-methylbenzofuran-3-carboxamide

[5-methoxy-3-(methylcarbamoyl)benzofuran-2-yl]boronic acid (200 mg, 0.80 mmol), 4-bromo-3-methyl-phenol (150 mg, 0.80 mmol), Potassium carbonate (333 mg, 2.40 mmol) and Pd(dppf)Cl_2 (65

mg, 0.08 mmol) were mixed in dioxane (4mL) and water (1mL) and the reaction mixture was degassed and purged with N_2 and then heated at 80°C for 3 h. The reaction mixture was cooled and quenched with water and then extracted with EtOAc. The combined organic extracts were washed with brine, dried and concentrated. The residue was purified by silica column chromatography (5% MeOH in DCM) to give a residue (100 mg). To a solution of the residue (100 mg, 0.32 mmol) in DCM (5mL) under N_2 was added tribromoborane (241 mg, 0.96 mmol) at -78°C and the mixture was stirred at the same temperature for 15 min and at rt for 2h. Aqueous sat $NaHCO_3$ was added, phases were separated and the aqueous phase was extracted with EtOAc (x3), dried and concentrated. The residue was purified by silica column chromatography (50% EtOAc in heptane) to give the title compound (86 mg, 0.26 mmol, 32%). LCMS: m/z 298 $[M+H]^+$. ^{1}H NMR (400 MHz, DMSO) δ 9.79 (s, 1H), 9.28 (s, 1H), 7.52 - 7.50 (m, 1H), 7.39 - 7.36 (m, 1H), 7.30 - 7.27 (m, 1H), 7.11 (d, *J*=2.4 Hz, 1H), 6.79 - 6.68 (m, 3H), 2.68 (d, *J*=4.7 Hz, 3H), 2.21 (s, 3H).

4-((2-oxa-6-azaspiro[3.4]octan-6-yl)methyl)-5-hydroxy-2-(4-hydroxy-2-methylphenyl)-N-methylbenzofuran-3-carboxamide (38)

To a solution of the corresponding 5-hydroxy-2-(4-hydroxy-2-methylphenyl)-N-methylbenzofuran-3-carboxamide and 2-oxa-7-azaspiro[3.4]octane; oxalic acid (30 mg, 0. 14 mmol) in ethanol (1mL) and water (0.2 mL) was added DIPEA (22 mg, 0.17 mmol) and formaldehyde 37% solution in water (13 mg, 0.16 mmol) and the mixture was stirred at 80°C overnight. Saturated aqueous $NaHCO_3$ was added and the mixture was extracted with EtOAc, dried and concentrated to afford a residue. Purification by prep HPLC (basic, 5-95% ACN) gave the title compound (11 mg, 0.02 mmol, 18%). LCMS: m/z 423 $[M+H]^+$. ^{1}H NMR (400 MHz, DMSO) δ 8.13 (q, *J*=4.5 Hz, 1H), 7.28 (dd, *J*=8.6, 30.3 Hz, 2H), 6.77 - 6.63 (m, 3H), 4.50 - 4.45 (m, 4H), 3.88 (s, 2H), 2.82 (s, 2H), 2.63 (d, *J*=4.7 Hz, 3H), 2.57-2.50 (m, 2H), 2.27 (s, 3H), 2.11-2.05 (m, 2H).

Scheme S14

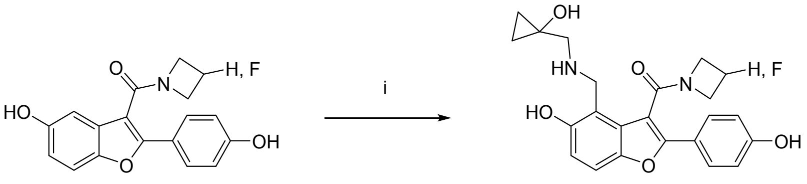

[a]*Reagents and conditions:* (i) Formaldehyde, 1-(aminomethyl)cyclopropanol, EtOH, H$_2$0, 60 °C or microwave 100 °C.

azetidin-1-yl(5-hydroxy-4-((((1-hydroxycyclopropyl)methyl)amino)methyl)-2-(4-hydroxyphenyl)benzofuran-3-yl)methanone formate (39)

A solution of azetidin-1-yl(5-hydroxy-2-(4-hydroxyphenyl)benzofuran-3-yl)methanone (400 mg, 1.29 mmol), 1-(aminomethyl)cyclopropanol (191 mg, 2.19 mmol) and formaldehyde 37% solution in water (177 mg, 2.19 mmol) in EtOH (8 mL) and H_2O (200 µL) was stirred at 60 °C for 40 h. The reaction solution was concentrated under vacuum to give a residue. The residue was purified by preparative HPLC (water (0.225%Formic Acid)-ACN; 10%-40%) to give the title compound (160 mg, 383 µmol, 30 %). LCMS: m/z 409 $[M+H^+]$. ^{1}H NMR (400 MHz, DMSO) δ 8.21 (s, 1H), 7.60 - 7.57 (m, 2H), 7.36 - 7.33 (m, 1H), 6.94 - 6.91 (m, 2H), 6.76 - 6.73 (m, 1H), 4.14 - 4.06 (m, 4H), 3.74-3.58 (br s, 2H), 2.69 (s, 2H), 2.18 - 2.09 (m, 2H), 0.62 - 0.58 (m, 2H), 0.51 - 0.47 (m, 2H).

(3-fluoroazetidin-1-yl)(5-hydroxy-4-((((1-hydroxycyclopropyl)methyl)amino)methyl)-2-(hydroxyphenyl)benzofuran-3-yl)methanone formate (40)

A mixture of 1-(aminomethyl)cyclopropanol (181 mg, 2.07 mmol) , formaldehyde 37% solution in water (252 mg, 3.11mmol) and (3-fluoroazetidin-1-yl)-[5-hydroxy-2-(4-hydroxyphenyl)benzofuran-3-

yl]methanone (680 mg, 2.07 mmol) in ethanol (10 mL) and water (2 mL) were heated under microwave conditions at 100 ^{0}C for 1 h. Reaction was diluted with EtOAc then water added. EtOAc was separated and aqeous extracted with EtOAc (x3). Extracts were combined and dried over Na_2SO_4. Purification by silica column chromatography (EtOAc 100% gradient elution with Heptane) to give 174 mg of a yellow gum. The yellow gum was purified by acidic prep. HPLC to give the title compound. (74 mg, 0.153 mmol, 7%). LCMS: m/z 428 $[M+H]^+$.^{1}H NMR (400 MHz, MeOD) δ 8.47 (s, 1H), 7.64 - 7.61 (m, 2H), 7.54 - 7.51 (m, 1H), 7.01 - 6.98 (m, 3H), 5.34-5.10 (m, 1H), 4.60-4.39 (m, 3H), 4.33 - 4.17 (m, 1H), 4.03 - 3.93 (m, 1H), 3.64 (dd, *J*=10.7, 24.3 Hz, 1H), 3.18 - 3.17 (m, 2H), 0.93 - 0.74 (m, 4H). HRMS (ESI): m/z calcd for $C_{23}H_{24}N_2O_5F$ $[M+H^+]$: 427.1669. Found 427.1685.

Scheme S15

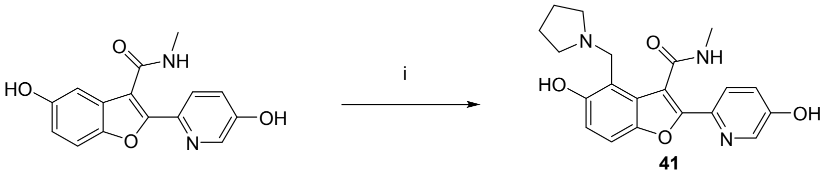

^{a}Reagents and conditions: (i) Formaldehyde, pyrrolidine, EtOH, water, 70 °C.

5-Hydroxy-2-(5-hydroxypyridin-2-yl)-N-methyl-4-(pyrrolidin-1-ylmethyl)benzofuran-3-carboxamide formate (41)

To a suspension of 5-hydroxy-2-(5-hydroxy-2-pyridyl)-N-methyl-benzofuran-3-carboxamide (230 mg, 0.80 mmol) in THF (8 mL) and water (1.5 mL), pyrrolidine (63 mg, 0.89 mmol) and formaldehyde 37% solution in water (85 mg, 1.05 mmol) were successively added. The mixture was stirred at 70°C overnight. Saturated aqueous $NaHCO_3$ was added and the mixture was extracted with EtOAc, dried and concentrated to afford a residue. Purification by prep HPLC (acidic, 5-95% ACN) gave the title compound (41 mg, 0.09 mmol, 11%) LCMS: m/z 368 $[M+H]^+$. ^{1}H NMR (400 MHz, DMSO) δ 8.46 (q, *J*=4.5 Hz, 1H), 8.21 - 8.17 (m, 2H), 7.69 - 7.66 (m, 1H), 7.42 - 7.39 (m, 1H), 7.29 (dd, *J*=2.8, 8.6 Hz, 1H), 6.81 - 6.78 (m, 1H), 3.98 (s, 2H), 2.78 (d, *J*=4.7 Hz, 3H), 2.66 (s, 4H), 1.80 - 1.79 (m, 4H).

Scheme S16

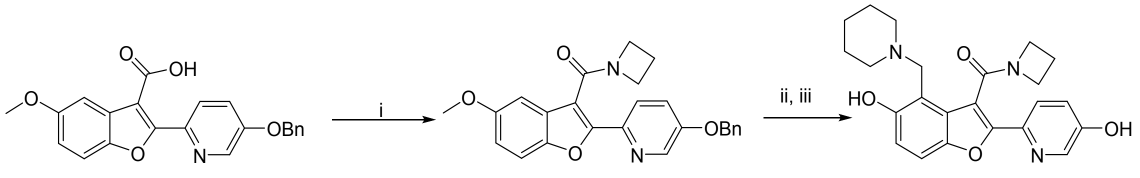

^{a}Reagents and conditions: (i) HATU, DIPEA, azetidine, DMF, rt; (ii) BBr_3, THF, 0°; (iii) formaldehyde, piperidine, AcOH, THF, water, reflux.

Azetidin-1-yl(2-(5-(benzyloxy)pyridin-2-yl)-5-methoxybenzofuran-3-yl)methanone

A mixture of 2-(5-(benzyloxy)pyridin-2-yl)-5-methoxybenzofuran-3-carboxylic acid (0.10 g, 0.266 mmol), HATU (0.11 g, 0.293 mmol), *N,N*-diisopropylethylamine (0.07 g, 0.532 mmol) and azetidine (0.016 g, 0.319 mmol) in DMF (3 mL) was stirred at rt for 5 h. Water was then added and the mixture was extracted with EtOAc. The combined organic extracts were washed with brine, dried and concentrated under reduced pressure. The residue was purified by column chromatography over silica gel using 50-70% EtOAc in heptanes as eluent to give the title compound (0.09 g, 0.194 mmol, 73%). LCMS: m/z 415 $[M+H]^+$. ^{1}H NMR (400 MHz, MeOD) δ 8.49 (d, *J*=2.8 Hz, 1H), 7.95 - 7.92 (m, 1H),

7.59 (dd, J=2.7, 8.8 Hz, 1H), 7.53 - 7.34 (m, 6H), 7.08 (d, J=2.5 Hz, 1H), 7.01 (dd, J=2.6, 9.0 Hz, 1H), 5.28 (s, 2H), 4.29 (t, J=7.8 Hz, 2H), 3.88 - 3.87 (m, 5H), 2.34 - 2.26 (m, 2H).

azetidin-1-yl(5-hydroxy-2-(5-hydroxypyridin-2-yl)-4-(piperidin-1-ylmethyl)benzofuran-3-yl)methanone (42)

A mixture of azetidin-1-yl(2-(5-(benzyloxy)pyridin-2-yl)-5-methoxybenzofuran-3-yl)methanone (0.09 g, 0.21 mmol) and tribromoborane (0.217 g, 0.86 mmol) in THF (1 mL) was stirred at 0°C to rt overnight. Saturated aqueous ammonium chloride was added and the mixture was extracted with EtOAc. The combined organic extracts were dried over magnesium sulfate and concentrated under reduced pressure. The residue was suspended in THF (1 mL) and then water (0.2 mL), piperidine (18 mg, 0.21 mmol), acetic acid (0.05 mL) and formaldehyde 37% solution in water (18 mg, 0.24 mmol) were added and the mixture was stirred at reflux for 2 h. After cooling to ambient temperature, saturated aqueous $NaHCO_3$ was added and the mixture was extracted with EtOAc. The organic extracts were then combined and concentrated under reduced pressure. The residue was purified by mass directed HPLC, 5-95% MeCN, basic method to give the title compound (0.021 g, 0.05 mmol, 23%). LCMS: m/z 408 $[M+H]^+$. ^1H NMR (400 MHz, DMSO) δ 8.26 (d, J=2.8 Hz, 1H), 7.73 - 7.70 (m, 1H), 7.38 - 7.28 (m, 2H), 6.79 - 6.76 (m, 1H), 4.07 (bs, 2H), 3.78 - 3.76 (m, 4H), 2.45 (bs, 4H), 2.17 (bs, 2H), 1.54 - 1.42 (m, 6H).

Scheme S17

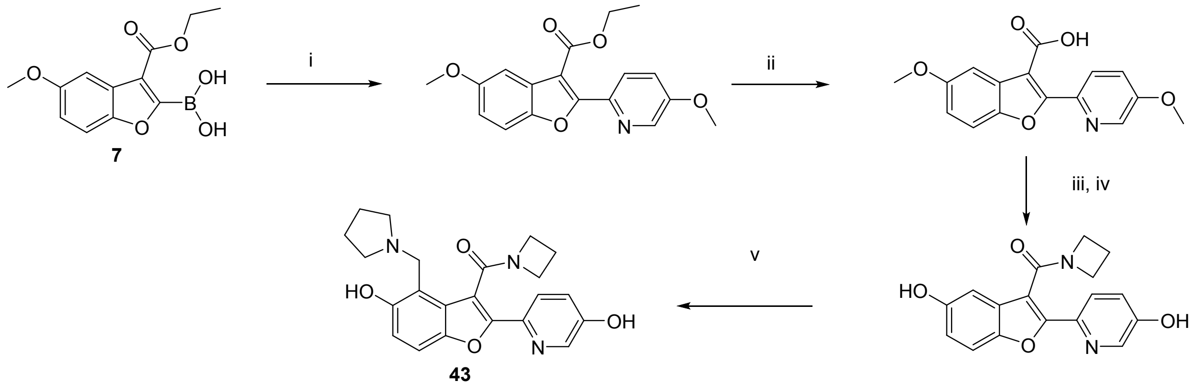

[a]*Reagents and conditions:* (i) 2-bromo-5-methoxypyridine, Pd(dppf)Cl_2 , K_2CO_3 , 1,4-dioxane, water, 60°C; (ii) KOH, EtOH, water, 50°C; (iii) azetidine hydrochloride, HOBt, DIPEA, EDCI 0 °C to 25 °C; (iv) BBr_3, DCM, -70 °C; (v) formaldehyde, pyrollidine, EtOH, water, 75°C.

ethyl 5-methoxy-2-(5-methoxypyridin-2-yl)benzofuran-3-carboxylate

To a solution of (3-(ethoxycarbonyl)-5-methoxybenzofuran-2-yl)boronic acid (30.00 g, 113.62 mmol) and 2-bromo-5-methoxypyridine (22.00 g, 117.03 mmol) in 1,4-dioxane (200 mL) and H_2O (50 mL) were added Pd(dppf)Cl_2 (4.16 g, 5.68 mmol) and K_2CO_3 (31.41 g, 227.24 mmol) portionwise while the temperature was kept at 25°C under N_2 to give a yellow solution. The reaction mixture was stirred at 60°C for 2 h. The mixture was poured into water (50 mL) and stirred for 5 min. The aqueous phase was extracted with ethyl acetate (40 mL x 3). The combined organic phases were washed with brine (30 mL), dried with anhydrous Na_2SO_4, filtered and concentrated under vacuum. The residue was purified by column chromatography (SiO_2, Petroleum ether/Ethyl acetate=10/1 to 2:1) to give the title compound (17.00 g, 51.93 mmol, 45%) as a white solid. ^1H NMR (400 MHz, $CDCl_3$) δ 8.47 (d, J=2.8 Hz, 1H), 8.18 (d, J=8.8 Hz, 1H), 7.55-7.42 (m, 2H), 7.28-7.26 (m, 1H), 6.98-6.95 (m, 1H), 4.41 (q, J=7.2 Hz, 2H), 3.91 (s, 3H), 3.7 (s, 3H), 1.41 (t, J=7.2 Hz, 3H).

5-methoxy-2-(5-methoxypyridin-2-yl)benzofuran-3-carboxylic acid

To a mixture of ethyl 5-methoxy-2-(5-methoxypyridin-2-yl)benzofuran-3-carboxylate (10.00 g, 30.55 mmol) and H_2O (10 mL) and EtOH (100 mL) was added KOH (6.86 g, 122.20 mmol) in one portion at

25°C. The mixture was stirred at 50°C for 3 h. The mixture was poured into aq. $NaHCO_3$ (1M, 200 mL) to give a precipitate. The solid was collected and washed with MeOH (20 mL) to give compound the title compound (9.00 g, 30.10 mmol, 98%) as a yellow solid. LCMS: m/z 300 $[M+H]^+$. ^1H NMR (400 MHz, DMSO) δ 8.61 (d, *J*=2.4 Hz, 1H), 8.24 (d, *J*=8.8 Hz, 1H), 7.85 (m, 1H), 7.76 (d, *J*=2.4 Hz, 1H), 7.64 (d, *J*=8.8 Hz, 1H), 7.08-7.05 (m, 1H), 3.99 (s, 3H), 3.84 (s, 3H).

azetidin-1-yl(5-hydroxy-2-(5-hydroxypyridin-2-yl)benzofuran-3-yl)methanone

To a mixture of 5-methoxy-2-(5-methoxypyridin-2-yl)benzofuran-3-carboxylic acid (2.00 g, 6.68 mmol) and azetidine hydrochloride (572 mg, 6.11 mmol, 672 µL) in DMF (20 mL) were added HOBt (1.81 g, 13.36 mmol), DIPEA (4.32 g, 33.40 mmol, 5.84 mL) and EDCI (2.31 g, 12.02 mmol) in one portion at 0˚C, then the mixture was stirred at 25°C for 12 h. The mixture was concentrated under vacuum. The residue was poured onto ice-H_2O (10 mL) and stirred for 1 h. The aqueous phase was extracted with ethyl acetate (50 mL x 5). The combined organic phases were washed with brine (100 mL), dried over anhydrous Na_2SO_4, filtered and concentrated under vacuum to give a yellow solid (1.8 g). To a mixture of the yellow solid (1.00 g, 2.96 mmol) in DCM (100 mL) was added BBr_3 (7.40 g, 29.55 mmol, 2.85 mL) in one portion at -70°C under N_2. The mixture was stirred at 40°C for 16 h. The reaction mixture was quenched with aqueous $NaHCO_3$ (1 M, 200 mL) and PE (100 mL) was added. The mixture was stirred for 5 min at 25°C and filtered. The filtered cake was washed with PE (50 mL) to give the title compound (710 mg, 2.28 mmol, 60%) as a light yellow solid. LCMS: m/z 311 $[M+H]^+$.^1H NMR (400MHz, MeOD) δ 8.36 (d, *J*=2.4 Hz, 1H), 8.12-8.05 (m, 1H), 7.67-7.60 (m, 1H), 7.46 (d, *J*=8.8 Hz, 1H), 7.03-6.91 (m, 2H), 4.32-4.00 (m, 4H), 2.42-2.28 (m, 2H).

azetidin-1-yl(5-hydroxy-2-(5-hydroxypyridin-2-yl)-4-(pyrrolidin-1-ylmethyl)benzofuran-3-yl)methanone formate (43)

To a solution of azetidin-1-yl(5-hydroxy-2-(5-hydroxypyridin-2-yl)benzofuran-3-yl)methanone (250 mg, 0.80 mmol) and formaldehyde 37% solution in water (130 mg, 1.61 mmol, 119.98 µL) in H_2O (2 mL) and EtOH (10 mL) was added pyrrolidine (68 mg, 966 µmol, 80.8 µL). The reaction mixture was stirred at 75°C for 16 h. The mixture was filtered and concentrated under vacuum to get the residue. The residue was purified by prep-HPLC (column: Boston Green ODS 150*30 5u; mobile phase: [water (0.225%Formic acid)-ACN]; B%: 5%-35%,10min) to give the title compound (168 mg, 427 µmol, 53%) as an off-white solid. LCMS: m/z 394 $[M+H]^+$.^1H NMR (500 MHz, MeOD) δ 8.52 (s, 1H), 8.29 (d, *J*=2.4 Hz, 1H), 7.82 (d, *J*=9.0 Hz, 1H), 7.50 (d, *J*=8.9 Hz, 1H), 7.33 (dd, *J*=2.8, 8.6 Hz, 1H), 6.97 (d, *J*=8.9 Hz, 1H), 4.50 (s, 2H), 4.25 (t, *J*=7.9 Hz, 2H), 3.76 (t, *J*=7.5 Hz, 2H), 3.41 (t, *J*=6.6 Hz, 4H), 2.27 - 2.18 (m, 2H), 2.14 - 2.09 (m, 4H). HRMS (ESI): m/z calcd for $C_{22}H_{24}N_3O_4$ $[M+H^+]$: 394.1767. Found 394.1786.

Scheme S18

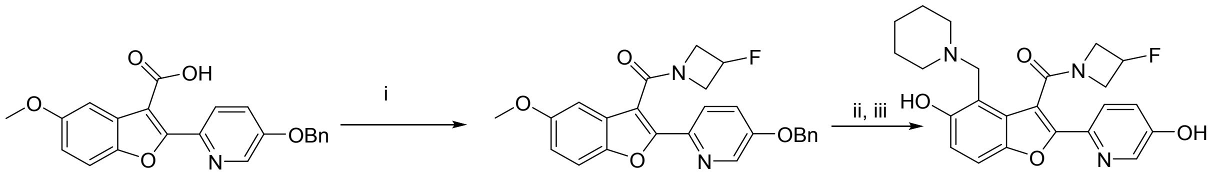

9 44

[a]Reagents and conditions: (i) HATU, DIPEA, 3-fluoroazetidine hydrochloride,DMF, rt; (ii) BBr_3, THF, -78 °C to rt; (iii) formaldehyde, piperidine, AcOH, THF, water, 70 °C.

(2-(5-(benzyloxy)pyridin-2-yl)-5-methoxybenzofuran-3-yl)(3-fluoroazetidin-1-yl)methanone

To a suspension of 2-(5-benzyloxy-2-pyridyl)-5-methoxy-benzofuran-3-carboxylic acid (200 mg, 0.53 mmol) and 3-fluoroazetidine hydrochloride (62 mg, 0.55 mmol) in DMF (3mL) were successively added HATU (222 mg, 0.58 mmol) and DIPEA (206 mg, 1.59 mmol) and the mixture was stirred

overnight at rt. Water was added and the mixture was extracted with EtOAc (x3), washed with brine, dried and concentrated. The residue was purified by silica (5-10% MeOH in DCM) to give the title compound (110 mg, 0.24 mmol, 45%). LCMS: m/z 433 [M+H$^+$]. ^{1}H NMR (400 MHz, DMSO) δ 8.52 (d, *J*=2.8 Hz, 1H), 7.95 - 7.91 (m, 1H), 7.69 - 7.58 (m, 2H), 7.52 - 7.37 (m, 5H), 7.04 - 6.99 (m, 2H), 5.30 - 5.28 (m, 3H), 4.50 - 4.40 (m, 1H), 4.20 - 3.97 (m, 2H), 3.84-3.67 (m, 4H).

(3-fluoroazetidin-1-yl)(5-hydroxy-2-(5-hydroxypyridin-2-yl)-4-(piperidin-1-ylmethyl)benzofuran-3-yl)methanone formate (44)

To a solution of [2-(5-benzyloxy-2-pyridyl)-5-methoxy-benzofuran-3-yl]-(3-fluoroazetidin-1-yl)methanone (250 mg, 0.57 mmol) in DCM (6 mL) under N_2 was added tribromoborane (724 mg, 2.89 mmol) at -78°C and the mixture was slowly warmed up and stirred at rt for overnight. Aqueous sat $NaHCO_3$ was added, phases were separated and the aqueous phase was extracted with EtOAc (x3), dried and concentrated to give a residue. Purification by silica chromatography (5% MeOH in DCM) gave an impure residue (175 mg, 0.47 mmol). To a solution of this residue (50 mg, 0.15 mmol) and piperidine (14 mg, 0.16 mmol) in THF (1 mL) was added formaldehyde 37% solution in water (16 mg, 0.19 mmol) and the mixture was stirred at 70 °C overnight. Saturated aqueous $NaHCO_3$ was added and the mixture was extracted with EtOAc (x 3), dried and concentrated to afford a residue. Purification by prep HPLC (acidic, 5-95% ACN) gave the title compound (23 mg, 0.04 mmol, 30%). LCMS: m/z 426 [M+H]$^+$. ^{1}H NMR (400 MHz, DMSO) δ 8.25 (d, *J*=2.8 Hz, 1H), 8.17 (s, 0.5H), 7.77 - 7.74 (m, 1H), 7.40 - 7.31 (m, 2H), 6.82 - 6.78 (m, 1H), 5.52- 5.30 (m, 1H), 4.46 - 4.38 (m, 1H), 4.17 - 3.98 (m, 3H), 3.76 (s, 2H), 2.49-2.40 (m, 4H), 1.53 - 1.44 (m, 6H).

Scheme S19

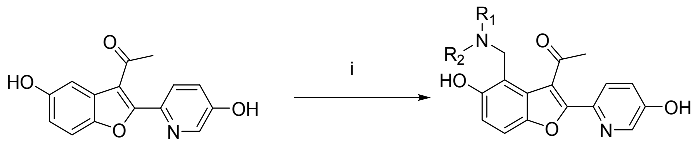

^{a}*Reagents and conditions:* (i) formaldehyde, 2-oxa-6-azaspiro[3.4]octane oxalate, DIPEA, EtOH, water, 70°C, or formaldehyde, pyrollidine, EtOH, water, 70°C.

1-(4-((2-oxa-6-azaspiro[3.4]octan-6-yl)methyl)-5-hydroxy-2-(5-hydroxypyridin-2-yl)benzofuran-3-yl)ethan-1-one formate (45)

To a solution of 1-(5-hydroxy-2-(5-hydroxypyridin-2-yl)benzofuran-3-yl)ethan-1-one (40 mg, 0.15 mmol) and 2-oxa-6-azaspiro[3.4]octane oxalate (33 mg, 0.16 mmol) in ethanol (1 mL) and water (0.2 mL) was added DIPEA (29 mg, 0.22 mmol) and formaldehyde 37% solution in water (16 mg, 0.19 mmol), and the reaction mixture was stirred at 70°C overnight. Saturated $NaHCO_3$ was added and the aqueous layer was extracted with EtOAc, evaporated, and purified by HPLC (5-95% MeCN/water, acidic method) to afford the title compound (6 mg, 0.01 mmol, 9%). LCMS: m/z 395 [M+H]$^+$. ^{1}H NMR (400 MHz, DMSO) δ 8.21 (d, *J*=2.8 Hz, 1H), 8.18 (s, 1H), 7.79 (d, *J*=8.8 Hz, 1H), 7.38 - 7.31 (m, 2H), 6.88 (d, *J*=8.9 Hz, 1H), 4.41 (s, 4H), 3.61 (s, 2H), 2.61 (s, 2H), 2.42 (s, 3H), 2.40 - 2.36 (m, 2H), 2.06 - 1.99 (m, 2H). HRMS (ESI): m/z calcd for $C_{22}H_{23}N_2O_5$ [M+H$^+$]: 395.1607. Found 395.1643.

1-(5-hydroxy-2-(5-hydroxypyridin-2-yl)-4-(pyrrolidin-1-ylmethyl)benzofuran-3-yl)ethan-1-one formate (46)

To a solution of 1-[5-hydroxy-2-(5-hydroxy-2-pyridyl)benzofuran-3-yl]ethanone (40 mg, 0.14 mmol) in THF (1mL) and water (0.2mL) , pyrrolidine (11 mg, 0.16 mmol) and formaldehyde 37% solution in water (15 mg, 0.19 mmol) were successively added. The mixture was stirred at 70°C for

3h. Saturated aqueous $NaHCO_3$ was added and the mixture was extracted with EtOAc, dried and concentrated to afford a residue. Purification by prep HPLC (acidic, 5-95% ACN) gave the title compound (17 mg, 0.04 mmol, 27%). LCMS: m/z 353 $[M+H]^+$. ^1H NMR (400 MHz, DMSO) δ 8.21 - 8.17 (m, 2H), 7.80 - 7.77 (m, 1H), 7.37 - 7.31 (m, 2H), 6.87 - 6.84 (m, 1H), 3.67 (s, 2H), 2.50 (s, 3H), 2.40 - 2.39 (m, 4H), 1.68-1.63 (m, 4H).

Scheme S20

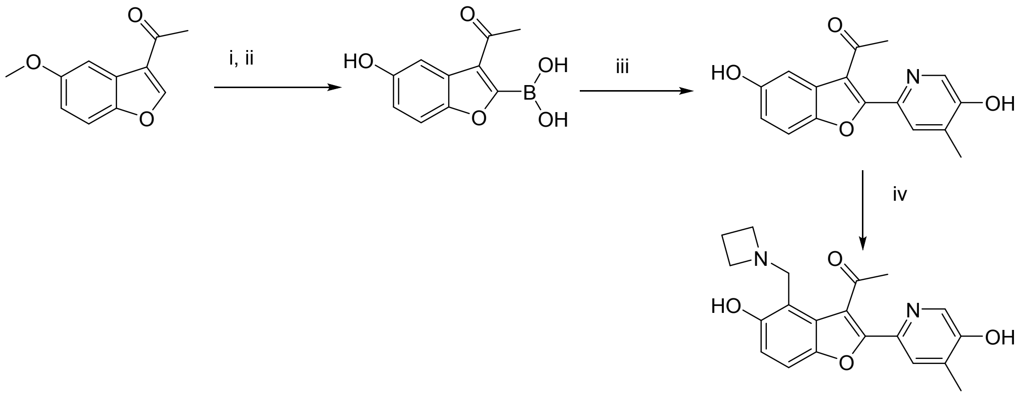

[a]Reagents and conditions: (i) $B(OMe)_3$, LDA, -78 °C 2-bromo-5-methoxypyridine, N_2; (ii) BBr_3, DCM, 0 °C to 20 °C; (iii) 6-bromo-4-methyl-pyridin-3-ol, 1,1'-Bis(di-tert-butylphosphino) ferrocenepalladium chloride, K_3PO_4, 80 °C, microwave, N_2; (iv) formaldehyde, azetidine, AcOH, EtOH, 80°C.

(3-acetyl-5-hydroxybenzofuran-2-yl)boronic acid

To a solution of 1-(5-methoxybenzofuran-3-yl)ethanone (17.00 g, 89.38 mmol) and $B(OMe)_3$ (37.15 g, 357.52 mmol) in THF (200 mL) was added LDA (2 M, 187.70 mL) dropwise at -78 °C under N_2. After addition, the solution was stirred at -78 °C for 2 h. The reaction solution was poured into aq. HCl to give a suspension. Then the resulting suspension was filtered, the filtered cake was washed with water and dried in vacuum to afford a light yellow solid (17 g). To a solution of the light yellow solid (13.00 g, 55.55 mmol) in DCM (200 mL) was added BBr_3 (37.96 g, 151.51 mmol) dropwise at 0 °C. After addition, the solution was stirred at 20 °C for 2 h. The solution was poured into ice water to give a suspension. Then above suspension was filtered, the filtered cake was washed with water and concentrated in vacuum to give a residue. The residue was purified by Prep-HPLC (water (0.225% formic acid)-ACN; 20%-45%) to afford the title compound (10 g, 41.59 mmol, 63 %) as a red solid. LCMS LCMS: m/z 220.9 [M+H]. ^1H NMR **(**400 MHz, DMSO) δ 9.44 (br s, 2H), 7.49 (d, *J*=8.9 Hz, 1H), 7.39 (d, *J*=2.4 Hz, 1H), 6.87 (dd, *J*=2.4, 8.8 Hz, 1H), 2.64 (s, 3H)

1-(5-hydroxy-2-(5-hydroxy-4-methylpyridin-2-yl)benzofuran-3-yl)ethan-1-one

A mixture of potassium phosphate tribasic (144 mg, 0.68 mmol) 1,1'-Bis(di-tert-butylphosphino) ferrocenepalladium chloride (7 mg, 0.01 mmol) 6-bromo-4-methyl-pyridin-3-ol (42 mg, 0.22 mmol), (3-acetyl-5-hydroxy-benzofuran-2-yl)boronic acid (50 mg, 0.22 mmol) in THF (1.5 mL) and water (1.5mL) was purged with N_2 and heated in the microwave at 80°C . Once the reaction completed by TLC water (10 mL) was added and the reaction extracted with ethylacetate (10 mL x 2), washed with brine (10 mL x 2), dried over $MgSO_4$ and concentration to dryness. The crude was then purified by silica flash column chromatography eluting (10 % Ethylacetate:Hexane) to give the title compound (20 mg, 0.067 mmol, 29%). LCMS: m/z 284 $[M+H]^+$. ^1H NMR (500 MHz, DMSO) δ 10.47 (br s, 1H), 9.41 (br s, 1H), 8.23 (s, 1H), 7.77 (s, 1H), 7.47 - 7.44 (m, 1H), 7.28 (d, *J*=2.4 Hz, 1H), 6.83 (dd, *J*=2.6, 8.9 Hz, 1H), 2.40 (s, 3H), 2.26 (s, 3H).

1-(4-(azetidin-1-ylmethyl)-5-hydroxy-2-(5-hydroxy-4-methylpyridin-2-yl)-2,3-dihydrobenzofuran-3-yl)ethan-1-one formate (47)

To a solution of 1-[5-Hydroxy-2-(5-hydroxy-4-methyl-2-pyridyl)benzofuran-3-yl]ethanone (31 mg, 0.11 mmol), azetidine (16 mg, 0.28 mmol) and formaldehyde 37% solution in water (22 mg, 0.28 mmol) in ethanol (0.70 mL), was added one drop of a solution of acetic acid (one drop acetic acid dissolved into 1 mL ethanol) and the mixture was heated at 80°C overnight. Saturated aqueous $NaHCO_3$ was added and the mixture was extracted with EtOAc and evaporated to afford a residue. The residue was purified by preparative HPLC using 5- 95% gradient elution of 0.1% formic acid: ACN, mixture solvent, desired fraction evaporated to give the title compound (13 mg, 0.03 mmol, 30%). LCMS: m/z 353 $[M+H]^+$. ^1H NMR (500 MHz, MeOD) δ 8.46 (s, 1H), 8.16 (s, 1H), 7.76 (s, 1H), 7.53 (d, *J* = 9.0 Hz, 1H), 7.01 (d, *J* = 8.9 Hz, 1H), 4.55 (s, 2H) 4.32 (dd, *J* = 7.9, 7.9 Hz, 4H), 2.59 - 2.50 (m, 2H), 2.45 - 2.41 (m, 3H), 2.34 (s, 3H). HRMS (ESI): m/z calcd for $C_{20}H_{21}N_2O_4$ $[M+H^+]$: 353.1501. Found 353.1503.

HPLC traces for *in vivo* compounds

Compound 1- TAM16 synthesis described in a previous paper

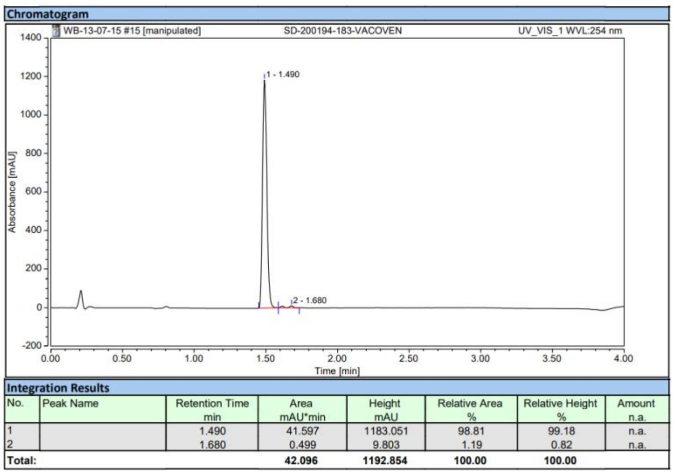

Integration Results

No.	Peak Name	Retention Time min	Area mAU*min	Height mAU	Relative Area %	Relative Height %	Amount n.a.
1		1.490	41.597	1183.051	98.81	99.18	n.a.
2		1.680	0.499	9.803	1.19	0.82	n.a.
Total:			42.096	1192.854	100.00	100.00	

Compound 13

Sample Description 200282-073-001

Method 3-microtof-2 Identify Compounds BASIC LCMS Pos 5-95.m

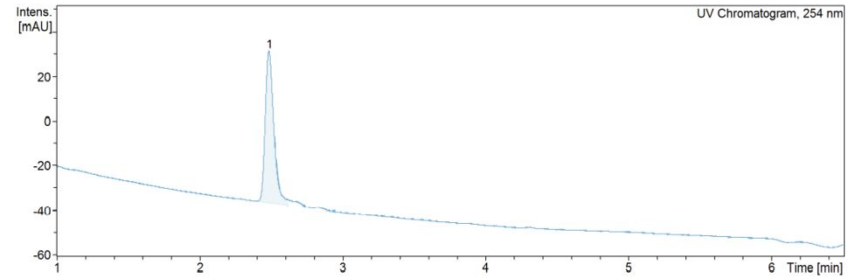

#	RT [min]	Area	Area Frac. %
1	2.5	280.39	100.000
2	2.5	5798509.50	100.000

SmartFormula Settings

Tolerance	mSigma Limit	Electron Conf.
2 mDa	20	even

Bruker Compass DataAnalysis 4.2 printed: 11/03/2016 09:49:55 Page 1 of 2

Analysis Name D:\Data\malcolm\200282-073-001_2439_RA7_01_2601.d

Compound 16

Chromatogram

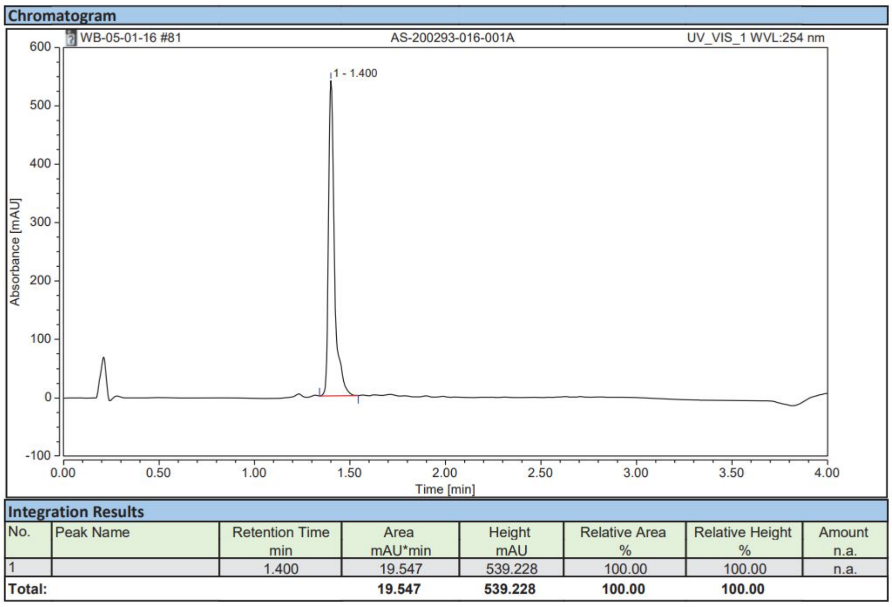

Integration Results

No.	Peak Name	Retention Time min	Area mAU*min	Height mAU	Relative Area %	Relative Height %	Amount n.a.
1		1.400	19.547	539.228	100.00	100.00	n.a.
**Total:**			**19.547**	**539.228**	**100.00**	**100.00**	

Compound 6

Analytical Studio Reviewer Report

Sample Name: jh-200238-146-2639
Location: 1,1:C,6
Acquired: 23-Oct-15 1:22 PM
Filename: jh-200238-146-26395166.D
Instrument: 5-90 MECN pos EZX-8.M
Sample Info Walkup method: '2_5-90_POS_EZX-8min'

		Area %			
Peak #	Time	TIC(+)	UV254	UV210	Found
1	2.275	0.0	100.0	100.0	NA
2	2.316	100.0	0.0	0.0	NA

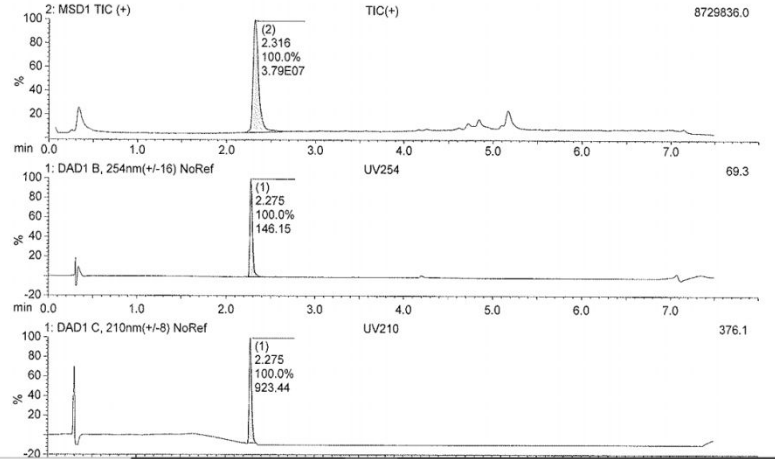

Compound 26

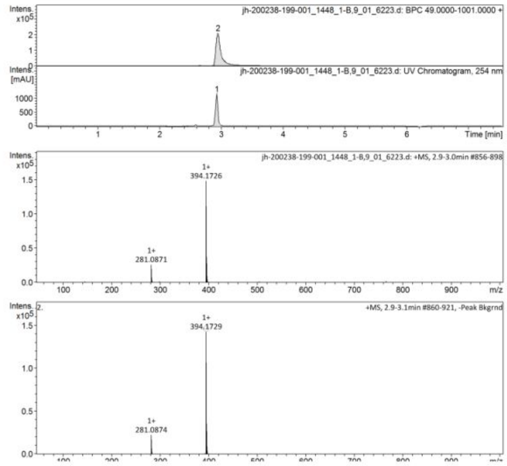

#	RT [min]	Area	Area Frac. %	Chromatogram
1	2.9	3145.8	100.00	UV Chromatogram, 254 nm
2	2.9	1156720.3	100.00	BPC 49.0000-1001.0000 +

Compound 35

Sample-ID DU1_A120
Submitter Nicola Caldwell
Analysis Name DU1_A120_9532_RB3_01_10252.d
Sample Description

Station Microtof-2
Supervisor System Administrator
Acquisition Date 29/11/2016 17:31:34
Method 1-microtof-2 Identify Compounds LCMS Pos 5-95.m

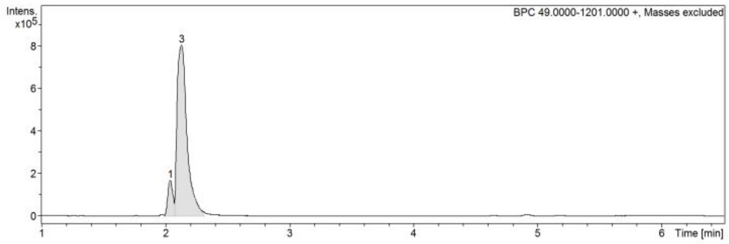

Sample Description

Method 1-microtof-2 Identify Compounds LCMS Pos 5-95.m

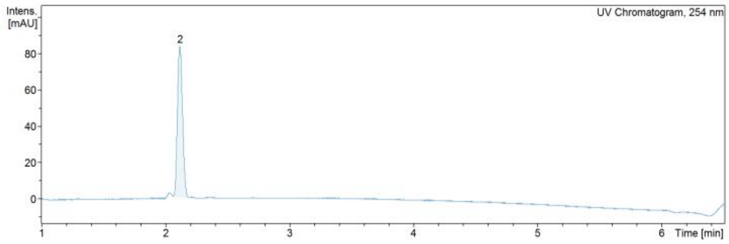

#	RT [min]	Area Frac. %	Chromatogram
1	2.0	9.851	BPC 49.0000-1201.0000 +, Masses excluded
2	2.1	100.000	UV Chromatogram, 254 nm
3	2.1	90.149	BPC 49.0000-1201.0000 +, Masses excluded

Compound 36

Sample Name: CM-200296-071-001
Location: 1,1:F,7
Acquired: 19-Feb-16 2:18 PM
Filename: CM-200296-071-0011154.D
Instrument: 5-90 MECN pos EZX-8.M
Sample Info Walkup method: '2_5-90_POS_EZX-8min'

		Area %		
Peak #	Time	TIC(+)	UV254	Found
1	2.407	0.0	100.0	NA
2	2.454	89.5	0.0	NA
3	5.241	10.5	0.0	NA

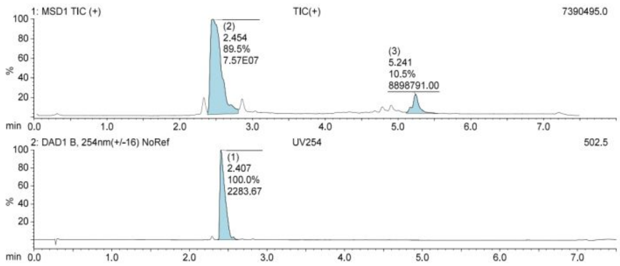

Compound 40

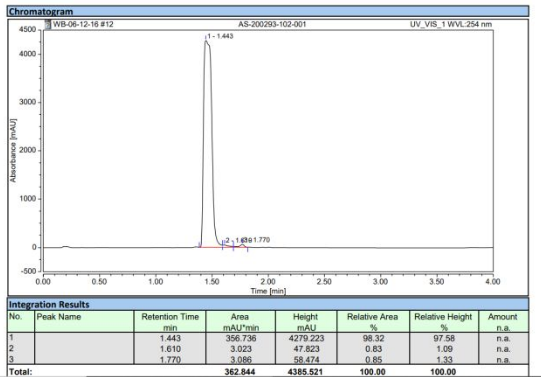

Integration Results

No.	Peak Name	Retention Time min	Area mAU*min	Height mAU	Relative Area %	Relative Height %	Amount n.a.
1		1.443	356.736	4279.223	98.32	97.58	n.a.
2		1.610	3.023	47.823	0.83	1.09	n.a.
3		1.770	3.086	58.474	0.85	1.33	n.a.
**Total:**			**362.844**	**4385.521**	**100.00**	**100.00**	

Compound 43

Sample Description 200282-106-002

Method 1-microtof-2 Identify Compounds LCMS Pos 5-95.m

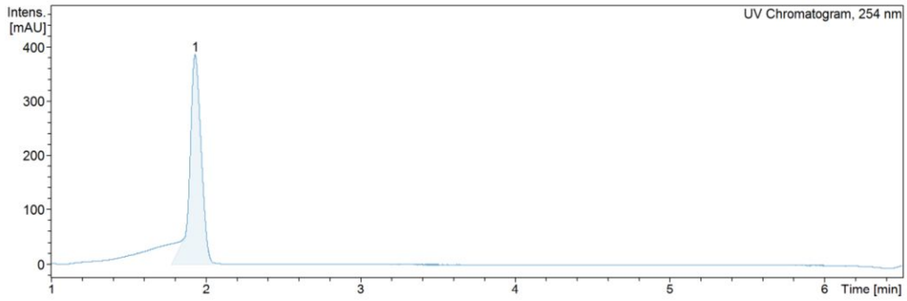

#	RT [min]	Area Frac. %	Chromatogram
1	1.9	100.00	UV Chromatogram, 254 nm
2	1.9	100.00	BPC 49.0000-1201.0000 +, Masses excluded

Compound 45

Sample Name: jh-200333-054-002
Location: 1,2:A,2
Acquired: 27-Apr-16 1:27 PM
Filename: jh-200333-054-0025532.D
Instrument: 5-90 MECN pos EZX-8.M
Sample Info Walkup method: '2_5-90_POS_EZX-8min'

Peak #	Time	Area % TIC(+)	Area % UV254	Found
1	3.227	0.0	100.0	NA
2	3.362	100.0	0.0	NA

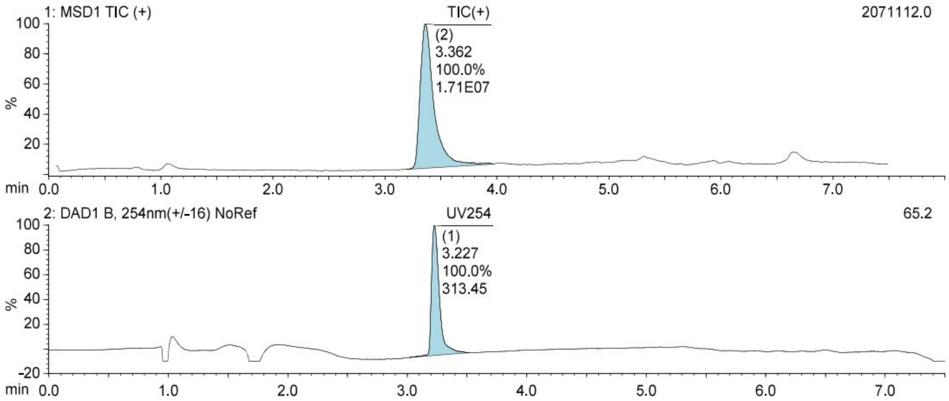

Compound 12

Analytical Studio Reviewer Report

Sample Name: jh-200333-060-001
Location: 1,1:B,9

Acquired: 20-May-16 1:19 PM
Filename: jh-200333-060-0016150.D

Instrument: 5-90 MECN pos EZX-8.M
Sample Info Walkup method: '2_5-90_POS_EZX-8min'

		Area %			
Peak #	Time	TIC(+)	UV254	UV210	Found
1	2.346	0.0	100.0	100.0	NA
2	2.389	100.0	0.0	0.0	NA

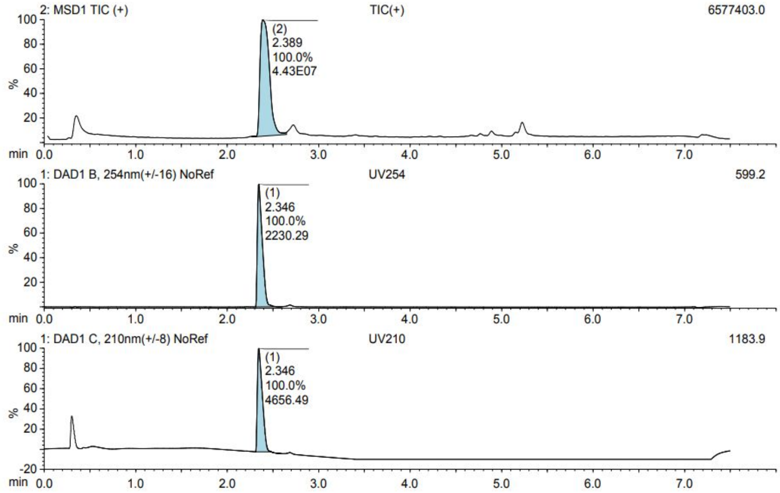

Supplement: Supplementary file 1 — jm1c01586_si_001.pdf [file jm1c01586_si_001.pdf]
